# The chromatin remodeler SMARCA5 binds to d-block metal supports: Characterization of affinities by IMAC chromatography and QM analysis

**DOI:** 10.1371/journal.pone.0309134

**Published:** 2024-10-07

**Authors:** Prokopis C. Andrikopoulos, Pavel Čabart

**Affiliations:** 1 BIOCEV, Institute of Biotechnology of the Czech Academy of Sciences, Vestec, Czechia; 2 BIOCEV, 1^st^ Faculty of Medicine, Charles University, Vestec, Czechia; 3 Institute of Experimental Medicine of the Czech Academy of Sciences, Prague, Czechia; University of Delhi, INDIA

## Abstract

The ISWI family protein SMARCA5 contains the ATP-binding pocket that coordinates the catalytic Mg^2+^ ion and water molecules for ATP hydrolysis. In this study, we demonstrate that SMARCA5 can also possess an alternative metal-binding ability. First, we isolated SMARCA5 on the cobalt column (IMAC) to near homogeneity. Examination of the interactions of SMARCA5 with metal-chelating supports showed that, apart from Co^2+^, it binds to Cu^2+^, Zn^2+^ and Ni^2+^. The efficiency of the binding to the last-listed metal was influenced by the chelating ligand, resulting in a strong preference for Ni-NTA over the Ni-CM-Asp equivalent. To gain insight in the preferential affinity for the Ni-NTA ligand, QM calculations were performed on model systems and metal-ligand complexes with a limited protein fragment of SMARCA5 containing the double-histidine (dHis) motif. The calculations correlated the observed affinity with the relative stability of the d-block metals to tetradentate ligand coordination over tridentate, as well as their overall octahedral coordination capacity. Likewise, binding free energies derived from model imidazole complexes mirrored the observed Ni-NTA/Ni-CM-Asp preferential affinity. Finally, similar calculations on complexes with a SMARCA5 peptide fragment derived from the AlphaFold structural prediction, captured almost accurately the expected relative stability of the TM complexes, and produced a large energetic separation (~10 kcal∙mol^-1^) between Ni-NTA and Ni-CM-Asp in favour of the former.

## Introduction

Metal ions are very abundant in protein structures, and are frequently key cofactors for protein stability, folding and catalytic functions. Around 30% of all proteins bind minimally one metal ion [1]. The majority (~ 66%) of all ion-binding proteins (*i*.*e*. metalloproteins) contain transition metal ions, about one-third (~37%) contain alkaline earth metal ions (such as Mg^2+^ and Ca^2+^) and ~ 6% contain alkali metal ions [2].

SMARCA5/SNF2H is a divalent magnesium cation—dependent ATPase involved in host–virus interactions via association with the nuclear matrix protein SAFA [3] and chromatin remodeling, as a catalytic subunit of hetero-dimeric complexes, e.g. NoRC [4]. SMARCA5, SWI/SNF-Related, Matrix-Associated, Actin-Dependent Regulator of Chromatin, Subfamily A, Member 5, belongs to the ISWI class of molecular motors that are capable of sliding the nucleosomes along the DNA molecule [5] The nucleosome, which is the elementary unit of chromatin, is composed of ~1.7 helical turns of DNA wrapped around the histone octamer, consisting of 2 copies of each core histones H2A, H2B, H3 and H4 [6, 7]. Nucleosomes represent the first order of packaging of DNA and thus cause obstacles to the binding of the DNA-binding factors. In order to secure coordinated and timely access to the regulatory DNA regions, cells have evolved a large number of chromatin remodeling protein complexes that change nucleosomes position, structure and DNA occupancy. Chromatin remodelers are divided into five families, and each possesses a specific role in these processes. Within the ISWI family, SMARCA5 slides edge- and centre-positioned histone octamers away from their original location on the DNA template [8].

A number of computational methods have been developed to predict the diverse metal-ion binding sites (MIB) and the docking of metal ions [9–11]. The server for protein structure and function prediction I-TASSER [12] generated a model suggesting that the Mg^2+^ ion is coordinated by amino acid residue Thr212 in the catalytic site of SMARCA5 helicase domain (see S8 Table in the **S1 File**). In this case, Mg^2+^ binding requires the presence of associated adenosine diphosphate (S8 Table in the **S1 File**), which is co-incorporated into the protein along with the Mg^2+^ ion. It has been reported that the binding of SMARCA5 in an activated ATP state changes the dynamics of buried histone residues to distort the histone octamer [13].

Importantly, in some ATPases the number of MIBs is not limited to one per molecule. The ATPase from halophilic archaebacterium *Halobacterium (Hbt*.*) saccharouorum* contains a second binding site for divalent metal ions, including the first-row d-block metals Zn^2+^ and Co^2+^. This site is distinct from the metal ion—ATP binding site and its occupation strongly stimulates the rate of ATP hydrolysis and the affinity of the catalytic site for the metal ion-ATP complex [14]. Therefore, it is open to investigation whether SMARCA5 can also bind biologically the important 3d-block metal ion(s) Cu^2+^, Co^2+^, Ni^2+^ and Zn^2+^. Alternatively, these ions (including Zn^2+^) will be referred as transition metals (TMs) further in the text. Four residues have been identified to frequently bind metal ions, Cys, His, Glu and Asp [15] through the atoms of their polar (Cys and His) or charged (Glu and Asp) side chains, which can coordinate metal ions. As reviewed in [16], histidine is the amino acid with the strongest affinity for metal ions, followed by cysteine. In particular, double histidine motifs (dHis) are routinely employed in Electron paramagnetic resonance (EPR) experiments. Spin label complexes of Cu(II) with nitrilotriacetic acid (NTA) display high binding affinity to the dHis motif, with very low dissociation constants [17, 18]. By inspection of the SMARCA5 sequence, a possible candidate site for metal binding was identified in the dHis motif located between ^656^His-Gly-Ala-Thr-^660^His—a site distinct to the Mg^2+^ coordination site—situated near the C-terminal edge of the 16 amino acid-long alpha helix, spanning from ^646^Gly to ^662^Phe (see Fig 3, S11 Fig and S8 Table in the **S1 File**), (I-TASSER) [12].

Practically, metalloproteins can be identified and characterized by various experimental methods: absorbance spectroscopy [19], mass spectrometry [20], nuclear magnetic resonance spectroscopy [21], gel electrophoresis [20], electrophoretic mobility shift assay [22] and immobilized metal ion affinity column chromatography (IMAC) [23]. Immobilized Zn^2+^, Cu^2+^, Ni^2+^ and Co^2+^ ions are routinely employed in IMAC, interacting with amino acid residues on protein surfaces through imidazolyl, thiol and indolyl functional groups. Metal ions have multiple linkages with multidentate chelators covalently attached on resin. After metal—tetradentate chelator complex formation, Zn^2+^, Cu^2+^, Co^2+^ and Ni^2+^ usually have two coordination sites left for protein binding.

Nevertheless, access to many structural and mechanistic details of metalloprotein systems is out of reach by the aforementioned experimental techniques. Theory can provide valuable assistance in elucidating the behaviour of different d-block metals coordinating to protein sites [24]. A solution to these limitations is the application of advanced molecular simulation methods, primarily quantum mechanics (QM) [25–27]. Computational quantum chemistry approaches being used to study metal-containing biomolecules are reviewed in [24, 28] and challenges in determining their binding affinities in [29]. Among these is the density functional theory (DFT) [30, 31], the most preferred method due both to its favourable scaling with the number of atoms and its sufficient degree of accuracy.

**Scheme 1:**
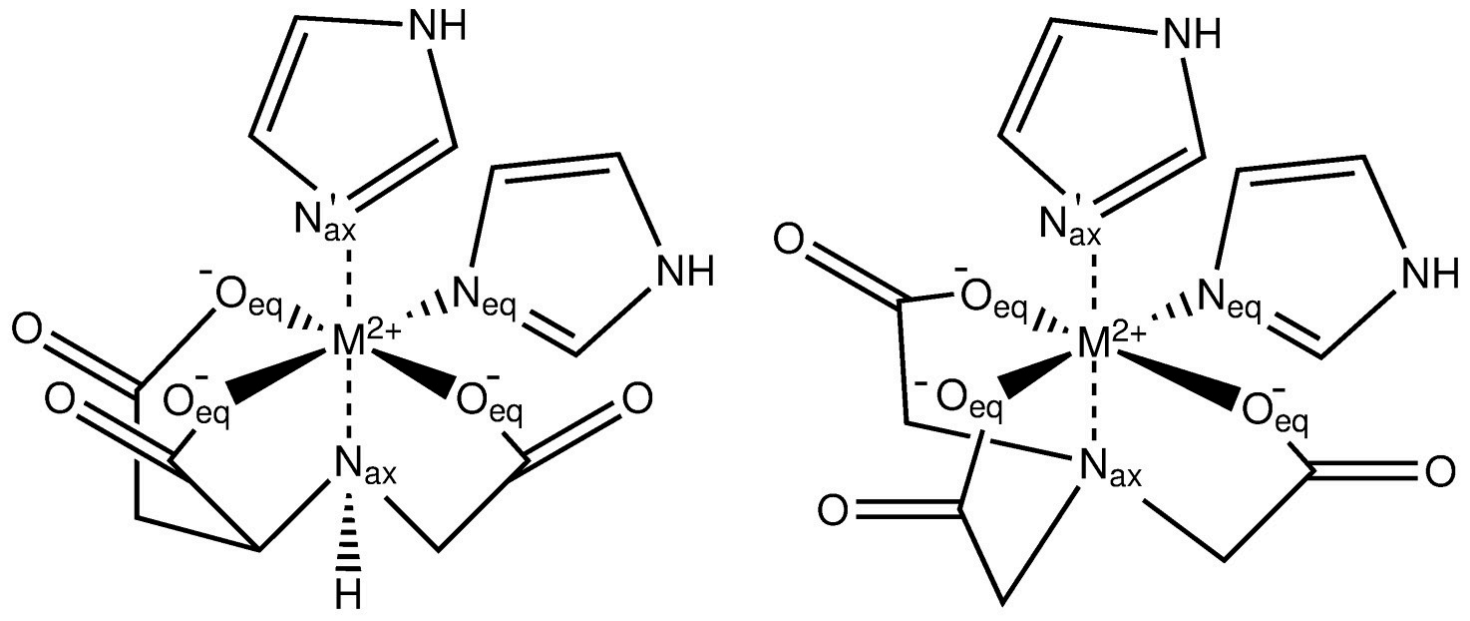
Chemical structure of complexes of a metal dication (M2+) with the chelating ligands CM-Asp (left), and NTA (right). Coordination to two imidazole rings is displayed, and equatorial and axial ligands are labelled. In this work, M^2+^ = Ni^2+^, Co^2+^, Cu^2+^, Zn^2+^.

Here we provide the first evidence of the binding of the SMARCA5 protein to chelated divalent transition metals, immobilized on solid supports. We characterize their binding modes both experimentally, using IMAC, and computationally, using QM calculations. For the computational part of the study, a peptide fragment was constructed from the SMARCA5 protein sequence with the aid of structural prediction methods and was coordinated via the dHis motif to carboxymethylated aspartic acid (CM-Asp), and nitrilotriacetic acid (NTA) supports containing the aforementioned d-block elements. Both are chelating agents capable of forming tetradentate complexes with transition metals, as shown in Scheme 1, illustrating the coordination to two imidazole rings. Differences in coordination between the d-block elements and the protein binding site were analysed and their binding energies were recorded in order to scrutinize the experimental findings.

## Materials and methods

### Expression and purification of recombinant SMARCA5 and TTF1ΔN185

Baculoviruses expressing FLAG-tagged human Snf2H/SMARCA5 [32] and 6xHis-tagged murine TTF1ΔN185 [33] were kindly provided by Dr. Gernot Längst. They were amplified three times in Sf9 cells to produce high titer stocks for protein expression. Sixty hours after infection, cell extracts were prepared by three freeze/thaw cycles followed by three (30-s, amplitude 50%) sonication bursts in a particular lysis buffer. Prior to chromatography, extracts were clarified by centrifugation at 10,000xg (4°C, 30 min).

#### FLAG-SMARCA5

Extraction was carried out in modified EX-500 buffer [33] [500 mM KCl, 10 mM Tris-HCl (pH 7.6), 1.5 mM MgCl_2_, 10% glycerol, 1 mM β-mercaptoethanol, and protease inhibitors] containing 0.2% NP40. The clarified extract was loaded on a TALON Superflow column (GE Healthcare). The resin was washed with EX-500 containing 0.05% NP40 and 5 mM imidazole. The bound protein was eluted with EX-500 containing 250 mM imidazole and dialyzed against the EX-300 buffer (300 mM KCl). The isolation procedure for SMARCA5 is included in the Results section.

#### 6xHis-TTF1ΔN185

The lysis solution K200 [34] consisted of 200 mM KCl, 50 mM phosphate buffer (pH 8.0), 10% glycerol, 0.2% NP40, 1 mM β-Mercaptoethanol and protease inhibitors. The extract was mixed with Heparin-agarose (Sigma) at 4°C for 3 hrs, resin beads washed with K200 and TTF1ΔN185 step eluted with K700 (700 mM KCl). Subsequent chromatography on the TALON column was conducted as described above for FLAG-SMARCA5.

### IMAC charged with various divalent metals

The TALON (*i*.*e*. Co-CM-Asp) and Ni-NTA Superflow (Qiagen) resins were stripped off Co^2+^ and Ni^2+^ ions, respectively, with 0.2 mM EDTA (pH. 7.0). Resins were washed with miliQ water and mixed with 50 mM each of ZnCl_2_, CuSO_4_, NiCl_2_ or CaCl_2_ where appropriate. After washing with 300 mM NaCl and water, cation-charged and uncharged beads were equilibrated in binding and washing (B&W) buffer, *i*.*e*. EX-300 containing 0.5% NP40. For a typical binding reaction, 10 μl beads were pelleted and 1 μg of SMARCA5 or TTF1ΔN185 added in total volume adjusted to 100 μl. The binding mixtures were rotated for 3 hrs at 4°C. The beads were washed four times with 1 ml of B&W buffer and the proteins eluted by boiling in SDS-loading buffer. The proteins were resolved on 8% SDS-PAGE and visualized by Coomassie blue staining. Protein levels were quantified by ImageJ software.

### Step gradient elution assay

SMARCA5 was pre-bound to the Co^2+^-, Zn^2+^- and Cu^2+^—resins and subjected to increasing concentrations of imidazole (indicated in Fig 2A). After a 1-hr incubation at 4°C, beads were pelleted by centrifugation, supernatants collected, and proteins analysed by SDS-PAGE as described above. Elution curves were obtained by four-parameter logistic regression (GraphPad Prism 6.0).

### Computational methods and details

All calculations were performed with the Gaussian program (G16 Rev. C.01) [35]. The hybrid B3LYP [36, 37] and the def2-TZVP [38, 39] combination of DFT functional and basis set was used. For all calculations dispersion corrections were included [40] and since the system under study involved a solvent-exposed portion of SMARCA5, solvation by water was applied via the polarizable continuum model (PCM) [41–43]. For the Cu^2+^ complexes with the SMARCA5 protein fragment, the solvent accessible surface (SAS) was employed on the PCM solvation [44], since the default PCM tesserae resulted in a fragmented solvent surface during the optimization (see S1 Fig in the **S1 File** for a visual comparison of default PCM solvation and PCM with SAS). To ensure comparable energies for the whole series of metal-SMARCA5 complexes (including Cu^2+^), the rest of the structures were also re-optimized with the SAS solvation. This included re-optimization of the metal-ligand complexes and the unbound peptide—required for computing the energies of reactions (2–3), (see Discussion section). The obtained energies were to some extent refined by performing single point calculations with the default PCM on the SAS-optimized structures. This refining applies only to the reported ΔEs which are denoted as SAS//PCM in the **S1 File**. In the main text, all reported structural data in Tables and Figures refer to the default PCM optimizations unless they refer to Cu^2+^/B3LYP. Energies for the model imidazole complexes are derived from PCM calculations and those from the peptide complexes from PCM(SAS) so as to include Cu^2+^ in the comparisons.

A negative total charge of -1 was employed for the majority of the optimizations (except the K^+^ counterion calculations, discussed later in the main text). This afforded a singlet electronic configuration for Zn^2+^ and Ni^2+^ and doublet open shell for the Co^2+^ and Cu^2+^ metal complexes. Optimized structures were verified by vibrational analysis, which also provided entropic and enthalpic contributions to the calculated binding energies. Additionally, counterpoise corrections in the gas phase were performed to counter the basis set superposition error (BSSE) in the computed binding free energies [45, 46]. The typical setup of the two fragments in the counterpoise calculation is illustrated in S9 Fig in the **S1 File**, displaying the two fragments comprising the peptide and the metal-ligand moieties. The BSSE values were halved to balance a possible overestimation [47] and added to the ΔG_b_ and ΔG_b(SAS)_ values of the ligand-metal-SMARCA5 complexes. Moreover, two additional hybrid DFT functionals were employed in the computation of binding free energies, namely TPSSh [48] and MPWB1K [49], the latter reported to perform well on the thermochemistry of transition metals [50]. Unlike B3LYP, optimizations of the Cu^2+^ complexes with these two functionals did not require the usage of SAS in the solvation model. Structural analysis is based primarily on the B3LYP functional throughout the manuscript, while thermochemistry results from the two additional functionals will be examined in the Discussion section together with B3LYP.

## Results

### SMARCA5 binds transient divalent metals with different binding affinity

**Fig 1:**
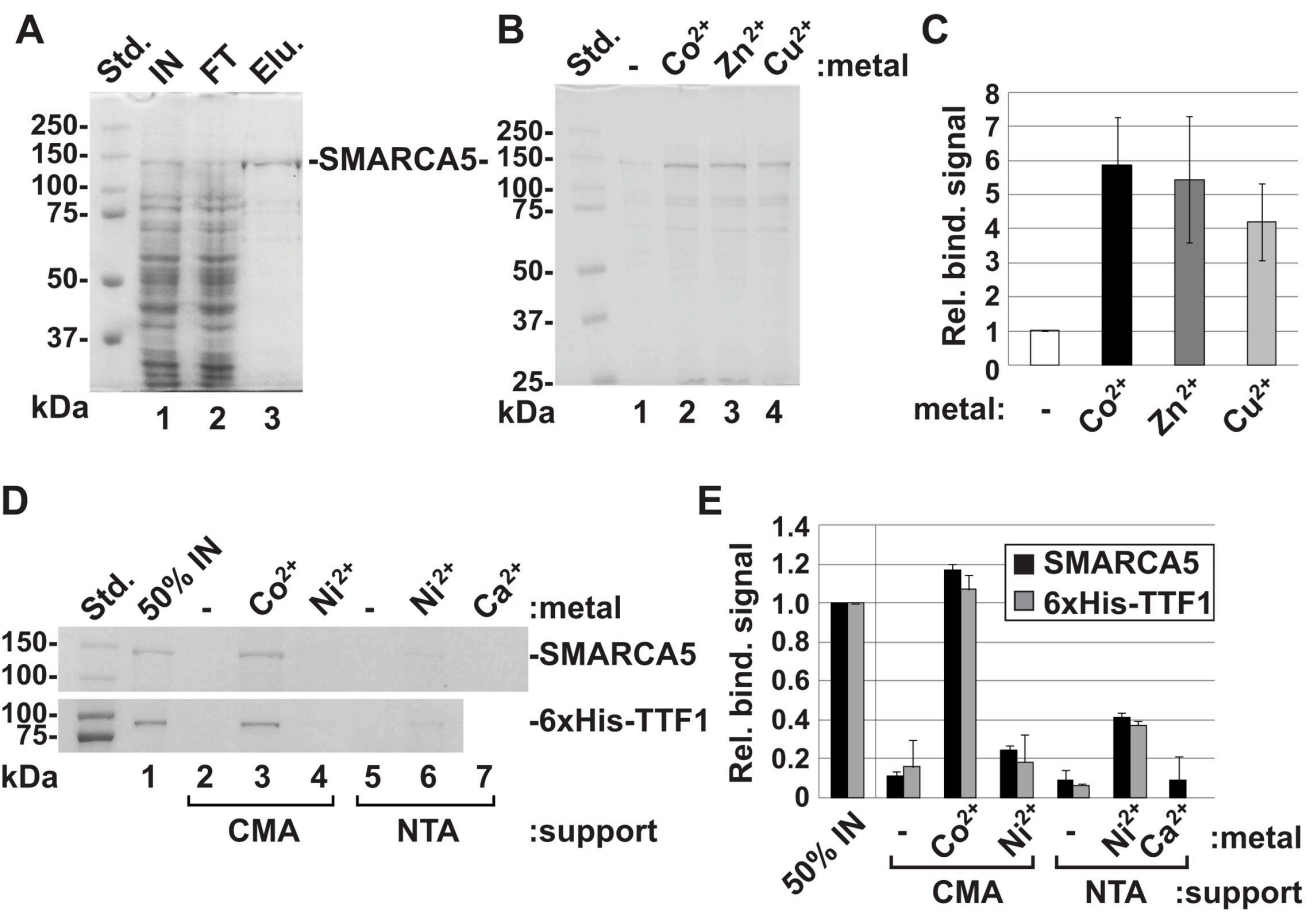
SMARCA5 possesses an intrinsic ability to associate with immobilized divalent transition metals. **(A)** The Sf9 cell extract, containing expressed recombinant FLAG-tagged SMARCA5 (IN), was loaded onto a Co^2+^-charged carboxymethyl-aspartate (CMA) agarose column and flow through collected (FT). The column was extensively washed (see Materials and Methods), and the retained material eluted with imidazole (Elu.). Proteins from each step were analysed by 8% SDS-PAGE. **(B)** The CMA resin, stripped of Co^2+^, was pre-charged with the indicated metals and mixed with the purified SMARCA5. After 3 hrs of incubation the beads were washed and bound SMARCA5 was resolved by SDS-PAGE. **(C)** SMARCA5 protein levels in **B** were quantified and plotted with the level in the no-metal control reaction set to 1. Error bars indicate the SD of values (n = 3). **(D)** Affinity binding set comprised the uncharged CMA-agarose (lane 2) and nitrilotriacetic acid (NTA)-agarose (lane 5), commercially available Co-CMA (lane 3) and Ni-NTA (lane 6) resins, Ni^2+^-pre-charged CMA (lane 4), and Ca^2+^-pre-charged NTA (lane 7) supports. Purified SMARCA5 and 6xHis-TTF1ΔN185 were incubated with each of the resins. After washing, the bound proteins were resolved and visualized. Lane 1 shows 50% of each protein input. **(E)** Protein levels in **D** were quantified and plotted relative to the relevant protein input; 50% value (**D**, lane 1) set to 1. Error bars indicate the range of values (n = 2).

*In silico* metal binding site predictions (see further in the Results section) suggested that SMARCA5 may form complexes with certain transition metal ions. We then explored this question experimentally. To this end, we opted for IMAC [23] with the use of a commercially available TALON resin. TALON is composed of cross-linked agarose with covalently attached tetradentate chelator carboxymethyl aspartate (CM-Asp or CMA), complexed with a Co^2+^ ion. To obtain a starting material for IMAC, we expressed the full-length FLAG-tagged SMARCA5 in Sf9 cells and prepared a whole cell extract (the pattern of resolved proteins by SDS-PAGE is shown in Fig 1A, lane 1). The extract was directly passed through the column, which was pre-equilibrated with 5 mM imidazole to prevent non-specific binding. Note the reduction of the top prominent band (lane 2). The column was thoroughly washed, and the bound material was eluted with 250 mM imidazole (lane 3). Subsequent immunoblotting with a specific antibody against SMARCA5 revealed that the relatively homogenous protein of molecular weight ~140 kDa is, in fact, SMARCA5 (S10 Fig in the S1 File).

To examine whether SMARCA5 can bind to other candidate metal ions, TALON was stripped off Co^2+^ by EDTA and charged with Zn^2+^ or Cu^2+^. Imidazole-free (dialyzed) SMARCA5 was incubated with each pre-charged resin along with the uncharged resin serving as a negative control. Unbound SMARCA5 was washed away, the bound protein was analysed by SDS-PAGE and detected by Coomassie blue staining. The representative gel (Fig 1B) shows that SMARCA5 binds to immobilized Zn^2+^ (lane 3) and Cu^2+^ (lane 4) almost as efficiently as to Co^2+^ (lane 2). Quantitation of binding signals from three independent experiments (Fig 1C) exhibits a shallow gradient of SMARCA5 binding ability that follows the order: Co^2+^ > Zn^2+^ > Cu^2+^.

Apart from TALON, Ni-NTA-agarose is the other widely used affinity chromatography matrix for purifying recombinant proteins carrying a polyhistidine-tag. It consists of a tetradentate chelating ligand nitrilotriacetic acid (NTA) that coordinates a Ni^2+^ ion. In parallel with the commercially available Ni-NTA agarose, we employed a Ni^2+^-charged CM-Asp resin. To ascertain the strength of SMARCA5 binding to immobilized transition metals, we included the 6xHis-tagged N-terminal truncated transcription termination factor 1 (TTF1ΔN185) [33] for comparison. As shown in Fig 1D, both proteins were bound to Co-CMA (lane 3) and Ni-NTA (lane 6) with almost equal strength, although a ~2.5-fold reduction in binding to Ni-NTA was unexpectedly observed (Fig 1E). Regarding the Ni^2+^-charged resins, it is remarkable that binding of both proteins to Ni-CMA is further reduced ~2-fold, thus approaching levels of negative control (compare lanes 4 and 6 in Fig 1D, see quantitation in Fig 1E). Along with the uncharged resins, Ca^2+^-charged NTA-agarose served as an additional negative control for SMARCA5 binding (Fig 1D, lanes 2, 5 and 7, respectively, Fig 1E).

Taken together, we found that SMARCA5 can be efficiently retained by transition metals, Co^2+^, Zn^2+^, Cu^2+^ and Ni^2+^, immobilized onto solid supports. Moreover, its binding affinity slightly exceeds that of the polyhistidine sequence, standardly used for purifying His-tag fusion proteins.

**Fig 2:**
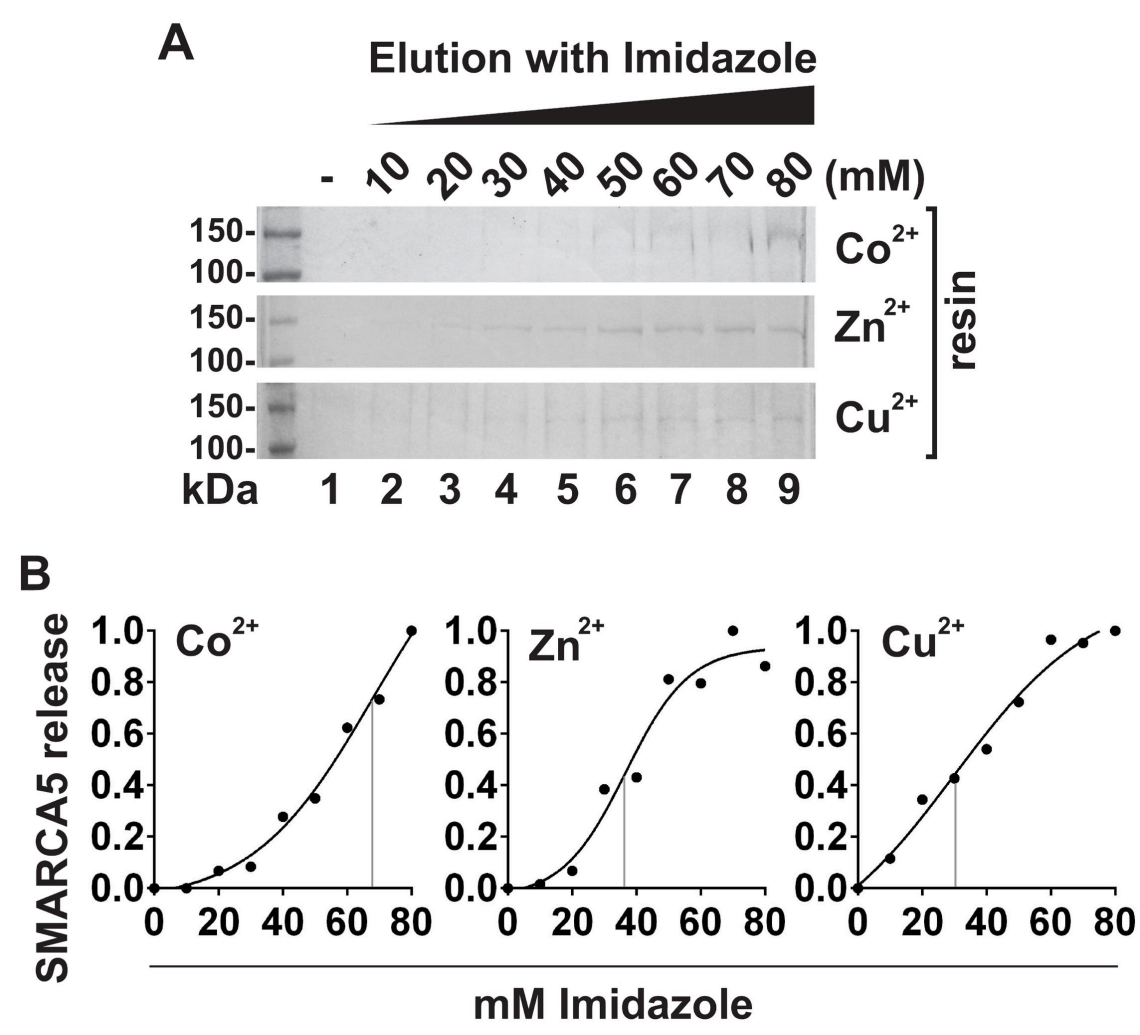
Differential binding affinity of SMARCA5 for Co^2+^, Zn^2+^ and Cu^2+^ metal ions. **(A)** Complexes of SMARCA5 with Co^2+^, Zn^2+^ or Cu^2+^ immobilized on CM-Asp support were subjected to the step-gradient elution with indicated concentrations of imidazole (lanes 2–9). Eluates were collected and analysed by 8% SDS-PAGE. The first lanes represent the mock elution with no imidazole in buffer. **(B)** Protein levels in **A** were quantified and plotted with the highest level of released SMARCA5 set to 1. The grey lines indicate the points of inflection on regression curves. This analysis gave EC50 of 67.9 mM, 36.7 mM and 30 mM imidazole for Co^2+^, Zn^2+^ and Cu^2+^, respectively.

Observed differences in binding affinities for immobilized Co^2+^, Zn^2+^ and Cu^2+^ (Fig 1B and 1C) were further measured by step gradient elution assay. CM-Asp resin-bound SMARCA5 was exposed to the displacer, imidazole in the concentration range from 0 to 80 mM. The released material was collected, analysed by SDS-PAGE (Fig 2A) and quantified. The plotted data were subjected to nonlinear regression analysis, resulting in calculated half maximal effective concentration (EC50) values of 67.9 mM, 36.7 mM and 30 mM of imidazole for Co^2+^, Zn^2+^ and Cu^2+^, respectively (Fig 2B).

### Screen for the metal*-*binding activity of SMARCA5 and structural prediction

Originally, the complete protein sequence of human SMARCA5 was submitted to the Phyre^2^ web portal for protein modelling, prediction and analysis [51]. The modelled 3D structure was analysed by the PyMOL 2.2.0 visualization system. Thorough examination of the structure resulted in a putative double-histidine (dHis) motif for binding Cu^2+^ and Co^2+^ ions that has been published previously [18, 52–54]. It is an *i+4* version where two histidines at positions 656 and 660, separated by three amino acids (*His*-Gly-Ala-Thr-*His*), can potentially form the chelating site. Simultaneous coordination of the metal ion by two histidine side chains is possible only if the dihedral (χ_1_) angles of the residues in positions i and *i+4* are near 180° and -60°, respectively [54]. The initial χ_1_ angles of the histidines 656 and 660 were measured at -170.6° ≡ 189.4° and -69.4°, respectively.

**Fig 3:**
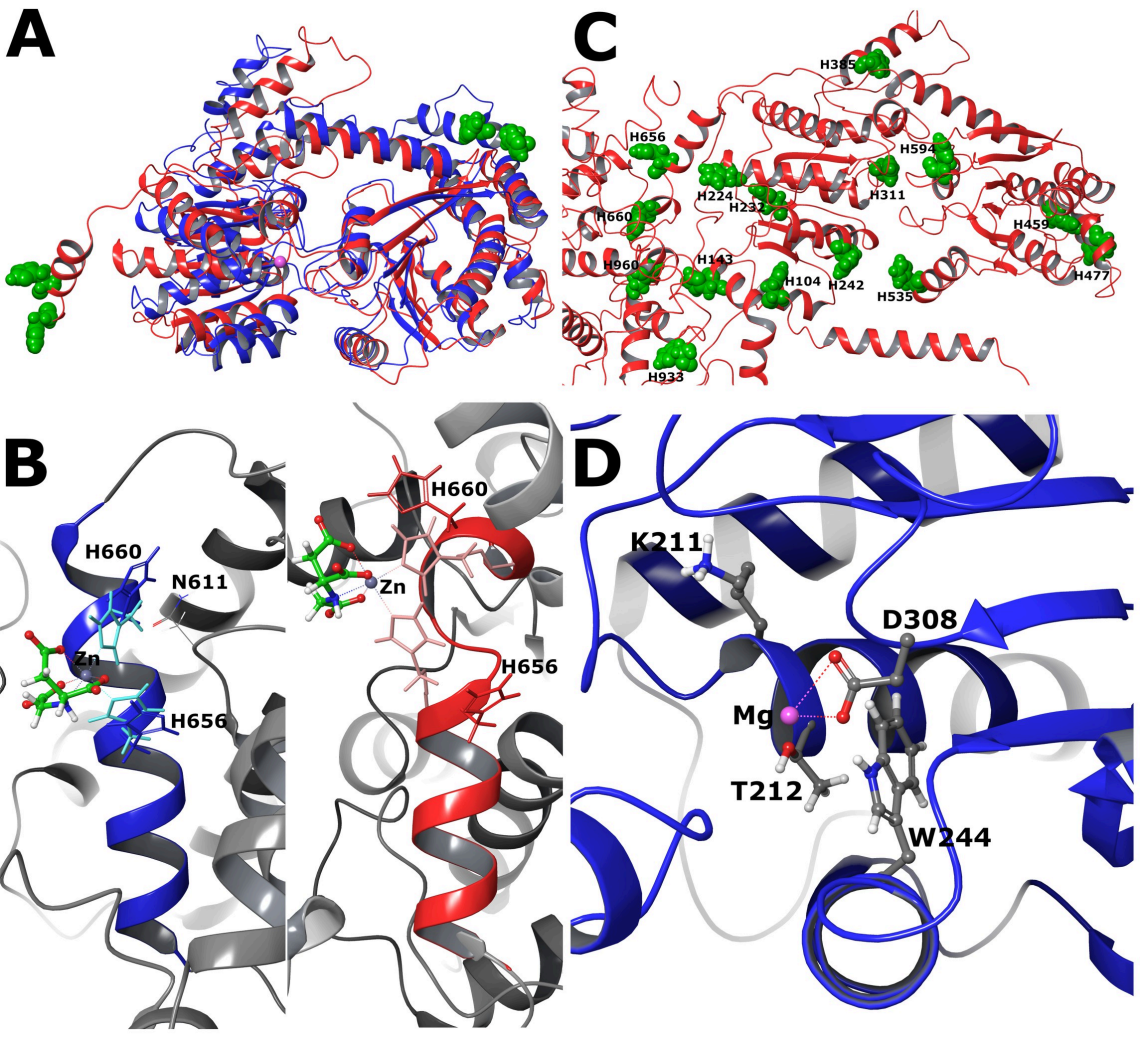
Structural prediction of SMARCA5. **(A)** Alignment of the core region of SMARCA5 (residues 180–632) of the AlphaFold (blue) and I-TASSER (red) structure predictions. ^656^H and ^660^H are shown in green CPK for both structures. The approximate Mg site is indicated by a pink ball. **(B)** Comparison of the dHis motif in the two structures. Histidine side chain positions after coordination with Zn^2+^ are shown in lighter colour. **(C)** Histidine positions on the I-TASSER structure, shown in green CPK. **(D)** AlphaFill prediction of the Mg site overlaid on the AlphaFold structure.

To evaluate further and take advantage of robust methodologies that appeared quite some time after the initial screening, two different structural predictions of SMARCA5 were scrutinized, provided by the I-TASSER and AlphaFold methodologies (Fig 3). Overall, the core region of the protein (residues 180–632) is predicted roughly similar by both methods with an RMSD value of 2.8 Å. The aligned structures of the core region are shown in Fig 3A, with the I-TASSER structure depicted in red and the AlphaFold in blue. Outside that region the structures diverge radically, which is reflected by the very low confidence values of the AlphaFold structure (entry: O60264). Histidines ^656^H and ^660^H lie outside the region of high confidence, yet both methodologies assign them as part of an α-helix encompassing residues 646 to 662 (Fig 3B and S11 Fig in the **S1 File**). Due to this structural divergence, ^611^N is proximal to the dHis motif only in the AlphaFold structure (Fig 3B, left). Fig 3C shows the location of all histidines mapped on the I-TASSER structure with green CPK. Apart from ^656^H-^660^H, two more histidine pairs appear structurally close: ^459^H-^477^H and ^242^H- ^535^H, with C_a_-C_a_ distances of 11.1 Å and 14.3 Å, respectively (11.5 Å is the C_a_-C_a_ distance for the i+4 dHis motif). However, the latter pairs are part of different secondary structures, and their study should be precluded by rigorous Molecular Dynamics simulations, in order to verify their relative positions. Finally, the AlphaFill algorithm [55] and I-TASSER predict a similar location for the Mg active site for both AlphaFold and I-TASSER structures respectively, near residues ^308^D, ^244^W and ^212^T (see Fig 3A and 3D and S8 Table in the **S1 File**). Thus, no synergy can be envisioned between the active site and the dHis motif. The computational study employed both the Phyre^2^ and the AlphaFold structural predictions, which will be alternatively referred further in the text as P2 and AF, respectively. The Phyre^2^ can be considered analogous to the I-TASSER prediction, since both predict an α-helical structure and exclude ^611^N from the vicinity of the binding site.

### Computational results

Unless otherwise mentioned, all results discussed here refer to calculations employing the B3LYP functional. As illustrated in Scheme 1, the ligand oxygens of CM-Asp or NTA coordinate with the metal in the equatorial plane (eq), while the nitrogen occupies the axial position (ax). Then, two imidazole/histidine side chain rings can coordinate to the metal via its two vacant equatorial/axial positions (N_eq_/$N_{\mathrm{ax}}^{'}$) for a completed octahedral geometry.

**Fig 4:**
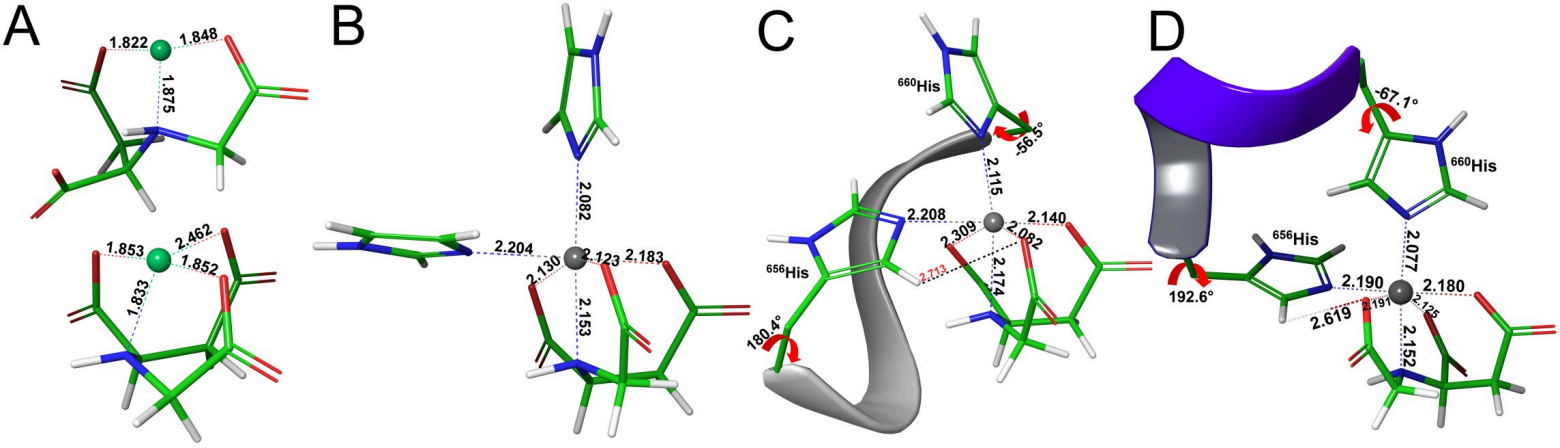
Optimized structures with the functional B3LYP of CM-Asp with: **(A)** Ni^2+^ in tridentate (top) and tetradentate (bottom) coordination, **(B)** Zn^2+^ and two imidazole rings in axial and equatorial positions, **(C)** Zn^2+^ with the with the Phyre^2^-derived protein fragment HGATH represented as a grey ribbon, and **(D)** Zn^2+^ with the with the AlphaFold-derived protein fragment HGATH represented as a violet ribbon. The ^656^His and ^660^His side chains protrude from the ribbons, Ni and Zn atoms are displayed in light green and grey balls respectively, and C, O, N and H atoms in green, red, blue and white sticks, respectively. Key distances are given in Å and χ1 dihedral angles in °.

**Fig 5:**
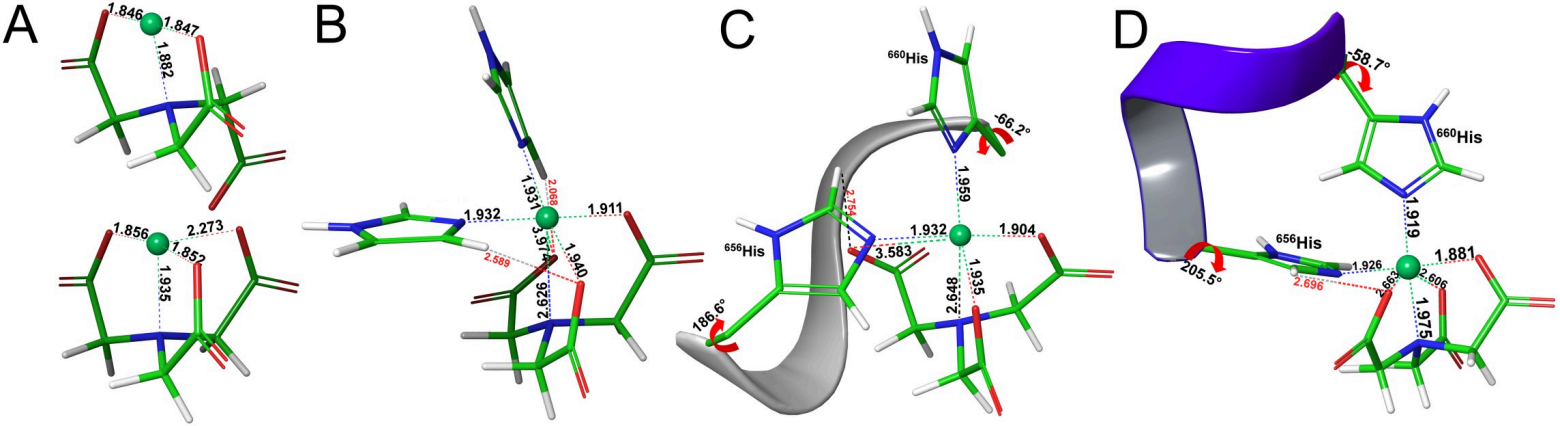
Optimized structures with the functional B3LYP of NTA with: **(A)** Ni^2+^ in tridentate (top) and tetradentate (bottom) coordination, **(B)** Ni^2+^ to form the Ni-NTA_(1)_ complex with two imidazole rings in axial and equatorial positions (for the alternative Ni-NTA_(2)_ complex see S3B, S3C Fig in the S1 File), **(C)** Ni^2+^ to form the Ni-NTA_(1)_ complex with the Phyre^2^-derived protein fragment HGATH represented as a grey ribbon, and **(D)** Ni^2+^ to form the Ni-NTA_(2)_ complex with the AlphaFold-derived protein fragment HGATH represented as a violet ribbon. The ^656^His and ^660^His side chains protrude from the ribbons and Ni atoms are displayed in light green balls, and C, O, N and H atoms in green, red, blue and white sticks, respectively. Key distances are given in Å, and include O_eq_ close distances with the substrate labelled in red, and χ1 dihedral angles in °.

Initially, the preference of the d-block metals to tri- or tetradentate coordination with CM-Asp/NTA was investigated–in the absence of any substrate. Key distances and dihedral angles of the tetradentate complexes, and their relative energies with respect to the tridentate equivalents are reported in Table 1. TMs follow the order: Co-CM-Asp > Zn-CM-Asp > Cu-CM-Asp > Ni-NTA > Ni-CM-Asp in their preference to form a tetradentate complex over tridentate. Ni^2+^ in particular, forms a more stable tridentate complex with CM-Asp by 9.0 kcal·mol^-1^ than the tetradentate equivalent (for their structures, see Fig 4A and S2 Fig in the **S1 File**, top and bottom, respectively) while the ΔΕ value for Cu^2+^ is close to 0 kcal·mol^-1^. Of interest are the averaged equatorial metal-ligand distances (μM-O_eq_), which span 1.96 Å in the doublet metals Co^2+^ and Cu^2+^ and ~2.00 Å in the singlets (Zn^2+^ and Ni^2+^). In contrast, the axial nitrogen distance (M-N_ax_) appears insensitive to the spin configuration. NTA possesses a more flexible ligand scaffold that allows the equatorial oxygens to be more in-plane with the metal. The reported dihedral and metal endocyclic angles for the tetradentate complexes measure the degree of that planarity, the latter displaying values very close to 360° for all complexes, the highest being for Ni-NTA. This is also demonstrated by the θ_OMΟO_ dihedral angles, which is 151° in the Ni-NTA complex, and 112° in the Ni-CM-Asp (Table 1). The tri- and tetradentate structures of Ni-NTA are displayed in Fig 5A, top and bottom, respectively.

**Table 1:** Selected bond lengths (μM-O_eq_, M-N_ax_, in Å) and dihedral and endocyclic metal angles (in °) of the optimized unbound metal-complexes with tetradentate coordination. Relative tridentate-tetradentate energies (ΔE_tri/tetra_) are also included, with positive values (in kcal∙mol^-1^) indicating a preference for a tetradentate coordination and *vice versa*.

	μM-O_eq_	M-N_ax_	θ_ΟMΟO_	Endocyclic	ΔE_tri/tetra_
Zn-CM-Asp	2.010	2.126	141.8	354.9	+4.0
Ni-CM-Asp	2.055	1.884	112.0	357.1	-9.0
Co-CM-Asp	1.965	1.966	139.7	357.2	+4.3
Cu-CM-Asp	1.962	2.035	151.6	358.4	-0.7
Ni-NTA	1.994	1.935	150.9	358.9	-1.0

At this stage, the relative stabilities of the CM-Asp/NTA metal complexes were also tested by adding a counterion K^+^ and recomputing the energies, yielding, largely, identical results with their negatively charged counterparts (values and comparison are included in S1 Table in the **S1 File**). The charge balance alters the order of tetradentate stability only in the estimation of the Co^2+^ tri/tetradentate energy gap (Zn-CM-Asp > Ni-NTA > Co-CM-Asp ≈ Cu-CM-Asp >> Ni-CM-Asp).

Secondly, to investigate the optimal coordinating positions of the imidazole rings without the steric constraints that the protein backbone might impose, model metal-ligand complexes with two imidazole rings at axial and equatorial positions were devised in similar fashion to Scheme 1. Complexes with Ni^2+^, Co^2+^, Cu^2+^ and Zn^2+^ with CM-Asp (Scheme 1, left) were constructed. Pertaining to the complexes with NTA (Scheme 1, right), only Ni^2+^ was considered, in par with the experimental regime (see Figs 4B and 5B for the resulting structures of optimizations on the Zn-CM-Asp and Ni-NTA complexes, respectively, and S3 and S4 Figs in the **S1 File** for the rest of the structures). From the initial octahedral geometry, the Ni^2+^ complexes reverted to trigonal bipyramidal during optimization, irrespective of the ligand, keeping close contact with two of the three equatorial CM-Asp/NTA oxygens (average μM-O_eq_ values of > 2.5 Å, see Table 2 top, 1^st^ column). In the octahedral complexes of Zn^2+^, Co^2+^ and Cu^2+^, μM-O_eq_ values are also elongated (2.1–2.2 Å), and the metal-equatorial plane is more distorted than the corresponding substrate-free tetradentate complexes, with TM endocyclic angles smaller by 15–20°. The imidazole rings maintain a perpendicular orientation with respect to the metal ($N_{\mathrm{ax}}^{'}-\hat{M}$-N_eq_ angles of ~90°, $N_{\mathrm{ax}}^{'}$ denotes the axial imidazole nitrogen as shown in Scheme 1). Binding free energies (ΔG_b_) for the imidazole complexes are also reported (last column, top portion of Table 2), but will be further analysed in the Discussion section. Optimizations of the Ni-NTA complex with the imidazoles yielded two different structures (denoted as Ni-NTA_(1)_ and Ni-NTA_(2)_), with a 4.1 and 3.2 kcal·mol^-1^ difference in energy between them (ΔE and ΔG energies, respectively). The slightly less stable Ni-NTA_(1)_ (Fig 5B), can be thought of as having a rotated axis, where one of the O_eq_ occupies the axial position and the ligand scaffold N atom occupies the equatorial (see S3C Fig in the **S1 File** for this alternate view). A similar type of rotation between axial and equatorial ligands was reported previously for a bis-imidazole Cu-IDA complex [56]. The most stable Ni-NTA_(2)_ retains the CM-Asp coordination motif with the three oxygen atoms in the equatorial and the nitrogen in the axial position, and is shown in S3A Fig in the **S1 File**. Both complexes maintain the perpendicular angle between the Ni and imidazoles (~90°). Additionally, Ni-NTA_(1)_ displays shorter axial distances (μM-N_ax_, $N_{\mathrm{ax}}^{'}$, 1.959 Å). The coordination in both is between tridentate-tetradentate with averaged equatorial distances of ~2.6 Å, lying between the 2.0–2.9 Å values of the fully tridentate and tetradentate Ni-NTA substrate-free complexes. Furthermore, of some importance is the interaction of two of the equatorial oxygens of NTA and CM-Asp with hydrogens of the equatorial imidazole, the average values of which are given under μO_eq_···H_im_ in Table 2. H-bonding ranges in length between 2.3–2.6 Å and is longer in Zn-CM-Asp, while complexes with Ni^2+^ exhibit much shorter values, reflecting their less rigid nature.

**Table 2:** Key distances, angles, dihedral angles and binding free energies of the M-CM-Asp and Ni-NTA complexes with two imidazoles (top section) and the protein fragment (bottom section). Average M-equatorial, M-axial, M-metal and ligand-imidazole/histidine O_eq_ distances are given in Å, angles, endocyclic and dihedral angles are given in ° and binding free energies (ΔG_b_ and ΔG_b,w(SAS)_) are given in kcal∙mol^-1^.

	μM-O_eq_	μM-N_eq_,O_eq_	μM-N_ax_, $N_{\mathrm{ax}}^{'}$	μO_eq_·H_im_	μM	*χ*_1_^656^Η	*χ*_1_^660^Η	θ_ΟMΟO_	$N_{\mathrm{ax}}^{'}-\hat{M}$-N_eq_	Endocyclic	ΔG_b_
**M** ^**2+**^ **[CM-Asp/NTA]** ^**3-**^ **[imidazole]**_**2**_											
Zn-CM-Asp	2.146	2.160	2.117	2.639	2.146	-	-	94.3	92.1	339.3	-6.3
Ni-CM-Asp^a^	2.870	2.637	1.971	2.319	2.309	-	-	113.7	90.1	327.6	-8.1
Co-CM-Asp	2.170	2.124	1.968	2.413	2.072	-	-	97.6	89.4	342.6	-16.6
Cu-CM-Asp	2.260	2.213	2.028	2.442	2.152	-	-	104.9	89.4	339.3	-13.3
Ni-NTA_(1)_^a^	2.608	2.439^c^	2.278^c^	3.600	2.386	-	-	58.4	91.1	267.0	-11.9
Ni-NTA_(2)_^a^	2.575	2.409	1.959	2.436	2.259	-	-	99.6	91.0	331.8	-15.1
**M** ^**2+**^ **[CM-Asp/NTA]** ^**3-**^ **[HGATH] Phyre** ^**2**^ **/I-TASSER**											**ΔG**_**b,w(SAS)**_
Zn-CM-Asp	2.177	2.185	2.145	2.990	2.171	180.4	-56.5	84.7	91.1	330.5	-20.5^de^
Ni-CM-Asp^b^	2.199	2.706	1.921	4.559	2.445	194.5	-56.4	81.3	71.0	350.6	-18.0^de^
Co-CM-Asp	2.101	2.152	1.988	3.082	2.097	181.9	-54.0	71.2	90.7	343.4	-17.2^de^
Cu-CM-Asp^d^	2.080	2.667	2.007	4.456	2.447	196.4	-54.1	132.8	66.0	352.4	-23.7^de^
Ni-NTA_(1)_^a^	2.474^c^	2.339^c^	2.303^c^	2.585	2.327	186.6	-66.2	104.8	91.9	315.7	-20.9^de^
**M** ^**2+**^ **[CM-Asp/NTA]** ^**3-**^ **[HGATH] AlphaFold**											**ΔG**_**b,w(SAS)**_
Zn-CM-Asp	2.165	2.171	2.114	2.924	2.152	192.6	-67.1	93.1	91.1	337.7	-28.6^de^
Ni-CM-Asp^b^	2.671	2.490	1.965	2.873	2.315	196.0	-70.0	113.4	88.7	326.6	-16.0^de^
Co-CM-Asp	2.180	2.130	1.973	2.635	2.078	196.7	-69.7	97.2	88.6	341.4	-30.3^de^
Cu-CM-Asp^d^	2.077	2.454	1.986	3.203	2.298	275.6	-50.1	130.5	119.6	352.9	-30.1^de^
Ni-NTA_(2)_^a^	2.383	2.269	1.947	2.683	2.162	205.5	-58.7	76.6	89.6	322.2	-25.5^de^

^a^ tridentate ligand coordination.

^b^ coordinated only to ^660^H.

^c^ the values reported here disregard the axis rotation.

^d^ computed with PCM(SAS) solvation.

^e^ these ΔG_b_ values are for the H_2_O exchange reaction (3) and are ½∙BSSE corrected.

Lastly, complexes were constructed with the metal-ligands and the amino acid sequence -HGATH- containing the two histidine residues ^656^His and ^660^His, which was extracted from the above-described protein structure predictions of Phyre^2^ (P2) and AlphaFold (AF). The fragment was capped at both ends by CH_3_CO- and -NHCH_3_ and coordinates on the terminal carbons were fixed. Specifically for the AlphaFold structure, residue ^611^N was included in the cluster, and was also capped and fixed at both ends. The computational models were confined to the vicinity of the protein-ligand coordination, which was dictated by previous studies on a similar dHis motif situated on an α-helix of the B1 immunoglobulin-binding domain of protein G (GB1) [57]. Bogetti *et al*, by employing Molecular Dynamics Simulations and monitoring the C_a_-C_a_ distances of the histidine sites—unlabelled or labelled with Cu-NTA—demonstrated that the coordination sites remain sufficiently unperturbed after 200 ns (> 1 Å). This finding was corroborated by their EPR measurements.

Octahedral coordination was imposed to the initial geometries, similarly to the model complexes with the imidazole. From those, Zn^2+^ and Co^2+^ (Fig 4C and 4D and S5A, S5D Fig in the **S1 File**, respectively) maintained the octahedral coordination during the optimization on both P2 and AF structures. Cu^2+^ with both P2 and AF formed trigonal bipyramidal complexes by conserving the tetradentate CM-Asp coordination and bonding only to the axial histidine ^660^H (as mentioned in the computational methods section, the complex with Cu^2+^ was optimized with SAS solvation, see S2 Table and S6B and S7B Figs in the **S1 File**). The Cu^2+^ deviation from the octahedral coordination has been documented previously with B3LYP calculations on its bis-imidazole complex with NTA, where the imidazoles were linked by a carbon chain in an effort to imitate the protein backbone [56]. When unlinked, Cu-NTA reverts to octahedral coordination [57], similarly to the bis-imidazole Cu-CM-Asp analogue shown in S4B Fig in the S1 File. This finding is in contradiction with experimental EPR results, that demonstrate octahedral coordination of Cu-NTA in different dHis sites [58, 59]. Remarkably, we report here that Cu^2+^ coordination is functional dependent since TPSSh concurs with B3LYP while MPWB1K predicts stronger coordination to the equatorial histidine at distances of 2.752 and 2.092 Å, respectively (see S8 Fig and S5 Table in the **S1 File** for a comparison between structures). To clarify whether the Cu^2+^ coordination is sensitive to a restraint between the two coordinating imidazole rings or if this finding is an artifact of the DFT functional, similar EPR experiments on SMARCA5-Cu-CM-Asp (or Cu-NTA) would have to be carried out.

With regards to the complexes of Ni^2+^ with CM-Asp, the P2 and AF structures diverge in the manner of coordination (see S5C, S5F Fig in the **S1 File**). In the P2 structure, Ni^2+^ is coordinated only to ^660^H, while in AF to both histidines. Thus, the former structure possesses a trigonal bipyramidal geometry with a bond to one of the equatorial oxygens more elongated than the others (~2.8 Å), and the latter an octahedral geometry with a similarly elongated Ni-O_eq_ bond.

A measure of the octahedral character of the complexes is provided by their μM values, included in Table 2, which is the average of the length of all six bonds in the TM coordination sphere (*i*.*e*. smaller values signify higher octahedral coordination). Ni-NTA in all its complexes has higher character than Ni-CM-Asp, with the exception of the model imidazole complexes. For the other TMs, they follow Co > Zn > Cu in order of diminishing octahedral character. The above observations reflect the experimental binding affinity, in par with the preference for tetradentate coordination described before.

Optimizations of Ni-NTA with the P2 protein fragment yielded only the equivalent to the model imidazole structure Ni-NTA_(1)_ (Fig 5C). In a similar way to the model structure, Ni^2+^ coordinates to both histidines and in an intermediate tri/tetradentate fashion to the NTA (average oxygen equatorial distances of 2.474 Å, Table 2, middle). On the AF structure, the Ni-NTA_(2)_ conformation was revealed as the most stable (Fig 5D) approaching more towards tetradentate coordination (2.383 Å, Table 2, bottom).

In all the octahedral complexes (*i*.*e*. Ni-NTA with B3LYP/AF, Cu-CM-Asp with MPWB1K, and Zn^2+^, Co^2+^ with all functional/peptide combinations) the equatorial and axial histidine nitrogens lie in a perpendicular angle with respect to the TM, similar to the model imidazole complexes (see $N_{\mathrm{ax}}^{'}-\hat{M}$-N_eq_ values in Table 2). They also exhibit comparable bond lengths to the model complexes, showing that protein interactions have a modest effect on the metal-ligand moiety (Table 2). Zn^2+^ and Co^2+^complexes deviate the least from the imidazole equivalents followed by Ni-NTA_(1)_/P2 and Ni-NTA_(2)_/AF. Inspecting the endocyclic angles about the metal equatorial plane, for the Zn^2+^ and Co^2+^ complexes the deviation from planarity is roughly unperturbed, with angles of 330.5° (337.7°) and 343.4° (341.4°), compared to their analogous imidazole complexes with values of 339.3° and 342.6°, respectively.

In similar fashion to the imidazole complexes, the O_eq_ atoms of the ligands interact with the histidine ^656^H and in addition, with the backbone of the peptide fragment in P2 and the side chains in AF (close contacts are included in S3 Table in the **S1 File** and are specified in S5A, S5D Fig in the S1 File on the Co^2+^ structures). The strongest interactions with the peptide side chains (AF structures, μSc. values) are predicted for Cu, Ni-NTA_(2)_ and Zn in order of diminishing strength. For peptide backbone interactions (P2 structures, Bb. values), the strongest interactions are predicted for Ni-NTA_(1)_, followed by Zn and Cu, again in order of diminishing H-bonding strength. The computed ΔG_b_ binding free energies of the above-mentioned complexes are included in the last column of Table 2 and will also be scrutinized in the following section. For a more extensive discussion of the TPSSh and MPWB1K results, see Section 4 in the **S1 File**.

## Discussion

It is evident from the experimental section (Fig 1C and 1E), that the TMs follow the sequence: Co-CM-Asp > Zn-CM-Asp > Cu-CM-Asp > Ni-NTA > Ni-CM-Asp, in order of decreasing SMARCA5 binding affinity. One of the computed energetic properties that precisely matches the above experimental order is the preference for tetradentate coordination over tridentate (ΔE_tri/tetra_, Table 1). As mentioned in the Results Section, this is also reflected in the octahedral coordination ability of the TMs in the optimized complexes as recorded in Table 2 (μM values). To evaluate the experimental affinity order further, binding free energies were computed for all imidazole and peptide complexes. These follow the elementary reactions:

$$2\cdot \mathrm{Imidazoles}+M^{2+}-\mathrm{Ligan}d^{3-}\to \mathrm{Imidazol}e_{2}-M^{2+}-\mathrm{Ligan}d^{3-}$$ (1)

$$\mathrm{Peptide}+M^{2+}-\mathrm{Ligan}d^{3-}\to \mathrm{Peptide}-M^{2+}-\mathrm{Ligan}d^{3-}$$ (2)

Apart from the ΔE and ΔG_b_ energies computed for the above reactions, counterpoise calculations were performed for reaction (2). Those were executed either as single point calculations on the PCM-optimized geometries (PCM-SAS for Cu^2+^) or as full-gas phase counterpoise optimizations. The former estimated the BSSE errors for each complex, which were halved [29, 47] and inserted in the ΔG_b_ energies reported in the last column of Table 2. It has to be noted however, that the inclusion of BSSE corrections did not alter the TM order of binding strengths in any case. The counterpoise calculations estimate quite large binding energies and are used only for relative comparisons between TMs (S7 Table in the **S1 File**). To faithfully match the product of reaction (2) in the estimation of ΔGs, the energy of the tetradentate M-Ligand was inserted in the left part of the reaction for Co^2+^ Cu^2+^ and Zn^2+^. Accordingly, in the case of the Ni^2+^ complexes with CM-Asp/NTA, the energies of the tridentate Ni^2+^-Ligand were employed.

As mentioned in the Computational methods and details section, it was not possible to optimize the Cu-CM-Asp complex with the peptide using the default solvation when using B3LYP. In order to include Cu^2+^ in the comparison, relative ΔG_b(SAS)_ energies were computed, and were corrected with ½∙BSSE (see corresponding ΔG_b(SAS)+1/2∙BSSE_ fields in S6 and S7 Tables in the **S1 File**). They were found in the order: Ni-NTA_(1)_ > Cu = Ni > Co > Zn, for the P2 structures, and Co > Ni-NTA_(2)_ > Cu > Zn > Ni for the AF structures, with decreasing complex stability. Both obtained series overestimate Ni-NTA and underestimate Zn^2+^ binding, with respect to the experimental binding affinity. On the other hand, the prediction that Ni-NTA binds relatively stronger that the CM-Asp equivalent is prevalent in most computed energies by the B3LYP functional, which is only reversed by TPSSh and MPWB1K on the P2 structures (see S6 and S7 Tables and the relevant discussion in the **S1 File**).

For the B3LYP-optimized P2 structures, the Ni^2+^, Co^2+^ and Cu^2+^ ΔG_b(SAS)_ values lie within a ~2 kcal∙mol^-1^ range, with the Zn^2+^ complex set apart and comparatively disfavoured. This is also apparent in the P2 ΔG_b_ values with the other two functionals. For the AF structures with B3LYP, the strongest binding TMs span 8 kcal∙mol^-1^, apart from the weakest Ni^2+^. Thus, the computed energies are more evenly spaced, which also holds true for the MPWB1K computed energies.

In contrast to the calculated series of reaction (2), the values that manage to resemble the experimental affinities the most are the ΔG_b_ values computed for reaction (1) of the imidazole model complexes (top of Table 2, rightmost column). These follow the order: Co-CM-Asp > Cu-CM-Asp > Ni-NTA_(1)_ > Ni-CM-Asp > Zn-CM-Asp. In this case, only Zn^2+^ is at odds with the experimental affinity order.

Some validity to the binding free energies computed by Eq (2) is provided by experimental data in the literature. A ΔG_max_ value of -8.9 kcal·mol^-1^ has been reported previously for a monoclonal antibody (Mab) absorbed on PEVA-CM-Asp-Zn(II) (PEVA: poly(ethylene vinyl alcohol)) [60]. This lies closer to the computed energy for the Zn-CM-Asp complex with imidazole (ΔG_b_ = -6.3 kcal·mol^-1^, Table 2) rather than the peptide (ΔG_b_ = -14.5 kcal·mol^-1^ for P2 and -17.7 kcal·mol^-1^ for AF, S6 Table in the **S1 File**). The disparity can be attributed to the different topology of the antibody when compared to SMARCA5. Nevertheless, this raises the question about a local distortion in the vicinity of the binding site that might not be captured with the computational setup based on the current structural predictions. With regards to the histidine χ1 dihedral angles, as mentioned before, these are expected at around 180° and -60°, for ^656^H and ^660^H, respectively [54]. For the optimized structures, these are included in Table 2 and show that for each TM, the P2 angles are closer to the optimal than the equivalent AF, but the latter do not deviate more than ~10-15°. Evidently, the ^656^H χ1 dihedrals of the non-coordinating complexes such as those of Ni^2+^ and Cu^2+^ exhibit a much larger deviation. From the above it can be surmised that, in the vicinity of the binding site—the computed structures can be relied upon, even though the conformational space of the protein-ligand interface was not extensively sampled.

The ΔGs can be also calculated for a modified reaction (2) which accounts for the exchange of coordination to the metal between water and the peptide:

$$\mathrm{Peptide}+{\left[H_{2}O\right]}_{2}M^{2+}-\mathrm{CM}-\mathrm{As}p^{3-}\to \mathrm{Peptie}-M^{2+}-\mathrm{CM}-\mathrm{As}p^{3-}+2\cdot H_{2}O$$ (3)

In the complexes with water, only one of the two H_2_O molecules is coordinated to the TM with the exception of Zn^2+^ and Co^2+^ (see S4 Table in the **S1 File** for the TM···OH_2_ distances), a finding that has been reported previously for Cu-NTA, with a Cu···OH_2_ distance larger than 4.0 Å [57].

Calculation of the free binding energies for reaction (3) yields the following series in order of decreasing binding strength, for the P2 structures: Cu > Ni-NTA_(1)_ ≈ Zn > Ni ≈ Co, while for AF, the relative binding strengths are provided by the series: Co ≈ Cu > Zn > Ni-NTA_(2)_ >> Ni. Thus, the AlphaFold-based results are the closest of any set of calculations to the experimental affinities. Only the Zn^2+^ binding strength is underestimated at -28.6 kcal·mol^-1^ when compared to Cu^2+^ at -30.1 kcal·mol^-1^ (Table 2, ΔG_b,w(SAS)+1/2∙BSSE_ values).

Interestingly, the computed B3LYP energies for the P2-based structures follow approximately the Irving-Williams series of relative stability: Co < Ni < Cu > Zn [61], (ΔG_b,w(SAS)_ values in Table 2, excluding Ni-NTA), which differ from the observed affinities. Protein-metal systems that go counter to the established series have been observed before [62–64], showing that further investigation is warranted into the SMARCA5-TM preferential affinity. In this respect, it is of note that an intrinsic metal specificity of class Ib RNR was found *in vitro*, which in turn might lead to the selection of biologically relevant cofactor *in vivo* [56]. However, as mentioned above, the calculations on the AF structures with B3LYP do not corroborate this finding, predicting a counter-Irving-Williams relative stability which approximates the experimentally observed one. Calculation of the energies of reaction (3) with the other functionals, reveals Cu^2+^ as the most stable complex, as per the Irving-Williams series, but Co^2+^ binding is overestimated (S7 Table in the **S1 File**, ΔG_b,w_ rows).

Inspecting the differences in coordination across all TMs, as was described for the complexes with imidazole (S5 Table in the **S1 File** shows a complete overview with coordination assignments of all calculated species), Cu^2+^, Co^2+^ and Zn^2+^ coordinate to both imidazole nitrogen atoms and the three equatorial oxygens of CM-Asp, while Ni^2+^ binds both imidazoles but only two of the equatorial oxygens. In the protein fragment complexes, Co^2+^ and Zn^2+^ exhibit the same coordination pattern. Cu^2+^ prefers to bind only to ^660^H, though optimization with the MPWB1K functional results to octahedral coordination, especially for the AF-based structure. For Ni-CM-Asp, P2-based structures favour coordination only to ^660^H, while AF-based favour two histidine coordination, both in trigonal bipyramidal fashion and regardless of the functional.

On the comparison between the Ni^2+^ complexes with CM-Asp and NTA, the difference in the computed ΔG_b,w_ energies is over 10 kcal∙mol^-1^ in favour of the latter (AF-based structures), and 7 kcal∙mol^-1^ in favour of Ni-NTA_(2)_ in the model imidazole complexes. Apart from the energetics, other characteristics of the complexes can shed a light into their divergent behaviour. For Ni-CM-Asp, the inherent tridentate stability when unbound was demonstrated, in contrast to Ni-NTA which opts for tetradentate ligation. In their model complexes with the imidazoles both exhibit a similar coordination pattern, with Ni-NTA_(2)_ displaying the strongest octahedral character. In their P2-based complexes with the protein fragment, only Ni-NTA is capable of binding to both histidines of the dHis motif. If we take μM as an indication of their binding ability, Ni-NTA is the complex with strongest binding after Co^2+^ and Zn^2+^ (Table 2 and S5 Table in the **S1 File**). Additionally, Ni-NTA exhibits stronger interaction with the protein environment; this is attested by the shorter distances of its H-bonding network in comparison to Ni-CM-Asp (see μBb./SC. and Avg.All values in S5 Table in the **S1 File**).

Overall, the calculations support the experimental findings that Ni-NTA has the ability to bind to the dHis motif of SMARCA5, with higher affinity than Ni-CM-Asp. While the computed structures limit the scope to the binding site of the protein-metal-ligand system, they are of adequate quality, manifested by their compliance with experimental energetic and structural data. Finally, the experimental affinity order of CM-Asp/NTA bound to d-block metals is captured by: (1) the computed preference for tri- or tetradentate ligation in the free metal-ligand system, (2) the TM capability for octahedral coordination, (3) partially by the relative binding energies of TMs on model systems with imidazole, and (4) by the relative binding energies of TMs on the AlphaFold-based structures. The prediction of the binding energies could benefit from a dynamic treatment of an extended ligand-metal-SMARCA5 cluster, rather than the limited size static treatment that was presented here. Moreover, the participation on the binding of alternate double-Histidine sites, such as the ones suggested by the structural analysis in the relevant chapter, cannot be ruled out, but the dHis motif analysed here should be the primary, most stable site. To settle this ambiguity, a mutagenesis study would have to be carried out on all three possible sites of SMARCA5.

Regarding possible structural or functional interplay between the catalytic Mg^2+^ ion and d-block metals investigated herein. Firstly, their binding sites are located at distant parts of SMARCA5 molecule, helicase ATP binding domain and spacer between helicase C-terminal and HAND domain, respectively (see Fig 3 and S8 Table in the **S1 File**). On the contrary, Shobert [14] claims that the second MBS and catalytic site are likely to be located close to each other in halobacterial ATPase because they appear to functionally interact and thus participation of the metal ion in catalysis is conceivable. Secondly, the ATP-binding site of SMARCA5 contains the common Walker A and Walker B motifs, which are characteristic for many ATPases, including the Superfamily II (SF2) DNA helicases [65]. Consensus sequence of Walker B motif, hhhhDE (h represents a hydrophobic amino acid) is involved in ATP binding and hydrolysis [66]. The conserved aspartate and glutamate residues (DE) facilitate coordination of the Mg^2+^ and water molecules for ATP hydrolysis [65, 67]. Mutation of residue Glu309 in the Walker B motif of SMARCA5 inhibits ATP hydrolysis [68]. Given the structural and spatial differences between dHis and hhhhDE motifs it seems rather unlikely that d-block metals could substitute for Mg^2+^ in binding pocket.

Attempting to interpret the significance of our experimental findings and computational results on the SMARCA5 association with certain d-block metals, we discuss here the major role of SMARCA5 in the context of cell. Many biological processes are frequently regulated by a particular chromatin state, where the access of sequence-specific DNA-binding proteins to the regulatory DNA regions (promoters, enhancers, silencers and insulators) is critical for the activity of relevant genes. Chromatin remodelers are involved in multiple DNA-dependent processes, namely in regulation of transcription, replication, DNA repair, chromatin assembly and recombination. These remodelers are energy-consuming DNA translocases that are able to manipulate and restructure nucleosomes in four ways: 1) sliding along DNA, which exposes previously inaccessible sequences and occludes regulatory sequences in a new histone octamer position, 2) ejection or complete displacement of the octamer, 3) taking away of the H2A-H2B dimer (leaving the H3-H4 tetramer only), and 4) histone H2A-H2B dimer exchange (reviewed in [69]). It is of note that both sliding and ejection necessitate the loss of all 14 contacts between histones and DNA, which requires a total energy of about 12–14 kcal mol^-1^ [70].

It has to be noted that we characterized interactions between SMARCA5 and chelated divalent transition metal ions on supports. Possible action of free (nonchelated) TMs in relevant assays: ATPase assay, nucleosome mobility assay, etc., were beyond the scope of this study. It is still therefore on the level of speculation that, apart from an alkaline earth metal Mg^2+^ ion, SMARCA5 might require also divalent TM ions for a maximal translocase enzyme activity. Alternatively, and independently of the catalytic Mg^2+^ ion, these metals might be involved in SMARCA5 protein conformational changes, which is turn stabilize its interaction with accessory subunits within the chromatin remodeling complexes or with proteins facilitating antiviral immunity. Future experimental and computational studies looking at the structures and activity of complexes, consisting of SMARCA5, its associated subunits and d-block metals, will help advance our understanding of how ISWI family motors reach their full potential.

## Supporting information

S1 File Additional figures, tables and cartesian coordinates for all calculated species, verification of SMARCA5 identity and prediction of the Mg^2+^ binding site. (PDF)

S1 Raw images (ZIP)

S1 Data (BZ2)
