# Supplementary material for: The chromatin remodeler SMARCA5 binds to d-block metal supports: Characterization of affinities by IMAC chromatography and QM analysis
Source: PLoS One. 2024 Oct 7;19(10):e0309134. doi: 10.1371/journal.pone.0309134 (PMC11458017; doi:10.1371/journal.pone.0309134)
Supplement: S1 File — (PDF) [file pone.0309134.s001.pdf]

ELECTRONIC SUPPLEMENTARY INFORMATION

The chromatin remodeler SMARCA5 binds to d-block metal supports: Characterization of affinities by IMAC chromatography and QM analysis

Prokopis C. Andrikopoulos,*[§a,b] Pavel Čabart*[§c]

[a] Institute of Biotechnology of the Czech Academy of Sciences, BIOCEV, Průmyslová 595, CZ-252 50 Vestec, Czechia
[b] Charles University, 1st Faculty of Medicine BIOCEV, Průmyslová 595, CZ-252 50 Vestec, Czechia
[c] Institute of Experimental Medicine of the Czech Academy of Sciences, Vídeňská 1083, CZ-142 20 Prague 4, Czechia

* Correspondence to: Prokopis C. Andrikopoulos; e-mail: prokopios.andrikopoulos@lf1.cuni.cz; and Pavel Čabart e-mail: pavel.cabart@iem.cas.cz

[§] P. C. Andrikopoulos and P. Čabart contributed equally to this work and both should be considered as first authors.

CONTENTS

Page 2 1. Solvent Continuum methods
Page 2 Fig.S1 CM-Asp-Co-SMARCA5 with default PCM and SAS solvation sphere.

Page 3 2. Counterion Calculations
Page 3 Table S1 The effect of the counterion (K^+) on the relative tri/tetradentate stabilities of the M-CM-Asp/NTA complexes.

Page 4 3. Figures and Tables of Computed Structures
Page 4 Fig.S2 Metal-CM-Asp structures of Co^{2+}, Cu^{2+}, and Zn^{2+}.
Page 4 Fig.S3 Ni-NTA-imidazole structures 1 and 2.
Page 5 Fig.S4 Metal-CM-Asp-imidazole complexes of Co^{2+}, Cu^{2+}, and Zn^{2+}.
Page 5 Fig.S5 Metal-CM-Asp-peptide complexes of Co^{2+}, Cu^{2+}, and Ni^{2+}.
Page 6 Fig.S6 PCM-SAS Metal-CM-Asp/NTA-peptide complexes of Co^{2+}, Cu^{2+}, Ni^{2+} and Zn^{2+} based on the Phyre[2] structural predictions.
Page 7 Fig.S7 PCM-SAS Metal-CM-Asp/NTA-peptide complexes of Co^{2+}, Cu^{2+}, Ni^{2+} and Zn^{2+} based on the AlphaFold structural predictions.
Page 7 Fig.S8 Metal-CM-Asp peptide complexes of Cu^{2+}, optimised with the MPWB1K and TPSSh DFT functionals.
Page 8 Table S2 Average distances, angles and dihedrals of the PCM-SAS-optimized complexes based on the Phyre[2] and AlphaFold structural predictions.
Page 8 Table S3 Ligand-peptide close contacts of B3LYP optimized complexes based on the Phyre[2] and AlphaFold structural predictions.
Page 8 Table S4 Metal-water M···OH_2 distances in the Metal-CM-Asp complexes.
Page 9 Table S5 Analytical structural information for all optimized complexes.

Page 10 4. Correlating SMARCA5 Computed Binding Energies with Experimental Binding Affinities
Page 10 Fig.S9 Fragment setup for counterpoise calculations.
Page 11 Table S6 Different computed binding energies for the Metal-SMARCA5 complexes.
Page 12 Table S7 Metal series arranged in order of increasing complex binding stability.

Page 13 5. Verification of SMARCA5's Identity after Isolation
Page 13 Fig.S10 The immunoblot analysis of SMARCA5 with the specific antibody.

Page 14 6. Predictions of Mg^{2+} - Binding Site and Double Histidine Structural Motif
Page 14 Table S8 I-TASSER prediction of ligand-binding sites in SMARCA5.
Page 14 Fig.S11 I-TASSER prediction of secondary structure of the dHis site.

Page 15 7. Coordinates of all optimized species with the B3LYP functional

1. Solvent Continuum methods

All metal-ligand and metal-ligand-imidazole complexes were computed effortlessly with PCM, while in their complexes with SMARCA5 and the functional B3LYP, Cu^{2+} required the application of the solvent accessible surface (SAS) to the PCM solvation. Therefore, Ni^{2+}, Co^{2+} and Zn^{2+} complexes were also re-computed with SAS to yield comparable – albeit less accurate – energies for the whole Co^{2+} – Ni^{2+} – Cu^{2+} – Zn^{2+} series of complexes with SMARCA5. In **Fig.S1** optimized structures of the Co-SMARCA5 complex are displayed including the solvent cavities using the default PCM and the PCM-SAS spheres.

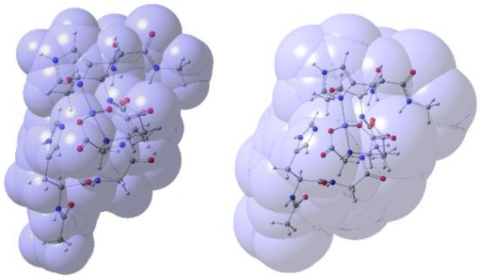

Fig.S1 Structures that illustrate the PCM cavities of the B3LYP-optimized CM-Asp-Co-SMARCA5 complex (Phyre[2] structure) with the default PCM solvation (left) and after the application of the Solvent Accessible Surface (SAS, right).

2. Counterion Calculations

Table S1 The effect of the counterion (K^+) on the relative tri- and tetradentate stabilities of the M-CM-Asp/NTA complexes is shown on the left column. For comparison, the right column contains the relative energies without a counterion, repeated from **Table 1**. Positive values indicate a more stable tetradentate ligand and *vice versa*. All values are given in kcal·mol^{-1} and are taken from the B3LYP-optimized species.

**Complex**	**M^{2+}[CM-Asp]$^{-3}$$K^+$ ΔE_{PCM}**	**M^{2+}[CM-Asp]$^{-3}$ ΔE_{PCM}**
Zn-CM-Asp	+3.73	+3.99
Ni-CM-Asp	-8.57	-9.01
Co-CM-Asp	+0.21	+4.35
Cu-CM-Asp	-0.10	-0.69
Ni-NTA	+1.45	-1.04

3. Figures of Optimized Structures

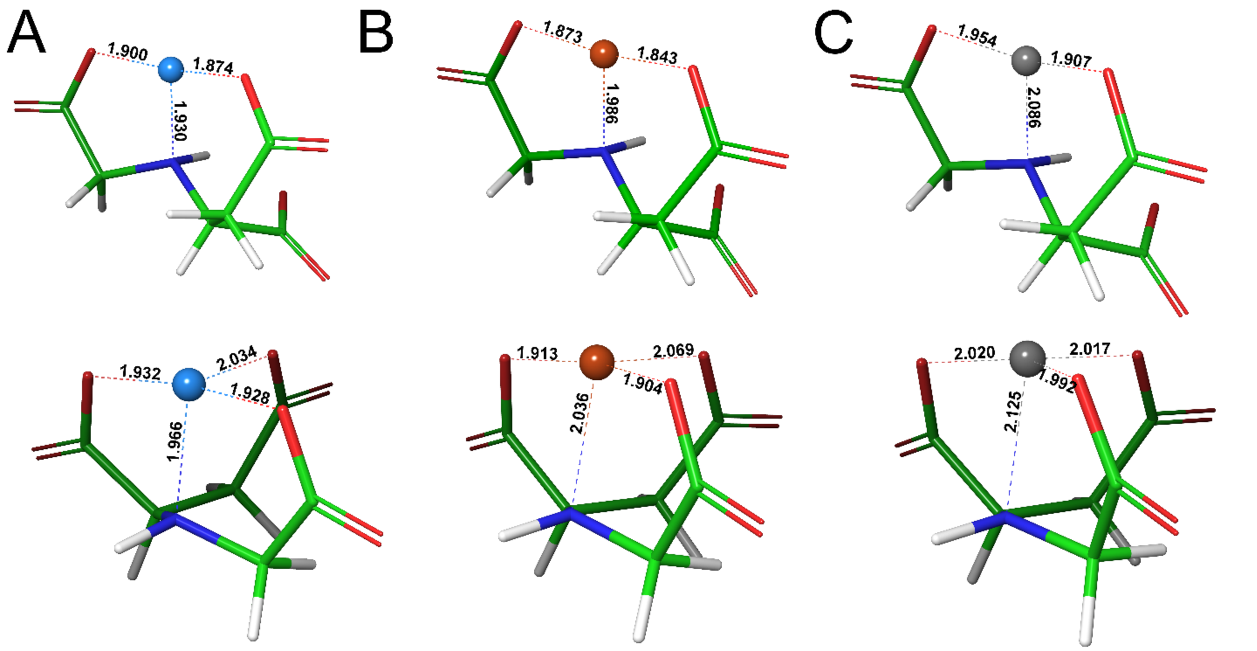

Fig.S2 Optimized structures with B3LYP of Metal-CM-Asp complexes of (A) Co^{2+}, (B) Cu^{2+}, and (C) Zn^{2+}. The tridentate optimized structures are displayed on the top row and the tetradentate on the bottom. Co atoms are shown in light blue and Cu in orange and Zn in grey balls. C atoms are shown in green, O in red, N in blue and H in white sticks. Key distances are given in Å.

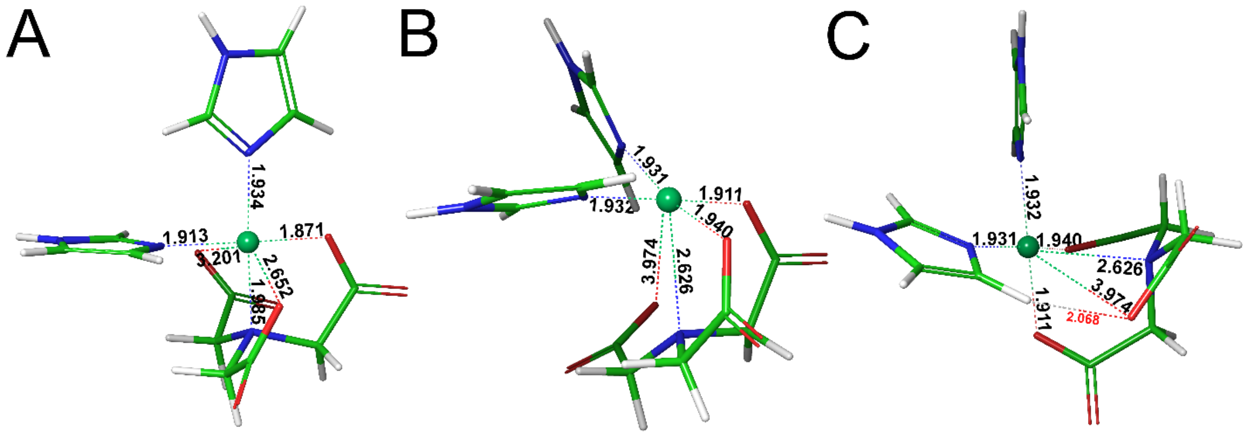

Fig.S3 Optimized structures with B3LYP of: (A) the Ni-NTA$_{(2)}$ complex with imidazoles, (B) and (C) the Ni-NTA$_{(1)}$ complex with imidazoles (repeated from **Fig.5B**) in two views, the latter with a ligand oxygen occupying the axial position. Key distances are given in Å. In (C), a close contact between an equatorial NTA oxygen and one of the imidazole rings is labelled in red.

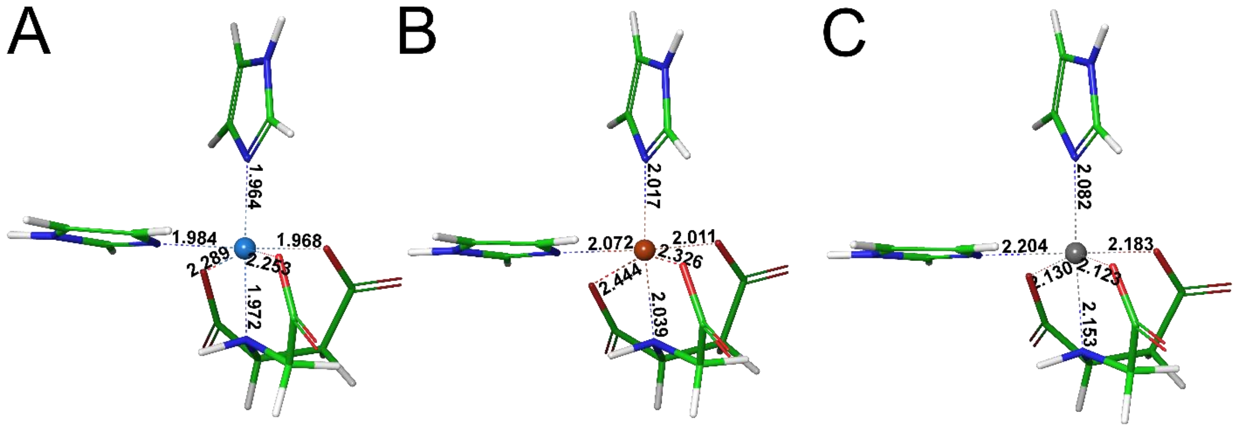

Fig.S4 Optimized structures with B3LYP of imidazole Metal-CM-Asp complexes with: (A) Co^{2+}, (B) Cu^{2+}, and (C) Zn^{2+}. Co atoms are shown in light blue, Cu in orange and Zn in grey balls. C atoms are show in in green, O in red, N in blue and H in white sticks. Key distances are given in Å.

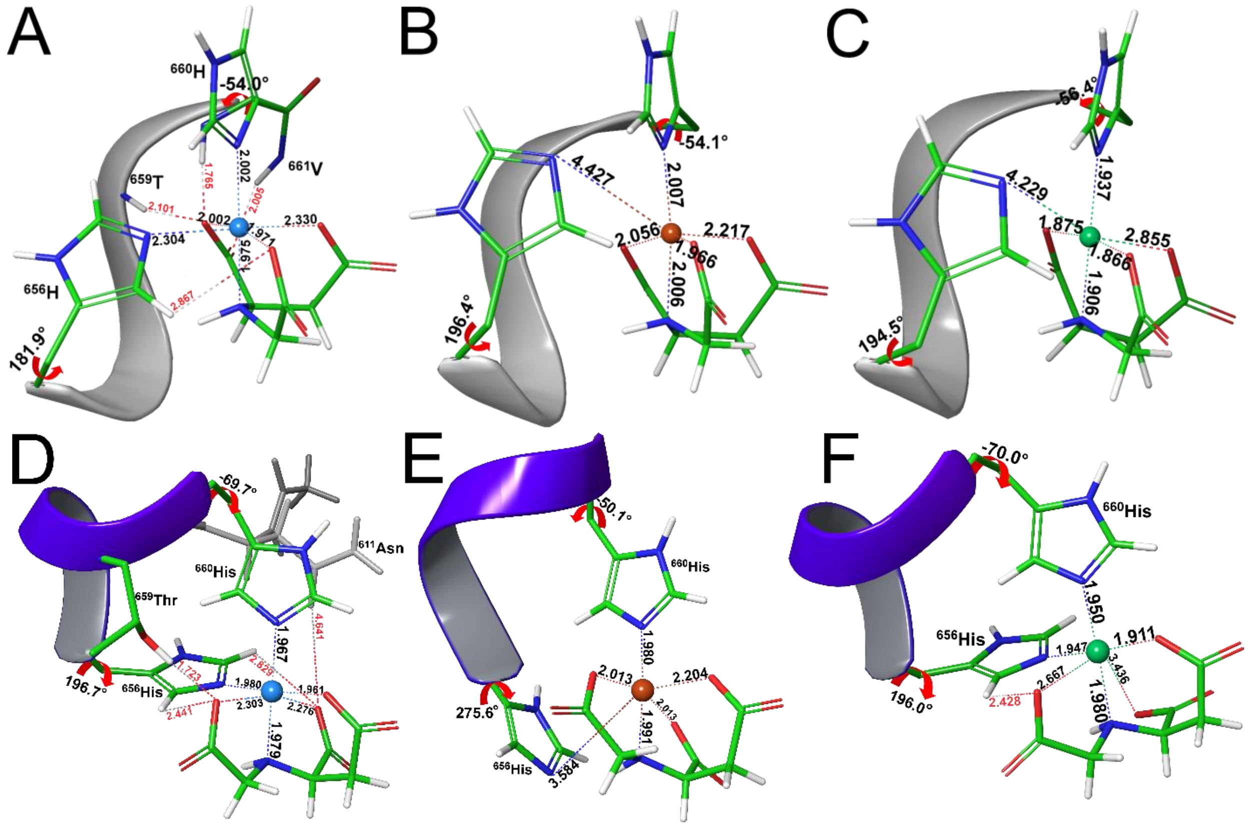

Fig.S5 Optimized structures of the peptide Metal-CM-Asp complexes with B3LYP of (A) Co^{2+}, (B) Cu^{2+}(SAS), and (C) Ni^{2+}, based on the Phyre[2] structure and (D) Co^{2+}, (E) Cu^{2+}(SAS), and (F) Ni^{2+} based on the AlphaFold structure. Co atoms are shown in light blue, Cu in orange and Ni in green balls. C atoms are shown in green, O in red, N in blue and H in white sticks. The peptide fragment is represented as a grey or violet ribbon for the Phyre[2] and AlphaFold-based structures, respectively, with histidine sidechains protruding from it. Key distances are given in Å and χ1 dihedrals in °. In (A) and (D), close contact distances between the ligand and the peptide have been marked with red labels. For all the complexes with the peptide, close contacts are collected in **Table S2**.

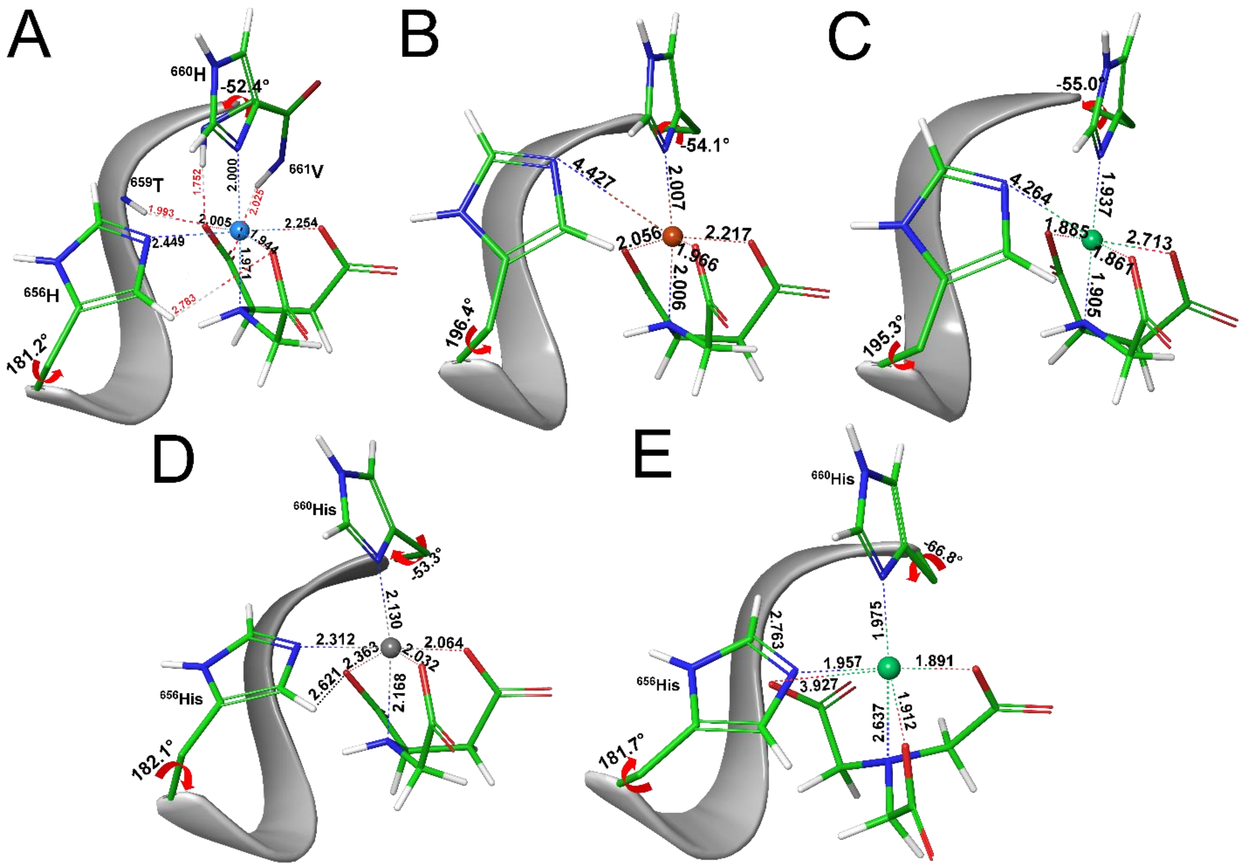

Fig.S6 Optimized structures with B3LYP and SAS of the peptide complexes based on the Phyre[2] structure of (A) Co-CM-Asp, (B) Cu-CM-Asp (repeated from **Fig.S5B**), (C) Ni-CM-Asp, (D) Zn-CM-Asp and (E) Ni-NTA. Co atoms are shown in light blue, Cu in orange, Zn in grey and Ni in green balls. C atoms are shown in green, O in red, N in blue and H in white sticks. The peptide fragment is represented as a grey ribbon with histidine sidechains protruding from it. Key distances are given in Å and χ1 dihedrals in °. In (A), close contact distances between the ligand and the peptide have been marked with red labels.

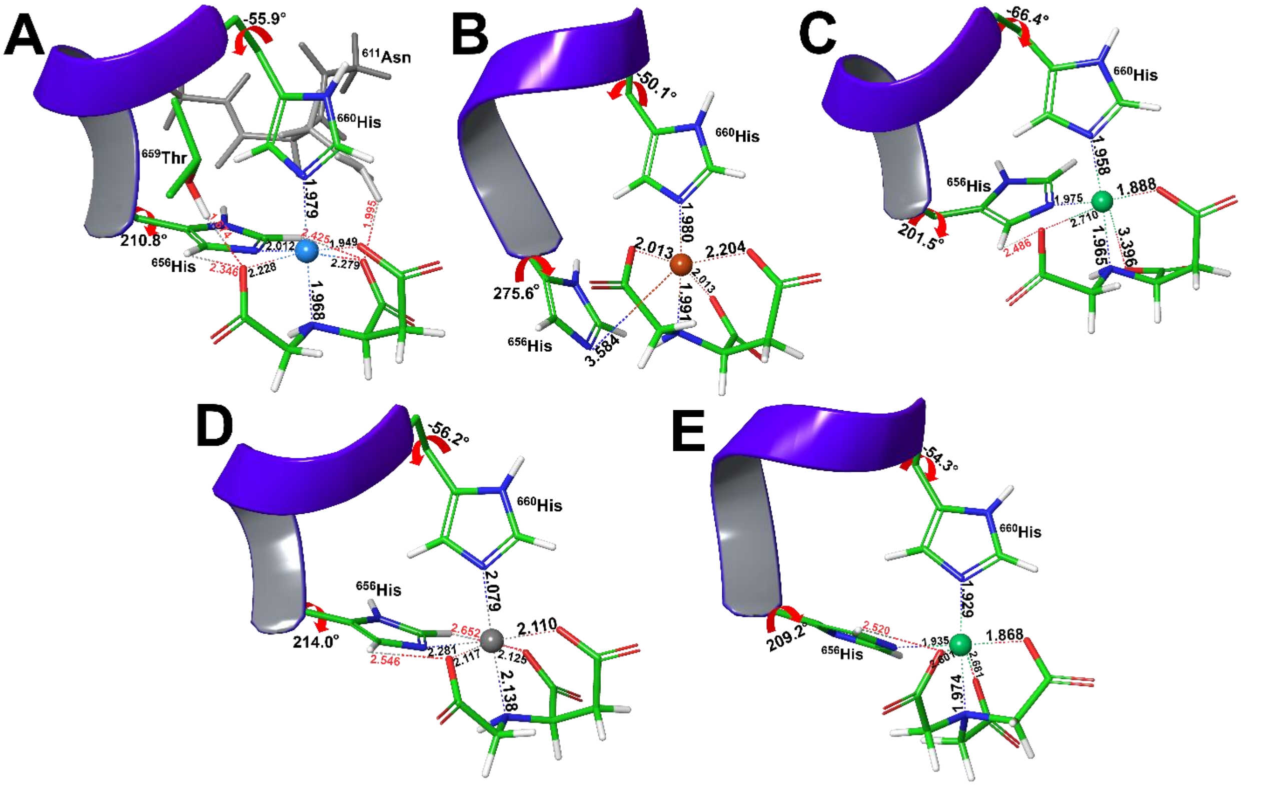

Fig.S7 Optimized structures with B3LYP and SAS of the peptide complexes based on the AlphaFold structure of (A) Co-CM-Asp, (B) Cu-CM-Asp (repeated from **Fig.S5E**), (C) Ni-CM-Asp, (D) Zn-CM-Asp and (E) Ni-NTA. Co atoms are shown in light blue, Cu in orange, Zn in grey and Ni in green balls. C atoms are shown in green, O in red, N in blue and H in white sticks. The peptide fragment is represented as a violet ribbon with histidine sidechains protruding from it. Key distances are given in Å and χ1 dihedrals in °. In (A), close contact distances between the ligand and the peptide have been marked with red labels. For all the complexes with the peptide, close contacts are collected in **Table S2**.

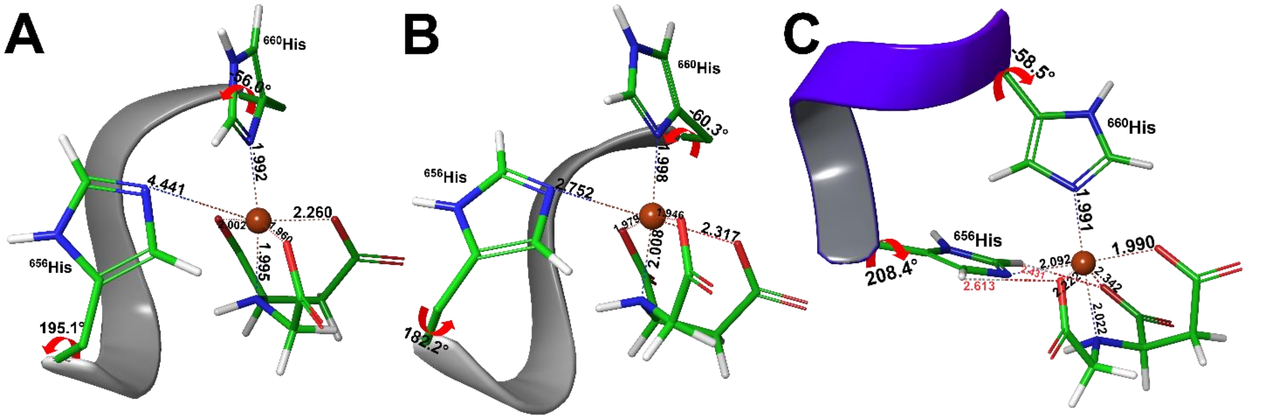

Fig.S8 Optimized structures of the peptide complexes with Cu and the MPWB1K and TPSSh functionals: (A) Cu-CM-Asp complex based on the Phyre[2] structure and optimized with TPSSh, (B) Cu-CM-Asp complex based on the Phyre[2] structure and optimized with MPWB1K (C) Cu-CM-Asp complex based on the AlphaFold structure and optimized with MPWB1K. Cu atoms are shown in orange balls, C atoms are shown in green, O in red, N in blue and H in white sticks. The peptide fragment is represented as a grey or violet ribbon for the Phyre[2] and AlphaFold-based structures, respectively, with histidine sidechains protruding from it. Key distances are given in Å and χ1 dihedrals in °. Close contact distances between the ligand and the peptide have been marked with red labels. For all the complexes with the peptide, close contacts are collected in **Table S2**.

Table S2 Average M-equatorial, M-axial, μMetal and ^{656}His-H···$O_{1,2eq}$ distances in Å, metal endocyclic angles and dihedral angles in ° of M-CM-Asp and Ni-NTA complexes with the protein fragment, based on the AlphaFold and Phyre^2 structural predictions and optimized with the B3LYP functional using PCM(SAS).

	Complex	μM-O_{eq}	μM-N_{eq},O_{eq}	μM-N_{ax},N'_{ax}	μM	μO_{eq}···H-^{656}H	χ_1 ^{656}H	χ_1 ^{660}H	θ_{OMOO}	Endocyclic	N'_{ax}-$\hat{M}$-N_{eq}
Phyre^2	Zn-CM-Asp	2.153	2.193	2.149	2.178	2.872	177.9	-53.2	102.9	338.9	87.1
	Ni-CM-Asp[b]	2.153	2.681	1.921	2.428	4.479	164.7	-55.0	110.3	353.5	71.0
	Co-CM-Asp	2.068	2.163	1.986	2.104	3.099	178.8	-52.4	98.5	349.6	88.8
	Cu-CM-Asp[b]	2.080	2.667	2.007	2.447	4.456	163.6	-54.1	132.8	352.4	66.0
	Ni-$NTA_{(1)}$[a]	2.577[c]	2.422	2.307	2.384	2.368	178.3	-66.8	116.5	320.1	92.1
AlphaFold	Zn-CM-Asp	2.117	2.158	2.109	2.142	2.599	146.0	-56.2	110.1	344.9	89.6
	Ni-CM-Asp[a]	2.665	2.492	1.962	2.315	3.105	158.5	-66.4	118.0	330.1	87.6
	Co-CM-Asp	2.152	2.117	1.974	2.069	2.385	149.2	-55.9	105.5	345.3	89.6
	Cu-CM-Asp[b]	2.077	2.454	1.986	2.298	3.203	84.4	-50.1	130.5	352.9	119.6
	Ni-$NTA_{(2)}$	2.383	2.271	1.952	2.165	2.443	150.8	-54.3	96.0	335.1	90.0

[a] tridentate coordination with respect to the ligand.
[b] coordinated only to ^{660}H.
[c] the values reported here disregard the axis rotation.

Table S3 Close contacts between the ligand and the peptide in the Phyre^2 and AlphaFold derived optimized complexes. These have been specified in **Fig.S5A**&**D** for the Co^{2+} complex, for the Phyre^2 and AlphaFold structures, respectively. The ^{656}H C-H···O_{eq} distances signify sidechain interaction, while all others involve interactions with the protein backbone. The last column (μBb.) includes the average interaction of all backbone close contacts to the O_{eq}. All values are given in Å.

	Complex	^{656}H C-H···O_{1eq}	^{659}T N-H···O_{eq}	^{660}H N-H···O_{eq}	^{661}V N-H···O_{eq}	μBb. (N-H···O_{eq})
Phyre^2	Zn-CM-Asp	2.713	1.985	1.804	1.923	1.904
	Cu-CM-Asp[a]	2.430	2.049	1.759	2.045	1.951
	Ni-CM-Asp	2.758	2.480	1.746	2.050	2.092
	Co-CM-Asp	2.867	2.101	1.765	2.005	1.957
	Ni-$NTA_{(1)}$	2.416	1.844	1.753	1.840	1.812
		**^{656}H C-H···O_{1eq}**	**^{659}T O-H···O_{1eq}**	**^{611}N N-H···O_{2eq}**	**^{656}H C-H···O_{2eq}**	**μSC.[b] ($O_{1,2eq}$)**
AlphaFold	Zn-CM-Asp	2.619	1.761	4.390	3.229	3.075
	Cu-CM-Asp[a]	3.372	1.701	1.825	3.034	1.763
	Ni-CM-Asp	2.428	1.707	5.940	3.319	3.823
	Co-CM-Asp	2.441	1.723	4.641	2.829	3.182
	Ni-$NTA_{(2)}$	2.696	1.755	1.935	2.669	1.845

[a] values are derived from the SAS-optimized calculation.
[b] values do not include interactions with ^{656}H.

Table S4 Metal-water M···OH_2 distances in the $[H_2O]_2M^{2+}$-Cm-Asp^{3-} and $[H_2O]_2Ni^{2+}$-NTA^{3-} complexes and their averages. All values are given in Å.

Complex	Axial	Equatorial	Average	$Average_{SAS}$
Zn-CM-Asp	2.188	2.223	2.205	2.266
Ni-CM-Asp	1.959	3.577	2.768	3.440
Co-CM-Asp	2.059	2.323	2.191	3.422
Cu-CM-Asp[a]	2.095	3.394	-	2.744
Ni-NTA	1.948	3.578	2.763	3.361

[a] values are from the SAS-optimized calculation.

Table S5 Collected structural information for all optimized complexes, including distances, close contacts with peptide backbone (Bb) and side chains (SC), angles, averages and coordination assignments (OCT: octahedral, TRG: trigonal bipyramidal, TWS: Twisted trigonal bipyramidal). Distances higher than 2.5 Å are highlighted in red, and in the coordination assignment the corresponding atom is shown in italics (123=O$_{eq}$, N=N$_{ax}$).

												Non AF >>>	^{656}H-H-O$_{eq1}$	^{656}H-H-O$_{eq2}$	^{659}T N-H...O$_{eq2}$	^{660}H N-H...O$_{eq2}$	^{661}V N-H...O$_{eq2'}$			
Model	M-O$_1$	M-O$_2$	M-O$_3$	M-N$_{ax}$	M-N$_{H656}$	M-N$_{H660}$	μM	N'$_{ax}$-M-N$_{eq}$	μM-O$_{eq}$	μM-N$_{eq}$,O$_{eq}$	μM-N$_{ax}$,N'$_{ax}$	Coordination	^{656}H-H-O$_{eq1}$	^{656}H-H-O$_{eq2}$	^{659}T O-H...O$_{eq1}$	^{611}N NH....O$_{eq2}$	-	μO$_{eq}$···H$_{im}$	μBb./SC.	Avg All
Co-AF$_{MPWB1K}$	2.240	2.198	1.946	1.966	1.981	1.976	2.051	90.1	2.128	2.091	1.971	OCT: 2His 123N	2.420	2.609	1.750	3.637	-	2.515	2.693	2.604
Co-P2$_{MPWB1K}$	2.184	2.228	1.943	1.994	1.983	2.006	2.056	91.8	2.118	2.085	2.000	OCT: 2His 123N	2.573	2.944	1.977	1.820	1.976	2.758	1.925	2.258
Co-P2$_{TPSSh}$	1.947	1.972	2.316	1.953	2.238	1.974	2.067	91.0	2.078	2.118	1.964	OCT: 2His 123N	2.862	3.201	2.160	1.746	1.986	3.032	1.964	2.391
Co-AF$_{B3LYP}$(SAS)	2.228	2.279	1.949	1.968	2.012	1.979	2.069	89.6	2.152	2.117	1.974	OCT: 2His 123N	2.346	2.425	1.674	1.995	-	2.385	1.834	2.110
Co-AF$_{B3LYP}$	2.303	2.276	1.961	1.979	1.980	1.967	2.078	88.6	2.180	2.130	1.973	OCT: 2His 123N	2.441	2.829	1.723	4.641	-	2.635	3.182	2.908
Co-P2$_{B3LYP}$	1.971	2.002	2.330	1.975	2.304	2.001	2.097	90.7	2.101	2.152	1.988	OCT: 2His 123N	2.867	3.298	2.101	1.765	2.005	3.082	1.957	2.407
Co-P2$_{B3LYP}$(SAS)	1.944	2.005	2.254	1.971	2.448	2.000	2.104	88.8	2.068	2.163	1.986	OCT: 2His 123N	2.783	3.414	1.993	1.752	2.025	3.099	1.924	2.394
Zn-AF$_{MPWB1K}$	2.131	2.080	2.145	2.140	2.164	2.065	2.121	92.0	2.119	2.130	2.103	OCT: 2His 123N	2.542	3.075	1.797	4.055	-	2.809	2.926	2.867
Zn-P2$_{TPSSh}$	2.054	2.165	2.115	2.174	2.188	2.098	2.132	92.1	2.111	2.131	2.136	OCT: 2His 123N	2.652	3.412	2.051	1.861	1.949	3.032	1.954	2.385
Zn-AF$_{B3LYP}$(SAS)	2.117	2.125	2.110	2.138	2.281	2.079	2.142	89.6	2.117	2.158	2.109	OCT: 2His 123N	2.546	2.652	1.713	2.025	-	2.599	1.869	2.234
Zn-P2$_{TPSSh}$	2.070	2.233	2.138	2.158	2.198	2.105	2.150	90.9	2.147	2.160	2.132	OCT: 2His 123N	2.696	3.203	2.000	1.784	1.910	2.949	1.898	2.318
Zn-AF$_{B3LYP}$	2.191	2.125	2.179	2.152	2.189	2.076	2.152	91.1	2.165	2.171	2.114	OCT: 2His 123N	2.619	3.229	1.761	4.390	-	2.924	3.075	3.000
Zn-P2$_{B3LYP}$	2.082	2.309	2.140	2.174	2.208	2.115	2.171	91.1	2.177	2.185	2.145	OCT: 2His 123N	2.713	3.267	1.985	1.804	1.923	2.990	1.904	2.338
Zn-P2$_{B3LYP}$(SAS)	2.032	2.363	2.065	2.168	2.311	2.130	2.178	87.1	2.153	2.193	2.149	OCT: 2His 123N	2.621	3.123	1.886	1.804	1.919	2.872	1.869	2.271
Cu-AF$_{MPWB1K}$	2.222	2.341	1.990	2.022	2.092	1.991	2.110	89.3	2.184	2.161	2.007	OCT: 2His 123N	2.613	2.430	1.751	1.994	-	2.522	1.873	2.197
Cu-P2$_{MPWB1K}$	1.946	1.979	2.317	2.008	2.752	1.998	2.167	86.3	2.081	2.249	2.003	*OCT* : *2His* 123N	2.816	4.250	2.275	1.835	2.072	3.533	2.061	2.650
Cu-AF$_{B3LYP}$(SAS)	2.013	2.013	2.204	1.991	3.584	1.980	2.298	119.6	2.077	2.454	1.986	TRG: 1His 123N	3.372	3.034	1.701	1.825	-	3.203	1.763	2.483
Cu-P2$_{TPSSh}$	1.960	2.001	2.260	1.995	4.441	1.992	2.442	67.8	2.074	2.666	1.994	TRG: 1His 123N	2.667	6.567	2.377	1.741	2.041	4.617	2.053	3.078
Cu-P2$_{B3LYP}$(SAS)	1.965	2.056	2.218	2.005	4.427	2.008	2.447	66.0	2.080	2.667	2.007	TRG: 1His 123N	2.430	6.482	2.049	1.759	2.045	4.456	1.951	2.953
Ni-AF$_{MPWB1K}$	2.448	3.297	1.867	1.946	1.914	1.918	2.232	88.6	2.537	2.382	1.932	TRG: 2His 13N	2.468	3.893	1.769	5.565	-	3.180	3.667	3.424
Ni-AF$_{B3LYP}$	2.667	3.436	1.911	1.980	1.947	1.950	2.315	88.7	2.671	2.490	1.965	TRG: 2His 13N	2.428	3.319	1.707	5.940	-	2.873	3.823	3.348
Ni-AF$_{B3LYP}$(SAS)	2.710	3.396	1.888	1.965	1.975	1.958	2.315	87.6	2.665	2.492	1.962	TRG: 2His 13N	2.486	3.724	1.646	8.256	-	3.105	4.951	4.028
Ni-P2$_{MPWB1K}$	1.850	1.862	2.598	1.893	4.218	1.915	2.389	72.8	2.103	2.632	1.904	TRG: 1His *123* N	2.801	6.356	2.414	1.789	2.098	4.579	2.100	3.092
Ni-P2$_{B3LYP}$(SAS)	1.861	1.885	2.714	1.905	4.264	1.937	2.428	69.2	2.153	2.681	1.921	TRG: 1His *123* N	2.615	6.343	2.173	1.765	2.032	4.479	1.990	2.986
Ni-P2$_{TPSSh}$	1.853	1.863	2.789	1.893	4.350	1.917	2.444	70.0	2.168	2.714	1.905	TRG: 1His *123* N	2.799	6.527	2.548	1.750	2.021	4.663	2.106	3.129
Ni-P2$_{B3LYP}$	1.867	1.875	2.855	1.906	4.228	1.936	2.445	71.0	2.199	2.706	1.921	TRG: 1His *123* N	2.758	6.360	2.480	1.746	2.050	4.559	2.092	3.079
Ni-NTA-AF$_{MPWB1K}$	2.560	2.517	1.865	1.960	1.918	1.913	2.122	89.4	2.314	2.215	1.937	*OCT:* 2His *12* 3N	2.688	2.548	1.752	1.933	-	2.618	1.842	2.230
Ni-AF$_{B3LYP}$	2.663	2.606	1.881	1.975	1.926	1.919	2.162	89.6	2.383	2.269	1.947	*OCT:* 2His *12* 3N	2.696	2.669	1.755	1.935	-	2.683	1.845	2.264
Ni-NTA-AF$_{B3LYP}$(SAS)	2.601	2.681	1.868	1.974	1.935	1.929	2.165	90.0	2.383	2.271	1.952	*OCT:* 2His *12* 3N	2.520	2.366	1.693	1.891	-	2.443	1.792	2.118
Ni-NTA-P2$_{MPWB1K}$	1.900	3.245	1.861	2.482	1.915	1.938	2.224	92.4	2.335	2.230	2.210	TWS: 2His 13*N*	2.411	2.558	1.856	1.770	1.894	2.485	1.840	2.098
Ni-NTA-P2$_{TPSSh}$	1.926	3.592	1.900	2.644	1.921	1.942	2.321	91.9	2.473	2.335	2.293	TWS: 2His 13*N*	2.401	2.807	1.833	1.734	1.822	2.604	1.797	2.119
Ni-NTA-P2$_{B3LYP}$	1.935	3.583	1.904	2.648	1.932	1.958	2.327	91.9	2.474	2.339	2.303	TWS: 2His 13*N*	2.416	2.754	1.844	1.753	1.840	2.585	1.812	2.121
Ni-NTA-P2$_{B3LYP}$(SAS)	1.912	3.928	1.891	2.638	1.958	1.975	2.384	92.1	2.577	2.422	2.307	TWS: 2His 13*N*	2.368	2.762	1.823	1.849	1.852	2.565	1.841	2.131
Co-imi$_{B3LYP}$	2.253	2.289	1.969	1.973	1.983	1.963	2.072	89.4	2.170	2.124	1.968	OCT: 2His 123N	2.443	2.383	2.744	2.835	-	2.413	-	2.601
Zn-imi$_{B3LYP}$	2.123	2.130	2.184	2.153	2.203	2.082	2.146	92.1	2.146	2.160	2.117	OCT: 2His 123N	2.702	2.575	2.709	3.143	-	2.639	-	2.782
Cu-imi$_{B3LYP}$	2.327	2.444	2.010	2.039	2.072	2.017	2.152	89.4	2.260	2.213	2.028	OCT: 2His 123N	2.540	2.344	2.627	3.435	-	2.442	-	2.737
Cu-imi$_{B3LYP}$(SAS)	2.360	2.397	1.973	2.035	2.098	2.033	2.149	89.0	2.243	2.207	2.034	OCT: 2His 123N	2.395	2.209	2.293	3.756	-	2.302	-	2.663
Ni-imi$_{B3LYP}$	2.715	3.358	1.900	1.970	1.939	1.971	2.309	90.1	2.658	2.478	1.971	TRG: 2His 13N	2.441	2.197	4.286	4.995	-	2.319	-	3.480
Ni-NTA$_{(1)B3LYP}$	1.940	3.974	1.911	2.626	1.932	1.930	2.386	91.1	2.608	2.439	2.278	TWS: 2His 13*N*	2.589	4.611	4.658	2.068	-	3.600	-	3.482
Ni-NTA$_{(2)B3LYP}$	2.652	3.201	1.870	1.984	1.913	1.934	2.259	91.0	2.575	2.409	1.959	*OCT:* 2His *12* 3N	2.630	2.243	4.345	4.531	-	2.436	-	3.437

4. Correlating SMARCA5 Computed Binding Energies with Experimental Binding Affinities

In an effort to interpret the experimental binding affinities, a focused benchmarking study was performed. The study comprised of three different DFT functionals combined with two structural predictions from the Phyre^2/I-TASSER (denoted as P2) and AlphaFold (denoted as AF) methodologies. A collection of energies was computed, including ΔE electronic energies, binding free energies ΔG_b, Counterpoise energies (CP) and basis set superposition error (BSSE) corrections. The latter are corrections to the computed binding energy and can only be performed in the gas phase in the current Gaussian 16 rev.C01. This is in contrast with the bulk of the calculations, performed in a water solvent continuum model (PCM – See Section 1).

With regards to the Counterpoise calculations, the molecule was split in two fragments comprising the metal-ligand and the binding pocket, illustrated in **Fig.S7**, and the energies of the fragments were evaluated both separately and together.

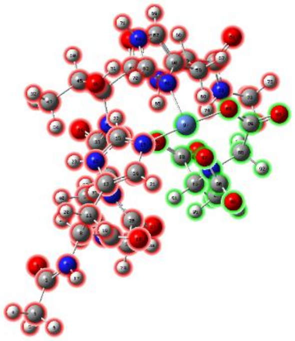

Fig.S9 Fragment setup for counterpoise calculations highlighting the two fragments, SMARCA5 in red (Phyre^2 structure) and Metal-CM-Asp in green.

The CP calculations were performed as single point calculations in the geometry of the PCM (or PCM/SAS for Cu^{2+}) optimized structures (CP), and also after full optimization in the gas phase ($CP_{(g)}$) – for the B3LYP Phyre^2 structures. The estimated BSSEs for each complex are included in **Table S6** and are taken from the single point calculation (CP).

Calculations were repeated for an additional two functionals (TPSSh and MPWB1K) and their CP, ΔE, ΔG_b values (for reactions (2) and (3) of the main text) are included in **Table S6**. For both these functionals, no need arose to optimize the structures using SAS.

In the calculations performed on the AlphaFold structure, apart from the metal-ligand and CH_3CO-HGATH-$NHCH_3$ fragments, residue ^{611}N was also included in the cluster since it features close to the dHis motif in the AlphaFold structure (see **Fig.3B**, left in the main text). This was inserted as a CH_3CO-^{611}N-$NHCH_3$ moiety, fixed at both ends at the C_a positions. As per the Phyre^2 B3LYP calculations, Cu-CM-Asp required SAS optimization with B3LYP and the scheme of re-optimizing all structures with PCM-SAS was repeated. Interestingly, optimization of Ni-NTA with the AlphaFold structure yielded the Ni-$NTA_{(2)}$ form unlike the Phyre^2 structure. Results from the AlphaFold calculations are included in **Tables S6**&**S7**.

Table S6 Different computed binding energies for the Metal-SMARCA5 complexes, computed by three different DFT functionals on the Phyre[2] (P2) and AlphaFold (AF) complexes. CP is the counterpoise corrected binding energy on the PCM (or SAS)-optimized geometry, BSSE is the estimated error at the same geometry, CP(g) is the energy after optimization in the gas phase, ΔE is the electronic energy of formation with PCM and SAS//PCM and ΔG_b is the free binding energy with PCM and SAS with additional columns for the inclusion of the BSSE corrections. $\Delta G_{b,w(SAS)}$ refers to the energies computed by the water exchange reaction (3) described in the main text. The binding free energies of the complexes with imidazole are included for reference in the last two columns. All values are given in kcal·mol^{-1}.

	Model	CP[a]	$CP_{(g)}$[a]	BSSE	ΔE	$\Delta E_{SAS//PCM}$	ΔG_b	ΔG_b[a]	$\Delta G_{b(SAS)}$	$\Delta G_{b(SAS)}$[a]	$\Delta G_{b,w}$	$\Delta G_{b,w(SAS)}$	ΔG_b[c]	$\Delta G_{b(SAS)}$[c]
**B3LYP-P2**	$Ni-NTA_{(1)}$	-143.0	-160.10	4.49	-42.25	-42.24	-22.67	-20.42	-33.21	-30.97	-22.47	-23.10	-11.90	-13.03
	Ni-CM-Asp	-79.1	-87.06	3.26	-40.96	-41.67	-23.31	-21.67	-31.58	-29.95	-16.86	-19.63	-8.06	-9.64
	Cu-CM-Asp	-75.6	-107.27	3.71	-	-43.98	-	-	-31.84	-29.98	-	-25.81	-13.34	-8.28
	Co-CM-Asp	-90.1	-82.32	3.58	-40.03	-41.34	-21.78	-19.99	-30.48	-28.69	-20.18	-18.97	-16.58	-18.13
	Zn-CM-Asp	-95.2	-122.53	3.72	-34.02	-35.10	-16.40	-14.54	-26.60	-24.74	-23.47	-22.34	-6.30	-9.94
**B3LYP-AF**	$Ni-NTA_{(2)}$	-120.5	-	3.34	-51.03	-80.07	-28.74	-27.07	-40.53	-38.78	-29.49	-27.19	-15.08	-11.37
	Ni-CM-Asp	-114.6	-	3.21	-42.69	-76.86	-23.71	-22.10	-29.56	-28.00	-17.26	-17.60	-	-
	Cu-CM-Asp	-73.9[b]	-	3.42[b]	-	-78.65	-	-	-37.88	-36.17	-	-31.86	-	-
	Co-CM-Asp	-88.0	-	3.00	-48.79	-81.94	-30.98	-29.48	-42.47	-40.88	-31.80	-32.17	-	-
	Zn-CM-Asp	-66.1	-	2.79	-37.18	-70.28	-19.05	-17.66	-34.32	-32.87	-26.12	-30.06	-	-
**MPWB1K-P2**	$Ni-NTA_{(1)}$	-	-	-	-40.20	-	-19.03	-	-	-	-12.63	-	-	-
	Ni-CM-Asp	-	-	-	-41.26	-	-22.58	-	-	-	-19.43	-	-	-
	Cu-CM-Asp	-	-	-	-41.24	-	-22.92	-	-	-	-21.75	-	-	-
	Co-CM-Asp	-	-	-	-41.33	-	-22.65	-	-	-	-21.69	-	-	-
	Zn-CM-Asp	-	-	-	-33.35	-	-14.06	-	-	-	-20.17	-	-	-
**MPWB1K-AF**	$Ni-NTA_{(2)}$	-	-	-	-9.98	-	-31.07	-	-	-	-24.67	-	-	-
	Ni-CM-Asp	-	-	-	-8.53	-	-23.59	-	-	-	-20.44	-	-	-
	Cu-CM-Asp	-	-	-	-9.14	-	-28.41	-	-	-	-27.24	-	-	-
	Co-CM-Asp	-	-	-	-9.54	-	-28.18	-	-	-	-27.22	-	-	-
	Zn-CM-Asp	-	-	-	-7.16	-	-17.51	-	-	-	-23.62	-	-	-
**TPSSh-P2**	$Ni-NTA_{(1)}$	-140.72	-	4.36	-39.55	-	-20.13	-17.95	-	-	-13.38	-	-	-
	Ni-CM-Asp	-75.65	-	3.02	-37.91	-	-21.29	-19.78	-	-	-14.81	-	-	-
	Cu-CM-Asp	-65.77	-	3.55	-40.44	-	-24.21	-22.43	-	-	-26.21	-	-	-
	Co-CM-Asp	-88.00	-	3.90	-41.48	-	-22.58	-20.63	-	-	-22.97	-	-	-
	Zn-CM-Asp	-89.80	-	3.58	-30.91	-	-12.99	-11.20	-	-	-20.26	-	-	-

[a] energies are corrected with ½ their respective BSSE.
[b] values computed using the SAS geometry.
[c] energies of the metal complexes with imidazole.

The CP and $CP_{(g)}$ energies are provided after correction with the BSSE, and the computed ΔG_b energies with solvation are also corrected with these values. This has been done for ΔG_b in the corresponding column of **Table S6** by adding half the BSSE value. This is recommended, since it has been reported that the BSSE is systematically overestimated by calculations (see the cited review).[1] For the functional MPWB1K, it was not possible to obtain the BSSE values.

The order of stability of the d-metal complexes, as predicted from the computed values in **Table S6**, is summarized in **Table S7** with the experimental binding affinities derived from **Fig.1C**&**1E** included in the first row. The Irving-Williams series is shown in the second row. This is relevant only for the H_2O exchange reaction, *i.e.* the $\Delta G_{b,w}$ $\Delta G_{b,w(SAS)}$ and $\Delta G_{b,w+1/2.BSSE}$ rows. Since the series is valid only for a single ligand, Ni-NTA should be excluded when comparing the stability of the complexes with the Irving-Williams series.

[1] : Ulf Ryde, Par Soderhjelm Chem. Rev. **2016**, *116*, 5520−5566.

For the B3LYP functional, in almost all cases, the computed binding energies of the Ni-NTA complex are overestimated with respect to the experimental affinities, while those of Zn^{2+} tend to be underestimated. However, the ΔG_b values of the imidazole and AlphaFold complexes point to Co^{2+} as the most stable complex, as per the experimental findings.

Additionally, the counterpoise single point calculations (based on the PCM-optimized geometry – or PCM-SAS for Cu^{2+}) fix the stability of the Zn^{2+} complex in the series and improve that of Co^{2+} with respect to the experimental findings – but only for the P2-derived complexes. Applying the BSSE corrections to the ΔG_b values does not alter the relative stability in any case.

Regarding the additional two functionals, none manages to reproduce entirely the experimental affinities. Both overestimate the Cu^{2+} binding with respect to Co^{2+} and Zn^{2+} complexes. Concerning the H_2O exchange reaction, B3LYP/P2 reproduces the Irving-Williams Series more faithfully, since both TPSSh and MPWB1K (P2 and AF structures) underestimate Ni^{2+} / overestimate Co^{2+} binding.

Finally, regarding the AlphaFold models, energetically, the advantage of Ni-NTA over Ni-CM-Asp is duplicated with both B3LYP and MPWB1K functionals (**Table S7**, $\Delta G_{b,w}$ values). Structurally, unlike the Phyre[2] structure (shown in **Fig.S6C**), Ni-CM-Asp is tridentate with Ni^{2+} coordinated to both histidines (**Fig.S7C**). Also, unlike the Ni-NTA$_{(1)}$ Phyre[2] structure (**Fig.S6E**), the AlphaFold Ni-NTA$_{(2)}$ structure is tetradentate and coordinated to the 2nd histidine in a closer to octahedral fashion (**Fig.S7E**). Overall, the Phyre[2] structures interact with the peptide backbone through residues T659 and V661, while the AlphaFold structures interact with the side chains of residues T659 and N611 (**Table S3**).

Table S7 The d-block metals arranged in order of increasing complex binding stability determined by the different sets of calculations. Co, Zn, Ni and Cu refer to the complexes with CM-Asp. TMs are color-coded for easier perusal.

Exp. Binding Affinity[a]		Co > Zn > Cu > Ni-NTA > Ni
Irving-Williams series[b]		Co < Ni < Cu >> Zn
**B3LYP-P2**	CP	Ni-NTA$_{(1)}$ >> Zn > Co >> Ni > Cu
	CP$_{(g)}$	Ni-NTA$_{(1)}$ >> Zn >> Cu >> Ni > Co
	ΔE	Ni-NTA$_{(1)}$ >> Ni > Co >> Zn
	$\Delta E_{SAS//PCM}$	Cu > Ni-NTA$_{(1)}$ > Ni ≈ Co >> Zn
	ΔG_b	Ni ≈ Ni-NTA$_{(1)}$ > Co > Zn
	$\Delta G_{b+1/2\cdot BSSE}$	Ni > Ni-NTA$_{(1)}$ ≈ Co > Zn
	$\Delta G_{b(SAS)}$	Ni-NTA$_{(1)}$ > Cu ≈ Ni > Co > Zn
	$\Delta G_{b(SAS)+1/2\cdot BSSE}$	Ni-NTA$_{(1)}$ > Cu = Ni > Co > Zn
	$\Delta G_{b,w(SAS)}$	Cu > Ni-NTA$_{(1)}$ ≈ Zn > Ni ≈ Co
	ΔG_b (imidazole)	Co > Cu > Ni-NTA$_{(1)}$ > Ni > Zn
	$\Delta G_{b(SAS)}$ (imidazole)	Co > Ni-NTA$_{(1)}$ >> Zn ≈ Ni > Cu
**B3LYP-AF**	CP	Ni-NTA$_{(2)}$ > Ni >> Co >> Cu >> Zn
	ΔE	Ni-NTA$_{(2)}$ > Co > Ni > Zn
	$\Delta E_{SAS//PCM}$	Co > Ni-NTA$_{(2)}$ > Cu > Ni >> Zn
	ΔG_b	Co > Ni-NTA$_{(2)}$ >> Ni > Zn
	$\Delta G_{b+1/2\cdot BSSE}$	Co > Ni-NTA$_{(2)}$ >> Ni > Zn
	$\Delta G_{b(SAS)}$	Co > Ni-NTA$_{(2)}$ > Cu > Zn > Ni
	$\Delta G_{b(SAS)+1/2\cdot BSSE}$	Co > Ni-NTA$_{(2)}$ > Cu > Zn > Ni
	$\Delta G_{b,w(SAS)}$	Co ≈ Cu > Zn > Ni-NTA$_{(2)}$ >> Ni
**MPWB1K-P2**	ΔE	Co ≈ Ni ≈ Cu > Ni-NTA$_{(1)}$ >> Zn
	ΔE_w	Cu ≈ Co ≈ Zn > Ni Ni-NTA$_{(1)}$
	ΔG_b	Cu ≈ Co ≈ Ni > Ni-NTA$_{(1)}$ >> Zn
	$\Delta G_{b,w}$	Cu ≈ Co > Zn > Ni >> Ni-NTA$_{(1)}$
**MPWB1K-AF**	ΔE	Ni-NTA$_{(2)}$ ≈ Co ≈ Cu > Ni > Zn
	ΔE_w	Co > Cu > Ni-NTA$_{(2)}$ ≈ Zn > Ni
	ΔG_b	Ni-NTA$_{(2)}$ > Cu ≈ Co >> Ni > Zn
	$\Delta G_{b,w}$	Cu = Co > Ni-NTA$_{(2)}$ > Zn > Ni
**TPSSh-P2**	CP	Ni-NTA$_{(1)}$ >> Zn ≈ Co >> Ni >> Cu
	ΔE	Co > Cu ≈ Ni-NTA$_{(1)}$ > Ni >> Zn
	ΔE_w	Co ≈ Cu > Zn >> Ni-NTA$_{(1)}$ > Ni
	ΔG_b	Cu > Co > Ni > Ni-NTA$_{(1)}$ >> Zn
	$\Delta G_{b+1/2\cdot BSSE}$	Cu > Co > Ni > Ni-NTA$_{(1)}$ >> Zn
	$\Delta G_{b,w}$	Cu > Co > Zn >> Ni > Ni-NTA$_{(1)}$
	$\Delta G_{b,w+1/2\cdot BSSE}$	Cu > Co > Zn >> Ni > Ni-NTA$_{(1)}$

[a] experimental relative affinities were derived from **Figs. 1C** & **1E**.
[b] this series applies only to the CM-Asp complexes and the $\Delta G_{b,w}$, $\Delta G_{b,w(SAS)}$ and $\Delta G_{b,w+1/2\cdot BSSE}$ rows.

5. Verification of SMARCA5's Identity after Isolation

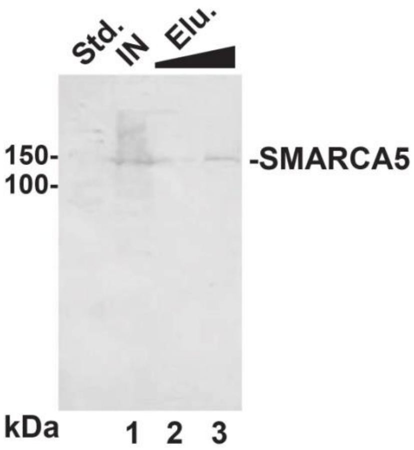

Fig.S10 The Sf9 cell extract, containing expressed recombinant FLAG-tagged SMARCA5 (IN, lane1), was loaded onto a CM-Asp-Co- agarose column. The column was washed and the retained material eluted with imidazole. The immunoblot analysis of SMARCA5 with the anti-SNF2h/ISWI polyclonal antibody (Cat. No. A301-017A, Bethyl Lab.) was performed by using the enhanced chemiluminescence system (Thermo Scientific). Lanes 2 and 3 show SMARCA5 present in 5 and 10 μl of eluate (Elu.), respectively.

6. Predictions of Mg^{2+} - Binding Site and Double Histidine Structural Motif

Table S8 The full-length human SMARCA5 amino acid sequence was submitted to the I-TASSER server for prediction of ligand-binding sites. The authentic table is presented, showing the Mg^{2+}-coordinating residue Thr212 in the last row. Residues involved in the ADP binding are listed in the top row.

Click to view	Rank	C-score	Cluster size	PDB Hit	Lig Name	Download Complex	Ligand Binding Site Residues
○	1	0.46	20	5jc3A	ADP	Rep, Mult	179,181,184,207,208,209,210,211,213,247,595
◉	2	0.10	5	5f9fE	BU3	Rep, Mult	203,205,209,211,214,335,336
○	3	0.03	1	N/A	N/A	N/A	183,206,207,208,210,211,212,238,239,240,243,244,247,308,309,312,337,338,341,394,396,397,454,507,531,538,539,559,560,562,563,564,565,580,581,582,584,585,587,588,591,592,595,596,628,633
○	4	0.02	1	5e3hA	BEF	Rep, Mult	207,211,309,564
○	5	0.02	1	4kitB	MG	Rep, Mult	212

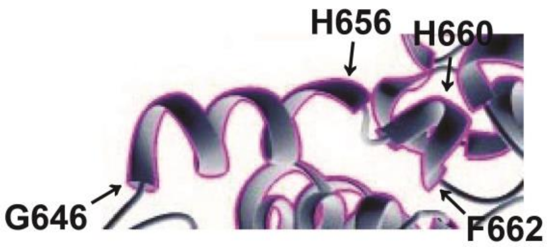

Fig.S11 The full-length human SMARCA5 amino acid sequence was submitted to the I-TASSER server for secondary structure prediction. Positions of His656 and His660 are indicated within the α-helix structure, spanning from Gly646 to Phe662.

7. Coordinates of all optimized species with the B3LYP functional

Zn^{2+}[CM-Asp]$^{3-}$ tridentate

O	-0.18340000	-2.05697700	2.04947900
O	-2.26834400	-1.27961600	1.72339300
O	0.22367100	1.89955500	2.13817800
O	-1.95250400	2.29261800	2.11539300
O	3.25406600	0.98721100	0.08841500
O	3.77515500	0.06574300	-1.87205300
N	0.93806600	-0.48494700	0.29014900
C	-0.53722500	-0.45900400	0.30106400
C	-1.10929100	0.96668900	0.31582800
C	1.60836300	-0.41834200	-1.01164600
C	-1.04630800	-1.34168700	1.48054100
C	-0.96462000	1.76549900	1.61448800
C	2.99793300	0.24363600	-0.94000900
H	-0.90853500	-0.94275500	-0.60902500
H	-0.63453200	1.54592800	-0.48368300
H	-2.16916700	0.91148100	0.08765700
H	1.18270300	-1.33452100	0.80151200
H	1.71436000	-1.40207100	-1.47045900
H	1.01204100	0.19565400	-1.68978000
Zn	1.77013500	1.10481000	1.35425600

Zn^{2+}[CM-Asp]$^{3-}$ tridentate SAS

O	-0.17345400	-2.05290100	1.89351800
O	-2.31225300	-1.50417400	1.40032500
O	0.33103500	2.14370500	2.01963500
O	-1.84213500	2.12354600	2.45260300
O	3.18857500	1.14639600	-0.05729100
O	3.87500000	-0.10636500	-1.77426200
N	0.92305900	-0.39929300	0.29271800
C	-0.55995700	-0.37163000	0.24481400
C	-1.17743900	1.02744100	0.42858000
C	1.68084800	-0.55423000	-0.95566400
C	-1.08340700	-1.41463500	1.30016200
C	-0.92084900	1.79117600	1.73986200
C	3.03898800	0.19125900	-0.94700800
H	-0.89012100	-0.73665400	-0.73141900
H	-0.84925400	1.67706500	-0.39323800
H	-2.25353200	0.90409800	0.34679100
H	1.07209300	-1.19439500	0.94314900
H	1.87158500	-1.60110700	-1.19412400
H	1.10743900	-0.13084600	-1.78383600
Zn	1.67634500	1.32044800	1.05838400

Zn^{2+}[CM-Asp]$^{3-}$K^{+} tridentate

O	-0.30541900	-1.95057800	2.11378100
O	-2.26440800	-0.89465700	1.86502800
O	0.00295200	1.61142000	2.19732800
O	-1.92194700	2.56867400	1.65062000
O	3.11926800	1.05497600	0.20003700
O	3.73608400	0.33065400	-1.81419000
N	0.90530600	-0.57571400	0.24558600
C	-0.56918700	-0.49430000	0.22725800
C	-1.07485300	0.94890800	0.11224500
C	1.62316800	-0.41988400	-1.02302000
C	-1.10160200	-1.18769000	1.50886400
C	-1.02279200	1.77958000	1.39652600
C	2.94063100	0.36731900	-0.88137000
H	-0.95744700	-1.05831800	-0.62784700
H	-0.49833900	1.47987100	-0.65225500
H	-2.10696900	0.94342800	-0.22504700
H	1.12770200	-1.45750000	0.70232700
H	1.84258100	-1.38179000	-1.48617500
H	1.00592000	0.14306500	-1.72481400
Zn	1.66583100	0.92215600	1.49395000
K	-0.94399700	-0.25518100	4.30169200

Zn^{2+}[CM-Asp]$^{3-}$ tetradentate

O	0.13051600	-1.26827500	2.54574100
O	-1.98188600	-1.72922800	1.98696500
O	0.51883800	1.62570800	1.78054400
O	-1.11771500	2.95511900	1.08717400
O	3.16422500	0.36353500	0.67526100
O	3.67836000	0.80192200	-1.45008400
N	0.79250700	-0.78409600	-0.02332800
C	-0.63869900	-0.68443800	0.30614000
C	-1.18697500	0.74571700	0.25692500
C	1.46082100	0.03705600	-1.02826300
C	-0.86229800	-1.28871400	1.72663900
C	-0.54910200	1.85877600	1.11002000
C	2.89154400	0.42612400	-0.58180500
H	-1.24486800	-1.28246000	-0.37807400
H	-1.19105200	1.10504300	-0.77395900
H	-2.23662000	0.69438400	0.54687900
H	1.04002900	-1.75720400	-0.14936500
H	1.52195300	-0.45037100	-2.00169200
H	0.92044800	0.97176500	-1.16826200
Zn	1.59253900	-0.08146100	1.81624000

Zn^{2+}[CM-Asp]$^{3-}$ tetradentate SAS

O	0.21672500	-1.23582200	2.52082700
O	-1.90089600	-1.77589600	2.02789000
O	0.51019300	1.69497000	1.74339100
O	-1.20685200	2.94962000	1.09364400
O	3.16358800	0.24433500	0.71988900
O	3.76177300	0.65781100	-1.39641600
N	0.76438500	-0.77516000	-0.08240100
C	-0.65598400	-0.68373800	0.30034800
C	-1.21689000	0.74313000	0.26486000
C	1.47700600	0.06682400	-1.03844100
C	-0.81608300	-1.29666800	1.73724000
C	-0.59659900	1.88825400	1.10552000
C	2.93520300	0.34475900	-0.55563900
H	-1.28596400	-1.29201800	-0.35361100
H	-1.24097500	1.10228000	-0.76674200
H	-2.25975200	0.67391200	0.57327800
H	1.03551900	-1.74379700	-0.18148100
H	1.52459000	-0.35602000	-2.04316800
H	0.98343900	1.03534400	-1.10995800
Zn	1.51013800	0.01678200	1.72466600

Zn^{2+}[CM-Asp]$^{3-}$[H_2O]$_2$tetradentate SAS

O	0.23440900	-1.49833100	2.33577700
O	-1.90517100	-1.91259200	1.80774000
O	0.55670600	1.50747000	1.83797000
O	-1.08542700	2.90236500	1.27520700
O	3.23199900	0.31939500	0.43745600
O	3.56998300	1.00555100	-1.67081100
N	0.80134500	-0.70195300	-0.15422200
C	-0.62328200	-0.67224400	0.22202500
C	-1.20502400	0.74105200	0.33074300
C	1.39288500	0.21480500	-1.12549600
C	-0.80140800	-1.43848700	1.57101300
C	-0.53251000	1.80923300	1.22042000
C	2.87137300	0.54043700	-0.77858900
H	-1.23557200	-1.21181500	-0.50654100
H	-1.30065900	1.18528100	-0.66229200
H	-2.22549200	0.62991100	0.69850100
H	1.06256700	-1.65410400	-0.37564100
H	1.34997200	-0.15475800	-2.15216800
H	0.85938400	1.16337700	-1.09857000
Zn	1.70906700	-0.16959200	1.72716000
O	2.88163400	1.28174600	3.03853300
H	3.50537900	1.39069200	2.30329900
H	2.14843600	1.89525100	2.83902600
O	2.83058200	-1.89783500	2.63699300
H	1.98095800	-2.37267900	2.67595600
H	3.37198000	-2.32271700	1.96234700

Zn^{2+}[CM-Asp]$^{3-}$K^{+} tetradentate

O	0.13315300	-1.25813300	2.56518400
O	-1.98370100	-1.73197300	2.03744800
O	0.54429800	1.63605700	1.66930400
O	-1.04313600	2.96694100	0.87458200
O	3.09108300	0.48101300	0.60684800
O	3.55952400	0.86653600	-1.53883600
N	0.77206800	-0.83762500	-0.02477200
C	-0.65772500	-0.72917600	0.31940700
C	-1.20986500	0.69882600	0.23508500
C	1.41100800	-0.04835000	-1.07477600
C	-0.86846800	-1.29821500	1.75549200
C	-0.52464700	1.85440500	0.98540500
C	2.80947500	0.46027700	-0.65513000
H	-1.26950800	-1.34556900	-0.34240500
H	-1.27871900	1.00778800	-0.80920100
H	-2.24030900	0.66279400	0.59045700
H	1.01360400	-1.81462900	-0.13140400
H	1.51056900	-0.59965400	-2.00963100
H	0.82176800	0.84130100	-1.29106800
Zn	1.58335800	-0.11058500	1.79473700
K	2.78293200	3.22957700	1.54445100

Co^{2+}[CM-Asp]$^{3-}$ tridentate

O	-0.06593300	-1.99486400	1.95330800
O	-2.21283800	-1.41336500	1.61091400
O	0.22517500	1.79760400	2.12019000
O	-1.94759700	2.20980100	2.28789800
O	3.26308800	0.89173100	0.08126100
O	3.89462400	-0.22503000	-1.74911500
N	0.94030100	-0.28658500	0.27915400
C	-0.54082300	-0.37010100	0.26376300
C	-1.20942100	1.00070100	0.35303200
C	1.61985400	-0.43093900	-1.02373600
C	-0.98853100	-1.34830000	1.39401100
C	-0.99739000	1.72055800	1.68457500
C	3.05390200	0.09086200	-0.91757700
H	-0.84530400	-0.83433100	-0.67816400
H	-0.82494900	1.64316900	-0.44570300
H	-2.27725000	0.88331600	0.19512700
H	1.19464200	-1.06897300	0.90024200
H	1.60802700	-1.46477500	-1.37005600
H	1.10065800	0.18002100	-1.76423800
Co	1.71232900	1.27840500	1.10880800

Co^{2+}[CM-Asp]$^{3-}$ tridentate SAS

O	0.00268400	-2.01900900	1.82866800
O	-2.18639000	-1.56167500	1.48472300
O	0.30842600	1.96344700	1.98159900
O	-1.85724900	2.28778500	2.34880400
O	3.23615400	0.92380100	0.09326700
O	3.94048500	-0.29117500	-1.65421600
N	0.92527900	-0.25152500	0.27097800
C	-0.55786100	-0.33408400	0.24100500
C	-1.27118800	1.00371700	0.41318200
C	1.63903600	-0.46348000	-1.00224300
C	-0.97335700	-1.41283200	1.30904700
C	-0.96849000	1.79218700	1.68933200
C	3.07437800	0.05763800	-0.88215900
H	-0.85148000	-0.74253700	-0.73003900
H	-1.02899400	1.66345100	-0.42822000
H	-2.34030700	0.81127100	0.39320200
H	1.11621800	-1.04174100	0.93437000
H	1.63338500	-1.51504500	-1.29287300
H	1.14791100	0.10797000	-1.79257300
Co	1.71392300	1.28074200	1.07784500

Co^{2+}[CM-Asp]$^{3-}$K^{+} tridentate

O	0.14272900	-2.35445500	1.66570900
O	-2.02379300	-1.76381100	1.57575000
O	0.12283900	1.47306900	2.28519800
O	-2.07859700	1.53473200	2.53925700
O	3.21740200	1.01151700	0.17732200
O	3.88027700	0.24983400	-1.81644000
N	0.99569100	-0.35933400	0.21898700
C	-0.47248700	-0.55988800	0.21324800
C	-1.23578400	0.74490400	0.43677900
C	1.64722200	-0.24353900	-1.10177500
C	-0.81647700	-1.66255800	1.25980000
C	-1.08260300	1.29129300	1.85412600
C	3.04116900	0.36411000	-0.93389800
H	-0.76030600	-0.96353700	-0.76152600
H	-0.87709900	1.49982200	-0.26887400
H	-2.29019700	0.58335700	0.23373000
H	1.33801000	-1.19870600	0.70541400
H	1.70310400	-1.20843300	-1.60597400
H	1.06376900	0.43338700	-1.72802100
Co	1.65914300	1.14044100	1.25932100
K	-3.89619900	-0.48866900	2.89706100

Co^{2+}[CM-Asp]$^{3-}$ tetradentate

O	0.10936900	-1.15256800	2.53291400
O	-1.97569000	-1.74910800	1.98720800
O	0.58648100	1.59381300	1.76008200
O	-1.10042500	2.93064000	1.20537300
O	3.06599100	0.45680800	0.68688300
O	3.64107600	0.88831800	-1.42830900
N	0.81256100	-0.80225000	0.01769000
C	-0.64286600	-0.67666300	0.30105000
C	-1.15900600	0.76526200	0.26125900
C	1.46187800	-0.00857300	-1.04321600
C	-0.88262600	-1.26336500	1.71290500
C	-0.50889700	1.84419700	1.15302700
C	2.84739600	0.48028100	-0.58465500
H	-1.22458800	-1.26016700	-0.41486000
H	-1.13647600	1.14416300	-0.76223900
H	-2.21527300	0.72883700	0.52829800
H	1.02602900	-1.78378200	-0.12525100
H	1.55454400	-0.57131100	-1.97108900
H	0.87071900	0.87915000	-1.25589600
Co	1.57236800	-0.18478100	1.72252500

Co^{2+}[CM-Asp]$^{3-}$ tetradentate SAS

O	0.11612300	-1.08901100	2.51904200
O	-1.93848600	-1.81025000	1.97225900
O	0.58330000	1.59827400	1.83852600
O	-1.11089300	2.92097600	1.26517700
O	2.99350100	0.52513700	0.69429000
O	3.64676000	0.86842600	-1.42209200
N	0.80769900	-0.82863300	-0.00663400
C	-0.64827900	-0.67389700	0.29242500
C	-1.13254400	0.78051300	0.27579200
C	1.47304100	-0.05222300	-1.07378900
C	-0.87791300	-1.26824600	1.70683200
C	-0.50812100	1.85224700	1.21152200
C	2.83731700	0.49076200	-0.59052500
H	-1.25026400	-1.24108600	-0.42202700
H	-1.08166600	1.18679400	-0.73689700
H	-2.19485100	0.75107200	0.51780900
H	1.00638800	-1.81570300	-0.12756600
H	1.61148900	-0.63974800	-1.98128300
H	0.86240400	0.81085200	-1.32938700
Co	1.50756000	-0.10735500	1.68022500

Co^{2+}[CM-Asp]$^{3-}$[H_2O]$_2$tetradentate SAS

O	1.23877300	-1.52898000	1.87454400
O	-0.68845700	-2.60436900	1.47477700
O	0.66240000	1.32277000	1.85890200
O	-1.45044000	2.04037800	1.97856600
O	3.22674600	1.28503200	0.19536500
O	3.19804200	2.36647200	-1.76937100
N	1.26796200	-0.36805700	-0.46628400
C	-0.07790500	-0.81919000	0.00116100
C	-1.01392900	0.31967400	0.41597600
C	1.42278300	0.83503100	-1.31373400
C	0.15958300	-1.75413900	1.21067800
C	-0.57307600	1.31128200	1.52259200
C	2.73745700	1.56929800	-0.97007300
H	-0.57334100	-1.39728000	-0.78240200
H	-1.29784400	0.91424300	-0.45543600
H	-1.93547400	-0.14801100	0.76144200
H	1.71524900	-1.15404500	-0.92786400
H	1.38890800	0.59814900	-2.37713300
H	0.61524500	1.52937800	-1.09603400
Co	2.17617000	0.02945700	1.18309200
O	2.97419000	0.70529500	2.93411400
H	3.59077000	1.38434100	2.62462700
H	2.13539700	1.18371700	3.10727100
O	0.41848900	-3.47702400	3.95853400
H	1.10346300	-2.83254400	3.73695700
H	-0.15328600	-3.37817400	3.17488600

Co^{2+}[CM-Asp]$^{3-}$ K^{+} tetradentate

O	0.05737500	-1.17604300	2.48094400
O	-2.02260100	-1.75911300	1.89965400
O	0.44587900	1.51916700	1.88244600
O	-1.04028400	2.95086700	1.05410100
O	2.97466900	0.52973500	0.71408800
O	3.61458700	0.98679800	-1.37621100
N	0.81649900	-0.82074700	-0.00430700
C	-0.64806700	-0.69246600	0.23931600
C	-1.15238700	0.75466300	0.19032700
C	1.47850900	-0.03839800	-1.07154100
C	-0.92476800	-1.28500800	1.63916100
C	-0.52938500	1.82852400	1.10796600
C	2.81033600	0.53588600	-0.56812100
H	-1.20988500	-1.26836700	-0.49779200
H	-1.11085800	1.13587100	-0.83040900
H	-2.21455100	0.72255200	0.43720400
H	1.03087600	-1.80421400	-0.13658400
H	1.64347400	-0.64147000	-1.96270400
H	0.85474500	0.80460000	-1.35885700
Co	1.53119000	-0.21813000	1.69423400
K	-0.40080300	0.81843800	4.47722300

Cu^{2+}[CM-Asp]$^{3-}$ tridentate

O	-0.10740600	-1.98853600	1.95545900
O	-2.25202400	-1.44041700	1.54413700
O	0.27295600	1.88530100	2.09741100
O	-1.90884700	2.15429300	2.35773600
O	3.22428900	0.98458800	0.04402500
O	3.89905800	-0.17101800	-1.73923300
N	0.92345000	-0.33709900	0.26385200
C	-0.55512900	-0.37343100	0.25083400
C	-1.19554600	1.01330500	0.37351500
C	1.64035000	-0.48102900	-1.01385500
C	-1.02370000	-1.36035000	1.36625000
C	-0.96550800	1.72785900	1.70700500
C	3.04471000	0.12785600	-0.92101200
H	-0.87807500	-0.80148500	-0.70158900
H	-0.81523400	1.65822800	-0.42473500
H	-2.26686000	0.90935500	0.23184800
H	1.16749500	-1.09139600	0.92369100
H	1.70291500	-1.52569100	-1.31851900
H	1.10211800	0.06569800	-1.78966700
Cu	1.69355200	1.30287300	1.07654400

Cu^{2+}[CM-Asp]$^{3-}$ tridentate SAS

O	-0.06063800	-1.98797400	1.85168200
O	-2.23400300	-1.60273800	1.35666900
O	0.32330800	2.00118500	2.03302500
O	-1.84698200	2.18166700	2.45193900
O	3.21603500	1.02896000	-0.00875200
O	3.93061600	-0.26595900	-1.68514900
N	0.91056300	-0.28668900	0.26149900
C	-0.56806500	-0.32569400	0.22149800
C	-1.24311400	1.03202700	0.44041400
C	1.66350100	-0.53497700	-0.97591800
C	-1.01746800	-1.41711900	1.26149400
C	-0.94284400	1.77414800	1.75099700
C	3.06826400	0.09229500	-0.90998200
H	-0.87568700	-0.68554000	-0.76379000
H	-0.97096500	1.70796200	-0.37918800
H	-2.31669500	0.87063200	0.39956900
H	1.09066400	-1.02535300	0.98172600
H	1.74614100	-1.60257400	-1.18462800

H	1.14324400	-0.06978300	-1.81570700
Cu	1.68668900	1.37442800	0.99630100

Cu^{2+}[CM-Asp]$^{3-}$ K^{+} tridentate

O	-0.26915800	-1.69305500	2.14828900
O	-2.33032700	-1.56859600	1.25090000
O	0.23084500	1.76087000	2.14356900
O	-1.95837800	2.02035400	2.36295900
O	3.19156500	1.00755800	0.02995400
O	3.90662200	-0.15963100	-1.72894900
N	0.90219900	-0.35126200	0.23500700
C	-0.57845900	-0.37106900	0.17899800
C	-1.18999000	1.03342200	0.31526900
C	1.64739800	-0.50461300	-1.02686700
C	-1.11898500	-1.31156600	1.29435800
C	-1.00117800	1.65618000	1.69908400
C	3.03833500	0.13293100	-0.92495500
H	-0.88333300	-0.77866600	-0.78566100
H	-0.75022300	1.69575400	-0.43545000
H	-2.25603200	0.96794200	0.11811800
H	1.14769300	-1.07561000	0.91781500
H	1.73376900	-1.55242800	-1.31237100
H	1.11236000	0.01954200	-1.81990300
Cu	1.66657300	1.28932200	1.06947400
K	0.37577600	-0.27160500	4.34040800

Cu^{2+}[CM-Asp]$^{3-}$ tetradentate

O	0.15289100	-1.19565900	2.52726300
O	-1.95168900	-1.73399200	2.01760400
O	0.52376900	1.61372800	1.86338900
O	-1.06630900	2.96885400	1.10722000
O	3.04467200	0.45480300	0.71576600
O	3.62999900	0.93535300	-1.37947200
N	0.79224800	-0.83812900	-0.04357100
C	-0.64391200	-0.68225800	0.30071800
C	-1.14718800	0.76715600	0.26974600
C	1.46651900	-0.02181900	-1.06172300
C	-0.85814500	-1.27092600	1.71971800
C	-0.50857100	1.86476300	1.15414300
C	2.83252600	0.49295400	-0.56228500
H	-1.26414600	-1.25661000	-0.38926900
H	-1.13597000	1.14072200	-0.75525100
H	-2.20171200	0.72970200	0.54508000
H	1.00202100	-1.81887600	-0.18167000
H	1.61085900	-0.57297000	-1.98975800
H	0.86851900	0.85644600	-1.29565300
Cu	1.55618300	-0.17433900	1.72170400

Cu^{2+}[CM-Asp]$^{3-}$ tetradentate SAS

O	0.15697200	-1.15448700	2.53499200
O	-1.91861900	-1.80059600	2.00662300
O	0.53967800	1.63702800	1.84856200
O	-1.05810600	2.96911500	1.06944500
O	3.04102000	0.44205800	0.73540200
O	3.66806100	0.85466300	-1.36935300
N	0.77642500	-0.84878500	-0.07640200
C	-0.65104900	-0.69457600	0.29723400
C	-1.15147200	0.75623000	0.27556700
C	1.46549400	-0.00528900	-1.05791600
C	-0.84933800	-1.28707400	1.72388800
C	-0.50275800	1.87533400	1.13730400
C	2.85386900	0.46604300	-0.54875100
H	-1.29351000	-1.26905900	-0.37467900
H	-1.16641400	1.12982700	-0.75011200
H	-2.19772700	0.71914700	0.58049500
H	0.99297600	-1.82574700	-0.21833000
H	1.59794000	-0.50446800	-2.01823800
H	0.88397500	0.89702600	-1.23816900
Cu	1.51514800	-0.09748800	1.72613500

Cu^{2+}[CM-Asp]$^{3-}$[H_2O]$_2$tetradentate SAS

O	-0.66587900	-1.20334900	2.56004600
O	-2.75982900	-0.80259900	1.87016300
O	0.34228100	1.60092200	1.90998900
O	-0.56287200	3.29890200	0.76166100
O	2.83339300	-0.37078200	1.14973400
O	3.73060800	-0.09176600	-0.88126000
N	0.38966400	-0.87964100	0.18111500
C	-1.00908600	-0.39263700	0.29587200
C	-1.17885100	1.11474200	0.09384400
C	1.35719400	-0.29711200	-0.75543500
C	-1.55608300	-0.82638700	1.68696400
C	-0.39713700	2.10290800	0.99776600
C	2.77645800	-0.25712800	-0.13972700
H	-1.63351800	-0.89655400	-0.44685300
H	-0.97876300	1.38746700	-0.94392000
H	-2.23779000	1.32220500	0.25274400
H	0.38598800	-1.89633500	0.12478500
H	1.38912600	-0.83388900	-1.70427200
H	1.08398500	0.73480200	-0.96539500
Cu	1.09329600	-0.49391000	1.99765400
O	1.82719300	0.45190300	3.71665300
H	2.75020000	0.59439600	3.46476200
H	1.34526400	1.24817400	3.38959700
O	0.01258100	-3.63741900	1.31401000
H	0.74214900	-4.11316200	1.72315300
H	-0.27182000	-2.97085200	1.97005700

Cu^{2+}[CM-Asp]$^{3-}$K^{+} tetradentate

O	0.15532100	-1.24742700	2.52585400
O	-1.97306200	-1.73000700	2.06199700
O	0.51614700	1.57524600	1.81776100
O	-0.92884900	2.97829700	0.88224100
O	2.96905100	0.50211000	0.66775200
O	3.51924800	0.96886100	-1.43982800
N	0.76031100	-0.90355600	-0.05102100
C	-0.67294500	-0.73297900	0.30889200
C	-1.16986200	0.71781700	0.24931900
C	1.41886300	-0.11086800	-1.09900400
C	-0.87479200	-1.30004500	1.73878900
C	-0.47326900	1.84226100	1.04560600
C	2.75315300	0.49123500	-0.61694900
H	-1.30224400	-1.31714200	-0.36391700
H	-1.21757800	1.05092500	-0.78772400
H	-2.20680100	0.70014300	0.58864000
H	0.95927400	-1.88836900	-0.17652400
H	1.60077300	-0.70584700	-1.99224100
H	0.78948600	0.72665900	-1.39316900
Cu	1.54294500	-0.24052400	1.69750900
K	2.71554100	3.19405400	1.73280600

Ni^{2+}[CM-Asp]$^{3-}$ tridentate

O	-0.04092400	-2.00571000	1.92382600
O	-2.19334400	-1.40695900	1.64238200
O	0.25130500	1.73487700	2.10045400
O	-1.89064400	2.27847200	2.26030600
O	3.21716700	0.88506000	0.12196200
O	3.90827200	-0.18864300	-1.71175400
N	0.93582100	-0.27880300	0.27515300
C	-0.54628300	-0.36743500	0.26158300
C	-1.22370800	0.99483100	0.34591800
C	1.61731700	-0.42429400	-1.02980100
C	-0.97575900	-1.34989900	1.39631400
C	-0.97595800	1.72564500	1.66212600
C	3.04307900	0.09959400	-0.90022000
H	-0.83959800	-0.83814800	-0.68035700
H	-0.86647600	1.63336000	-0.46763100
H	-2.29480300	0.86747800	0.22124000
H	1.18908500	-1.05856900	0.90605000
H	1.60163600	-1.46063800	-1.36713200
H	1.10117400	0.18642900	-1.77149000
Ni	1.68520700	1.23225600	1.09476900

Ni^{2+}[CM-Asp]$^{3-}$ tridentate SAS

O	0.02303400	-2.01615900	1.80356200
O	-2.17255300	-1.57117300	1.48332500
O	0.30802100	1.84648800	2.04243200
O	-1.83382500	2.36503900	2.31074200
O	3.20623600	0.93103900	0.09241600
O	3.94606500	-0.26963400	-1.64954000
N	0.92380700	-0.24011300	0.26851100
C	-0.56072300	-0.32353400	0.23694100
C	-1.27489400	1.00994900	0.41615800
C	1.64430500	-0.47560600	-1.00054800
C	-0.96316400	-1.41252400	1.29881400
C	-0.95701600	1.79271000	1.69434500
C	3.07022200	0.06794700	-0.88096400
H	-0.84571100	-0.72724000	-0.73842900
H	-1.04336900	1.67249900	-0.42576600
H	-2.34468900	0.82000100	0.40607300
H	1.11080100	-1.01862000	0.95411700
H	1.64625200	-1.53611500	-1.25594900
H	1.14651900	0.06790300	-1.80549800
Ni	1.67324900	1.27604900	1.03295700

Ni^{2+}[CM-Asp]$^{3-}$[H_2O]$_2$tridentate SAS

O	-0.23481700	-2.37799200	1.28235300
O	-2.48192100	-2.34626500	1.01877800
O	-0.56814800	1.33892800	2.34890500
O	-2.70890600	1.21544500	2.90100300
O	2.29835600	1.37999800	0.00580300
O	3.06876800	0.55372700	-1.92495100
N	0.23653100	-0.16782300	0.14216700
C	-1.20718100	-0.53102500	0.13389600
C	-2.12426200	0.56073200	0.67217900
C	0.88964600	-0.11022200	-1.17948500
C	-1.33740700	-1.89672000	0.90409900
C	-1.82112300	1.05348300	2.08439700
C	2.21047300	0.63955900	-1.05825900
H	-1.49845100	-0.73431300	-0.89905500
H	-2.08267700	1.43516600	0.01265300
H	-3.14126500	0.17845800	0.66324900
H	0.59999700	-1.00073300	0.66966800
H	1.04345700	-1.10906300	-1.59014200
H	0.25888800	0.44833200	-1.87389800
Ni	0.81426900	1.32338900	1.13248500
O	1.44631500	2.86887200	2.21896900
H	2.34622100	2.78082100	2.55677200
H	0.82489000	2.78852100	2.96344600
O	4.96751000	2.52750300	-1.14638800
H	4.42352400	2.75022100	-0.38311800
H	4.44498500	1.80731100	-1.54724800

Ni^{2+}[CM-Asp]$^{3-}$K^{+} tridentate

O	-0.03435700	-2.02584600	1.89637900
O	-2.14813300	-1.27573100	1.73251700
O	0.27525100	1.75645500	2.06371700
O	-1.89551300	2.13037700	2.29708500
O	3.24667600	0.88350500	0.09533000
O	3.93648700	-0.18797600	-1.73984500
N	0.97086700	-0.28449000	0.25443800
C	-0.51081300	-0.36995900	0.24576200
C	-1.17262100	1.00247000	0.30320900
C	1.64657200	-0.42675400	-1.05520100
C	-0.93990900	-1.31260500	1.41182200
C	-0.94933500	1.68861300	1.64600200
C	3.07207200	0.09844200	-0.92826400
H	-0.81151100	-0.86428700	-0.68147000
H	-0.76752600	1.63935600	-0.48757000
H	-2.23943600	0.89854200	0.13015900
H	1.23581900	-1.06325900	0.87779300
H	1.63026600	-1.46252400	-1.39357000
H	1.12618100	0.18502200	-1.79283800
Ni	1.71815200	1.24288400	1.06082900
K	-3.87692400	0.31093700	2.92540600

Ni^{2+}[CM-Asp]$^{3-}$ tetradentate

O	0.24657800	-1.32307800	2.47709300
O	-1.95131700	-1.53093600	2.15891600
O	0.41538200	1.59175600	1.79083600
O	-1.01793000	3.01241200	0.81800000
O	3.11188800	0.23251300	0.72346800
O	3.64384200	0.99710200	-1.30440100
N	0.81939200	-0.79161900	0.01870900
C	-0.64239500	-0.67727000	0.34040300
C	-1.21020900	0.73492700	0.21786900
C	1.44801000	0.10145300	-0.98325800
C	-0.83393300	-1.22664100	1.76587600
C	-0.53769900	1.87689700	1.01724000
C	2.85853200	0.47714600	-0.52540800
H	-1.19815500	-1.32147900	-0.34286200
H	-1.25677800	1.03116000	-0.83092000
H	-2.24841600	0.67566700	0.54902100
H	1.00863800	-1.75541400	-0.24074100
H	1.47089000	-0.36382700	-1.96711300
H	0.88033400	1.02408200	-1.06052900
Ni	1.69591100	-0.50594700	1.66149900

Ni^{2+}[CM-Asp]$^{3-}$ tetradentate SAS

O	0.08876200	-1.08770100	2.46180200
O	-1.97308600	-1.77595000	1.87998600
O	0.53861400	1.57145600	2.28207300
O	-1.23011000	2.79504700	1.72413800
O	2.59180200	0.90906300	0.54455300
O	3.13137800	1.31816500	-1.58772000
N	0.82817500	-0.98396800	-0.01833000
C	-0.60400200	-0.60537500	0.26323900
C	-0.86523700	0.92349300	0.31924800
C	1.52030000	-0.38838600	-1.17305200
C	-0.90752500	-1.23626400	1.64219700
C	-0.49868700	1.82632400	1.55431100
C	2.50075400	0.71785200	-0.73466200
H	-1.25865900	-1.03366900	-0.50022200
H	-0.40190400	1.40344500	-0.54723400
H	-1.93841900	1.05231800	0.19068900
H	0.89783700	-1.99402800	-0.02627700
H	2.10211700	-1.15871800	-1.67839600
H	0.81825000	0.01575900	-1.90346400
Ni	1.36220300	-0.00996000	1.59081800

Ni^{2+}[CM-Asp]$^{3-}$K^{+} tetradentate

O	0.29837000	-1.42584400	2.41757400
O	-1.92254100	-1.55775500	2.22348500
O	0.03244100	1.56601500	2.09619500
O	0.04372700	2.76496400	0.20833600
O	2.89408100	0.51567000	0.64124900
O	3.37109200	1.21215800	-1.42388300
N	0.76478100	-0.84903000	-0.01485500
C	-0.68776800	-0.71133400	0.34642200
C	-1.20672000	0.72460200	0.23261500
C	1.35955500	-0.04198800	-1.11121100
C	-0.82383000	-1.27320600	1.77655000
C	-0.31069300	1.78288000	0.90122700
C	2.65681100	0.61327800	-0.63941300
H	-1.27846800	-1.34073600	-0.32051500
H	-1.36575200	0.98473500	-0.81197400
H	-2.18284600	0.73703900	0.72081300
H	0.94865700	-1.83231800	-0.19407600
H	1.54546300	-0.66519400	-1.98330300
H	0.68293600	0.75684100	-1.40081000
Ni	1.66053300	-0.48945900	1.59179200
K	2.27162700	3.16448000	1.83422300

Ni^{2+}[NTA]$^{3-}$ tridentate

O	1.79131400	-1.39981100	-2.44013400
O	-1.75599800	-0.64463400	-3.12749000
O	0.40444000	3.37220900	-0.29275600
O	3.14926400	-1.57045500	-0.67602600
O	-3.60995800	-0.17051400	-1.97681900
O	-0.17301900	1.63962600	1.02351900
N	-0.11584700	-0.16021400	-1.16394000
C	0.89475400	-0.84437800	-0.30735000
C	-1.54201800	-0.35726900	-0.77652400
C	0.20689300	1.28698000	-1.34064400
C	2.06786800	-1.29828200	-1.17150000
C	-2.40749800	-0.36995300	-2.03372800
C	0.13371700	2.17558500	-0.06297200
H	0.44095600	-1.73604600	0.12352300
H	1.20714500	-0.19380700	0.50404400
H	-1.85232100	0.40582800	-0.06896900
H	-1.64222900	-1.33145400	-0.29926400
H	-0.47730800	1.69523000	-2.08176100
H	1.21255100	1.35783900	-1.75060200
Ni	0.03320400	-1.00304300	-2.84019600

Ni^{2+}[NTA]$^{3-}$ tridentate SAS

O	1.77378800	-1.40168300	-2.45226700
O	-1.74434100	-0.68704300	-3.12974200
O	0.42931600	3.35689500	-0.27221000
O	3.15071700	-1.55886600	-0.69355000
O	-3.60879400	-0.18732900	-1.99484200
O	-0.18311900	1.60312500	1.02055100
N	-0.11796900	-0.15372000	-1.16249000
C	0.89743000	-0.83890600	-0.30855300
C	-1.54903400	-0.34157500	-0.77947100
C	0.21091000	1.29041500	-1.34374200
C	2.07185700	-1.29436900	-1.17922100
C	-2.41343600	-0.38369800	-2.04277800
C	0.14041400	2.17416200	-0.04007700
H	0.44232100	-1.72987700	0.12401300
H	1.19932600	-0.18265000	0.50258500
H	-1.84958200	0.43770200	-0.08515100
H	-1.65016700	-1.30392900	-0.27784500
H	-0.47832100	1.70285500	-2.07761800
H	1.21732000	1.35760500	-1.75157700
Ni	0.02727500	-1.00567700	-2.81560300

Ni^{2+}[NTA]$^{3-}$K^{+} tridentate

O	1.79373900	-1.62077500	-2.38185100
O	-1.66306500	-0.52921400	-3.15334000
O	-0.98630400	2.79871500	-0.18382100
O	3.12247100	-1.84844800	-0.60262700
O	-3.38052300	0.38983600	-2.05557000
O	0.85112200	2.00547000	0.84059500
N	-0.01385200	-0.19981400	-1.17336400
C	0.94419400	-0.90639500	-0.27450700
C	-1.45070300	-0.32882900	-0.80893100
C	0.36656900	1.23686100	-1.37309400
C	2.07313600	-1.49797200	-1.11477700
C	-2.26561800	-0.11718700	-2.08342400
C	0.05732200	2.10024100	-0.11425700
H	0.42482900	-1.73029500	0.21284800
H	1.31606400	-0.22333200	0.48551700
H	-1.72708400	0.36726100	-0.02086500
H	-1.62924000	-1.34598400	-0.46268000
H	-0.19565900	1.61434700	-2.22226100
H	1.42897400	1.26336700	-1.60329000
Ni	0.08747300	-1.08270600	-2.83025300
K	-3.45034500	3.03160000	-1.06107800

Ni^{2+}[NTA]$^{3-}$ tetradentate

O	1.25704700	-1.39302800	-2.83042500
O	-1.55794500	-0.60950500	-3.13134400
O	-0.02441100	2.07033600	-2.65310900
O	1.93132600	-3.00025200	-1.43701000
O	-3.03283200	-1.47436800	-1.68061400
O	-0.18395400	3.66855200	-1.10026500
N	0.18370100	0.11452400	-0.93047600
C	1.09918600	-0.96570000	-0.48845400
C	-1.25246300	-0.23942700	-0.75855000
C	0.47824800	1.46889000	-0.40210400
C	1.45531800	-1.89442300	-1.65156400
C	-1.99994000	-0.84851200	-1.97787800
C	0.04177300	2.51486100	-1.43363900
H	0.67331900	-1.53234800	0.33864900
H	2.03136500	-0.51850500	-0.14480400
H	-1.79526800	0.67463400	-0.51851000
H	-1.35540400	-0.91394400	0.08984400
H	1.55548100	1.57026000	-0.27274700
H	-0.00198800	1.64125900	0.56126800
Ni	0.50301000	0.29909700	-2.83007000

Ni^{2+}[NTA]$^{3-}$ tetradentate SAS

O	1.16482000	-1.48758800	-2.78813400
O	-1.51607800	-0.43548000	-3.15806100

O	0.02524300	1.99889300	-2.72261000
O	1.95925500	-3.00397100	-1.34728200
O	-3.19166500	-1.03039900	-1.79457700
O	-0.18423000	3.64693600	-1.22347700
N	0.19402900	0.09105100	-0.85215400
C	1.17727900	-0.92829600	-0.45031500
C	-1.21396300	-0.35800200	-0.72532100
C	0.41220000	1.47776600	-0.40819500
C	1.45778900	-1.92341300	-1.59598300
C	-2.04838400	-0.63493100	-2.02465400
C	0.03469700	2.48570700	-1.51415700
H	0.86372400	-1.47388400	0.44190700
H	2.12387500	-0.43487300	-0.22611200
H	-1.77388800	0.39239800	-0.16786200
H	-1.24177900	-1.28099000	-0.14689300
H	1.47448100	1.61893500	-0.20455800
H	-0.13874000	1.71035800	0.50533200
Ni	0.42690500	0.20218600	-2.84869600

$Ni^{2+}[NTA]^{3-}K^{+}$ tetradentate

O	1.16971600	-1.57926700	-2.72919200
O	-1.88447500	-0.17940900	-2.89233600
O	0.04728600	1.95028500	-2.70083200
O	2.10866300	-3.00292600	-1.28545900
O	-3.37436200	-0.23151000	-1.21555100
O	-0.36982700	3.55774200	-1.20944300
N	0.22564800	0.01317700	-0.90561000
C	1.31699100	-0.89250300	-0.45513800
C	-1.10505300	-0.61253900	-0.66170200
C	0.29745600	1.40676200	-0.39123900
C	1.56211300	-1.94304900	-1.54235000
C	-2.22589400	-0.28845100	-1.68111900
C	-0.05506600	2.41285500	-1.48778400
H	1.08228400	-1.35597100	0.50280800
H	2.23306200	-0.31326200	-0.34516600
H	-1.43554600	-0.35068800	0.34269000
H	-0.97221200	-1.69352700	-0.70304900
H	1.32100700	1.61619600	-0.08279100
H	-0.34989000	1.53882700	0.47425500
Ni	0.50989000	0.14908400	-2.79215600
K	-2.28492200	2.05544000	-4.31854600

$Zn^{2+}[CM\text{-}Asp]^{3-}[imidazole]_2$

O	-0.07434400	-0.22872300	3.04212700
O	-2.13195800	-0.93659400	2.52845200
O	0.42995200	2.03889600	1.11774100
O	-1.05812200	2.91101000	-0.29429000
O	3.17028400	0.65390900	0.56933900
O	3.63065000	0.11115600	-1.55030600
N	0.85419500	-0.81217500	0.56985600
C	-0.60316900	-0.66683900	0.71788900
C	-1.16639300	0.57142200	0.01561800
C	1.50955100	-0.54844500	-0.70906700
C	-0.97215300	-0.61550700	2.22768100
C	-0.54259400	1.94956700	0.30764700
C	2.89535600	0.11925100	-0.55040300
H	-1.12657200	-1.53537300	0.30755500
H	-1.15052800	0.43446100	-1.06698700
H	-2.22261200	0.63725100	0.28043700
H	1.12548900	-1.72775500	0.90577600
H	1.62825600	-1.45199300	-1.30999000
H	0.91121200	0.14427400	-1.29751400
Zn	1.66813800	0.50864400	2.06284400
C	2.34474100	-1.94016000	3.95225900
C	4.13820100	-1.40186000	2.86818000
C	4.47818400	-2.44436900	3.68266300
H	4.74263900	-0.85456000	2.16720500
H	5.40264300	-2.97073700	3.83653700
C	3.55119800	2.63674500	3.10774200
C	1.98506400	2.34879300	4.57790600
C	2.82837300	3.29276500	5.08877300
H	1.08970900	1.93270300	5.00445100
H	4.59548100	4.09089000	4.20799700
H	2.81928200	3.84528000	6.01038100
N	2.80651300	-1.09982800	3.04759300
N	3.32720100	-2.77155400	4.36222300
H	3.23113100	-3.50603900	5.04470600
H	1.33320500	-1.96846000	4.31673900
N	2.44921700	1.95155400	3.34379800
N	3.81265300	3.46031500	4.14266600
H	4.15987100	2.55618100	2.22440100

$Co^{2+}[CM\text{-}Asp]^{3-}[imidazole]_2$

O	-0.17496000	-0.17809300	3.11748300
O	-2.13986700	-1.07431900	2.51100000
O	0.67497100	1.98182300	1.15096300
O	-0.95552800	2.98342300	0.03190900
O	3.29409000	0.63079500	0.41302700
O	3.64058000	-0.20964000	-1.63687800
N	0.92766700	-0.76487100	0.70860400
C	-0.54830900	-0.64667000	0.78967300
C	-1.06982800	0.62396500	0.11960100
C	1.54279100	-0.64697100	-0.62840300
C	-0.99408700	-0.64854300	2.27635500
C	-0.40282700	1.96121300	0.47014700
C	2.95991200	-0.03164500	-0.60909400
H	-1.01955000	-1.50518300	0.30452400
H	-1.03001600	0.53298000	-0.96780800
H	-2.12919100	0.71440400	0.36023400
H	1.17802500	-1.67260600	1.08568300
H	1.57570500	-1.61405800	-1.13110800
H	0.93976300	0.01450000	-1.24642000
C	2.28072900	-1.69830900	3.85806300
C	4.05326900	-1.22642500	2.69502600
C	4.40715400	-2.22153200	3.55954300
H	4.63331600	-0.71720300	1.94808800
H	5.33433800	-2.74103300	3.71867700
C	3.55841000	2.48976000	3.12007900
C	1.88741200	2.19950700	4.47291800
C	2.71178200	3.11433900	5.06323200
H	0.95694300	1.78635800	4.81884200
H	4.54997500	3.89682400	4.32697900
H	2.64896800	3.64496200	5.99551300
N	2.72519100	-0.91260600	2.89330400
N	3.27358200	-2.50555200	4.28508400
H	3.19255000	-3.19988700	5.00990000
H	1.27877200	-1.69157500	4.24703300
N	2.42975000	1.82358200	3.26451300
N	3.76218300	3.28431900	4.19240600
H	4.22379700	2.41627600	2.27848100
Co	1.69248200	0.52178600	1.99345200

$Cu^{2+}[CM\text{-}Asp]^{3-}[imidazole]_2$

O	-0.31803500	-0.53667200	3.06383600
O	-2.32410600	-1.10434900	2.23242000
O	0.44134500	2.00926400	1.34065300
O	-1.17718900	2.98948700	0.18786600
O	3.28468100	0.66083200	0.42658800
O	3.69409600	-0.24222800	-1.58513700
N	0.88895600	-0.74650800	0.66038600
C	-0.58642200	-0.66028900	0.67301500
C	-1.10593400	0.63215500	0.04578600
C	1.55787600	-0.63144600	-0.64545900
C	-1.12309300	-0.78660500	2.12668400
C	-0.57785300	1.97324300	0.57003100
C	2.98136800	-0.02740600	-0.58575300
H	-1.00897100	-1.49576600	0.10703000
H	-0.93966700	0.63538500	-1.03361300
H	-2.18816500	0.64132500	0.17004000
H	1.14942000	-1.63305500	1.07744500
H	1.60310500	-1.59865000	-1.14800800
H	0.98182000	0.03427000	-1.28517200
C	2.26402700	-1.79747500	3.88012500
C	4.06633200	-1.18879100	2.83331700
C	4.43059200	-2.19005700	3.68632800
H	4.65686900	-0.62580100	2.13399100
H	5.37571800	-2.66301800	3.88146700
C	3.55943000	2.54213900	3.05348600
C	2.04018800	2.26549900	4.57805100
C	2.92844100	3.17591900	5.07373600
H	1.15063600	1.86584400	5.03130900
H	4.68215100	3.94466800	4.14300700
H	2.96852100	3.70927500	6.00567400
N	2.71473700	-0.95865600	2.96621500
N	3.27666000	-2.56078900	4.33891800
H	3.19722400	-3.28216800	5.03736500
H	1.24129200	-1.86276200	4.20558500
N	2.44814300	1.88413500	3.32033100
N	3.88062900	3.33649200	4.09455500
H	4.13037400	2.45827800	2.14564500
Cu	1.62474800	0.54837800	2.05288400

$Ni^{2+}[CM\text{-}Asp]^{3-}[imidazole]_2$

O	-0.69031700	-1.64842400	2.70621800
O	-2.22791900	-0.05722700	2.30881900
O	0.72147800	2.05776600	1.28048400
O	-0.86792300	3.17225600	0.23765900
O	3.61165800	0.27532800	0.12704900
O	3.80157900	-1.69918100	-0.92742100
N	0.90668500	-0.68995600	0.77628700
C	-0.56073100	-0.49549300	0.63257300
C	-0.92345500	0.81892900	-0.02788700
C	1.61110200	-0.85032600	-0.51846000
C	-1.22475800	-0.73785800	2.02099700
C	-0.33600500	2.10153700	0.54292400
C	3.15313600	-0.75937400	-0.41821300
H	-0.93620100	-1.30248700	-0.00996200
H	-0.64190100	0.80472400	-1.08426300
H	-2.00547200	0.91940200	-0.00492100
H	0.98612200	-1.55852400	1.30162500
H	1.31638000	-1.80197300	-0.96293400
H	1.29223300	-0.04734400	-1.18128200
C	2.14447100	-1.70001300	3.75401400
C	4.02465300	-1.05185700	2.85317900
C	4.32270300	-2.08294500	3.69225400
H	4.65496900	-0.47553400	2.20261800
H	5.25172900	-2.56597000	3.93269400
C	2.51056200	3.20920900	3.09189900
C	3.02094000	1.67640400	4.54736100
C	3.42713300	2.86616700	5.07214000
H	3.09749600	0.69287700	4.96977600
H	3.26513700	4.80861700	4.21212100
H	3.90442900	3.11394300	6.00222700
N	2.66374100	-0.82533800	2.90434800
N	3.12579000	-2.47455800	4.24880400
H	2.99681900	-3.22655000	4.90654300
H	1.09549400	-1.79441800	3.97792800
N	2.44886500	1.90436300	3.31031600
N	3.09764600	3.81818100	4.13589500
H	2.13127400	3.70974700	2.22116200
Ni	1.70040000	0.60999300	2.02605300

$Ni^{2+}[NTA]^{3-}[imidazole]_2$ (1)

C	10.54350600	84.54113000	23.72806100
N	10.75636700	85.71500200	23.04362600
C	11.57523600	84.43338800	24.61186900
C	11.87848500	86.28775100	23.51115100
N	12.39873300	85.53189200	24.46640400
H	11.79530800	83.65923700	25.32239800
H	12.28030900	87.21670800	23.15012900
H	10.17388600	86.08800200	22.31109200
C	14.44141900	88.79794400	25.25507700
N	13.64254400	87.72157400	25.58745400
C	13.79776400	89.94064800	25.62891600
C	12.54387800	88.19941500	26.15180600
N	12.60751900	89.54110300	26.19391700
H	14.06901900	90.97666200	25.54227400
H	11.71667100	87.62153000	26.52226900
H	11.89912700	90.14564400	26.57772500
O	16.81988400	87.97687200	23.42617000
O	16.25318500	88.14934300	21.26231300
O	15.75245300	86.16701500	26.23286600
O	17.94882200	85.97556300	26.39015400
O	14.36523500	83.93806500	25.46888700
O	14.83479200	81.99591300	24.50643600
N	15.83227700	85.28801000	23.49697400
C	15.99025300	86.03147500	22.25045800
C	17.02102600	85.24611800	24.34193100
C	15.29174600	83.96405300	23.24061300
C	16.38662300	87.52112900	22.34156400
C	16.89990900	86.84453200	25.74918500
C	14.80858500	83.22940300	24.49279300
H	15.03934500	85.98899100	21.71828900
H	17.35182400	84.21054600	24.48364700
H	17.84506400	85.77451800	23.86914800
H	16.73384200	85.55138600	21.59034300
H	14.42012800	84.06246100	22.58691300
H	16.00791700	83.30710400	22.72712400
Ni	14.07765200	85.85426500	25.36701200
H	9.69945700	83.90672200	23.52999700
H	15.38692700	88.65208500	24.75618500

$Ni^{2+}[NTA]^{3-}[imidazole]_2$ (2)

C	10.85820900	84.62463000	23.64369200
N	10.98170200	85.88085200	23.09656000
C	11.94711900	84.45908800	24.44708100
C	12.10916300	86.44783300	23.56138800
N	12.71553300	85.60426600	24.38229300
H	12.24074300	83.62133700	25.05204500
H	12.49355300	87.42007900	23.29468100
H	10.34047600	86.30839800	22.44772300
C	13.75433300	87.58319700	27.79980800
N	13.27073800	86.73633700	26.81989400
C	12.74558700	87.87305200	28.66797700
C	11.99237200	86.52043600	27.09392500
N	11.64515200	87.19129300	28.20497700
H	12.71247200	88.48696800	29.54902700
H	11.31406900	85.90492500	26.53348200
H	10.72892300	87.18678300	28.62379800
O	14.45529100	88.50673000	23.32165900
O	16.69386300	88.30460900	23.34212000
O	15.86490700	86.61071200	26.21274900
O	18.09015400	86.57867900	26.13153100
O	14.79003600	83.44464500	25.67355800
O	14.84078000	81.85083200	24.08809300
N	15.49463300	85.43574200	23.87221300
C	15.39871500	86.39602400	22.73244100
C	16.87313500	85.41750400	24.43796800
C	15.15236200	84.07601500	23.38591500
C	15.53158300	87.87446900	23.17376000
C	16.99185700	86.28332900	25.67248600
C	14.91759100	83.02944100	24.49652600
H	14.43132200	86.24243100	22.26451400
H	17.10205500	84.40050400	24.75202200
H	17.59982600	85.72242900	23.68783800
H	16.18237200	86.15217400	22.00997800
H	14.23868800	84.14181100	22.79884600
H	15.94356200	83.72408700	22.71957000
Ni	14.31044000	86.03288200	25.34825300
H	10.03117000	83.97792500	23.41576200
H	14.77891700	87.90062100	27.80022300

$Zn^{2+}[CM\text{-}Asp]^{3-}[HGATH]$ Phyre[2]

C	8.84499700	82.10404800	17.88318000
C	9.51051300	83.25269500	18.60320300
O	9.96513500	84.22266600	17.99241800
H	7.76858300	82.09831700	18.05203900
H	9.06907300	82.25104000	16.80202100
H	9.26998600	81.12995000	18.21286900
N	9.59185900	83.16990200	19.95145800
C	10.22727800	84.21722300	20.73673200
C	11.75103700	83.98859800	20.77256200
O	12.31729000	83.43822200	21.70723500
C	9.63070600	84.32091000	22.14445900
C	10.32182300	85.39728200	22.90488300
N	10.33050100	86.72027100	22.50024400
C	11.24205300	85.33496800	23.91006500
C	11.24139800	87.39097400	23.23655400
N	11.81698200	86.57456400	24.10074800
H	9.29594000	82.32555800	20.41581400
H	10.04515500	85.14821300	20.20122800
H	9.76527800	83.38109400	22.67887800
H	8.55871700	84.50509000	22.04977000
H	11.55520600	84.47276700	24.46858100
H	11.47222700	88.43293000	23.08874500
H	9.75151000	87.12887900	21.78378300
N	12.39169400	84.43567100	19.66531300
C	13.77911100	84.10855900	19.42908700
C	14.76676900	85.26349700	19.33083000
O	15.96199900	85.00065100	19.26025500
H	11.79679800	84.66921000	18.87677700
H	13.87744800	83.55703600	18.49129100
H	14.11919400	83.45637800	20.23027500
N	14.27930600	86.52267000	19.26971900
C	15.13932900	87.60091800	18.78141700
C	14.60261000	89.00197900	19.09500400
O	14.69817900	89.88898000	18.25223100
C	15.41938900	87.44644000	17.28926600
H	13.27777300	86.63749600	19.22928400
H	16.07388500	87.51448500	19.34080500
H	16.13037400	88.19833200	16.95452400
H	15.83784100	86.45965500	17.10143900
H	14.50122400	87.56023600	16.71227200
N	14.09664400	89.22042900	20.33111100
C	13.82098500	90.58410500	20.75215500
C	14.82862100	91.14100800	21.76158300
O	15.15464200	92.32578300	21.71822700
C	12.39358200	90.82456300	21.28902000
O	12.18319500	90.25362600	22.58285900
C	11.33348200	90.25956800	20.36754600
H	14.13682000	88.48029500	21.02761200
H	13.92534300	91.19820000	19.86239000
H	12.28028900	91.91145600	21.35850100
H	12.84933100	90.58373700	23.20045200
H	10.34169100	90.48298400	20.76002700
H	11.42274500	90.69982600	19.37421300
H	11.44155800	89.17889400	20.27568900
N	15.22739900	90.29541100	22.72755200
C	16.06542500	90.73065800	23.82346900
C	17.56840000	90.71691300	23.51236200
O	18.31453100	91.52448000	24.06889600
C	15.80494600	89.85197900	25.05980600
C	14.37421100	89.83375500	25.49204500
N	13.58586400	88.68900800	25.48730800
C	13.61430100	90.87257800	25.95906500
C	12.39620800	89.03643000	25.95079100
N	12.37564900	90.34690300	26.24422800
H	14.95959700	89.30606400	22.69301100
H	15.82114400	91.76842400	24.04274700
H	16.43174300	90.23081200	25.86836000
H	16.11885600	88.82909300	24.86677900
H	13.84636300	91.91005800	26.11795000
H	11.55218600	88.38073500	26.06425900
H	11.58509600	90.85416200	26.60861400
N	17.99467700	89.76429200	22.67517100
C	19.40245500	89.55682900	22.39743100
H	17.33981500	89.08088600	22.29771600
H	19.99178000	89.68648400	23.30419800
H	19.52917300	88.51595700	22.02618000
H	19.78631400	90.26617500	21.63031900
O	14.59226700	87.53980900	22.71569300
O	16.61363400	87.37625400	21.78499200
O	15.96049400	86.78681700	25.39663300
O	17.85089400	85.75119000	25.96453300
O	13.57209800	85.19297400	26.13435200
O	13.89325400	83.01389700	26.50536900
N	14.49910700	85.03332300	23.53681300
C	15.76637100	85.43855100	22.89841100
C	16.98351200	85.28448100	23.80870700
C	14.46171800	83.81326300	24.34974000

C	15.65874000	86.90308300	22.42151400
C	16.92489500	85.98524800	25.17166300
C	13.93867500	84.02426800	25.78820800
H	15.95507000	84.84596300	21.99766500
H	17.22421200	84.23478100	23.97282300
H	17.83657000	85.70340100	23.27339400
H	13.78499300	84.96048000	22.82199700
H	13.80959400	83.07778300	23.87785400
H	15.44229500	83.35055100	24.42934000
Zn	13.91563100	86.72693100	24.76860300

Zn^{2+}[CM-Asp]$^{3-}$[HGATH] SAS $Phyre^2$

C	8.84499700	82.10404800	17.88318000
C	9.50421000	83.25099300	18.61364300
O	9.92771900	84.24118400	18.02566800
H	7.76727800	82.09543300	18.04526300
H	9.06907300	82.25104000	16.80202100
H	9.26998600	81.12995000	18.21286900
N	9.60948100	83.13295300	19.96409600
C	10.26619200	84.16122100	20.75622800
C	11.79383900	83.96777700	20.69976500
O	12.40139500	83.32755800	21.53992500
C	9.76379800	84.18246300	22.20317400
C	10.50198300	85.21570600	22.97907200
N	10.51130100	86.55795200	22.63021100
C	11.46847500	85.09831900	23.93522800
C	11.46500700	87.18307000	23.36282600
N	12.06309800	86.32121800	24.15823400
H	9.36593700	82.26273100	20.40881400
H	10.02702000	85.10875800	20.27353000
H	9.94872000	83.21550600	22.67133100
H	8.68419000	84.35286000	22.19472100
H	11.80214500	84.21093200	24.44146300
H	11.69444400	88.23253100	23.26611000
H	9.89439500	87.01015700	21.97631600
N	12.38084000	84.56438500	19.62662500
C	13.77017500	84.30144900	19.31474500
C	14.68787700	85.50460400	19.12962500
O	15.85805700	85.29821700	18.85207500
H	11.74653900	84.80892200	18.87251500
H	13.85341600	83.71962500	18.39390300
H	14.18309000	83.69403900	20.11778300
N	14.15113000	86.74080700	19.24712900
C	14.91718900	87.91678400	18.82807100
C	14.28614100	89.23668600	19.29524400
O	14.15978400	90.16472300	18.50842600
C	15.13013700	87.92786800	17.31843400
H	13.16625900	86.82030400	19.44085400
H	15.88137200	87.84363600	19.33892900
H	15.75240200	88.77372500	17.03377000
H	15.62193600	87.00539700	17.01752400
H	14.17791200	88.02277900	16.79575000
N	13.94815100	89.33297200	20.60747900
C	13.68476700	90.64943600	21.16471400
C	14.73075700	91.14185300	22.17292000
O	14.97099400	92.33974200	22.25660000
C	12.29190100	90.82781100	21.80326800
O	12.16642000	90.15899000	23.06165700
C	11.17813100	90.32130100	20.91170400
H	14.16311800	88.56133000	21.23944700
H	13.73309300	91.34233800	20.32877500
H	12.17535000	91.90509700	21.96405300
H	12.94806200	90.33686300	23.60309700
H	10.21110100	90.49583600	21.38386100
H	11.19858000	90.83247000	19.94920700
H	11.29826700	89.25264100	20.72924800
N	15.24085300	90.21354900	23.01016800
C	16.14421400	90.57757100	24.08050700
C	17.63130100	90.59775700	23.69774400
O	18.40798100	91.29776200	24.33955000
C	15.98149500	89.61349800	25.27110300
C	14.57574000	89.47730900	25.75457000
N	13.85199600	88.29510600	25.66681900
C	13.77950300	90.42019100	26.34788300
C	12.66845400	88.51990400	26.20420300
N	12.58339500	89.79506700	26.62876700
H	15.03120600	89.21906000	22.87311900
H	15.90671500	91.59723800	24.37873900
H	16.61670300	89.98826800	26.07429000
H	16.34940100	88.62144700	25.01435700
H	13.96083800	91.45000100	26.59743700
H	11.87280900	87.80077000	26.27618200
H	11.77955200	90.21794900	27.06089300
N	18.00613500	89.77213800	22.70608700
C	19.40245500	89.55682900	22.39743100
H	17.33420500	89.13956200	22.27507300
H	20.00055500	89.69223700	23.29749400
H	19.52917300	88.51595700	22.02618000
H	19.78631400	90.26617500	21.63031900
O	14.66558700	87.46258800	22.68791900
O	16.61173600	87.49681000	21.59646800
O	16.31082400	86.57220600	25.16121700
O	18.27413100	85.55379300	25.48173500
O	13.89913800	84.77554700	25.90942900
O	14.02032800	82.54018600	25.94128500
N	14.77214600	84.90022800	23.29519200
C	15.95363700	85.42227400	22.58304700
C	17.25480800	85.25810500	23.36643400
C	14.84483000	83.61565900	24.00009900
C	15.73904400	86.91549700	22.25661900
C	17.28749600	85.82334200	24.79872300
C	14.19497200	83.63690700	25.40948200
H	16.08791000	84.92666100	21.61490400
H	17.56872100	84.21534700	23.40365800
H	18.02605100	85.78533200	22.80392600
H	13.98491500	84.87745300	22.65928000
H	14.36371100	82.82438500	23.42520400
H	15.88119700	83.32364800	24.15159400
Zn	14.28960700	86.41284600	24.77097700

Co^{2+}[CM-Asp]$^{3-}$[HGATH] $Phyre^2$

C	8.84540900	82.10413200	17.88322700
C	9.48217200	83.26262600	18.61105100
O	9.81153300	84.29543000	18.02399600
H	7.76693100	82.09364100	18.04133600
H	9.06888600	82.25106400	16.80198500
H	9.26982200	81.12989100	18.21289300
N	9.67047800	83.11913800	19.94348500
C	10.25724600	84.18319400	20.74158700
C	11.79488600	84.09335500	20.68247800
O	12.46544800	83.53292400	21.53974100
C	9.73428200	84.18387400	22.18171400
C	10.41011800	85.26605000	22.94683100
N	10.35283000	86.59851400	22.58079900
C	11.39860000	85.20699600	23.88744200
C	11.29472600	87.27456100	23.28032900
N	11.95317200	86.45401400	24.07600800
H	9.47095100	82.23363600	20.38156400
H	9.96589800	85.11505500	20.25907500
H	9.95528500	83.23205500	22.66393100
H	8.64945300	84.30376000	22.15284600
H	11.76775100	84.33430000	24.39580800
H	11.47726000	88.33112200	23.17570900
H	9.71645700	87.00864200	21.91591300
N	12.32345800	84.67811800	19.58038700
C	13.71387300	84.49581500	19.23623600
C	14.58081000	85.74234400	19.12471600
O	15.76358400	85.59682500	18.84174600
H	11.66286900	84.90986600	18.84572000
H	13.79783100	83.99579600	18.26912400
H	14.16688600	83.84318300	19.97971900
N	14.00102300	86.95467000	19.29578500
C	14.68881600	88.13556300	18.77274900
C	14.19297300	89.47018200	19.33995100
O	14.16093200	90.45424300	18.60767100
C	14.65902500	88.15081700	17.24596400
H	12.99631500	86.97724300	19.39196300
H	15.72254800	88.04770900	19.11429400
H	15.25463500	88.97409000	16.85938300
H	15.06689900	87.21498500	16.86865500
H	13.63676900	88.26495300	16.88367400
N	13.88089900	89.53489700	20.65600800
C	13.66831300	90.83624400	21.27040700
C	14.77194200	91.26957700	22.23919700
O	15.09529800	92.45364200	22.30948400
C	12.30194400	91.00968000	21.96658100
O	12.18931800	90.23632900	23.16262600
C	11.14865200	90.61810200	21.06655200
H	13.98302400	88.71146100	21.23779200
H	13.69429500	91.55470500	20.45651000
H	12.22751100	92.07282000	22.21840800
H	12.91174400	90.45243000	23.76732200
H	10.20233500	90.78971100	21.57900400
H	11.16328500	91.21227200	20.15281700
H	11.21447700	89.56471800	20.79385000
N	15.25709400	90.31664500	23.05275600
C	16.17877300	90.61812100	24.12500700
C	17.65441600	90.62909400	23.70935500
O	18.45212600	91.34163400	24.31967900
C	16.01693600	89.59442100	25.26654400
C	14.62114300	89.45710900	25.77964800
N	13.85782100	88.30550400	25.61782700
C	13.88151800	90.36157500	26.49400900
C	12.70462100	88.51561800	26.22999500
N	12.68252500	89.74793300	26.76871400
H	14.98172100	89.33759800	22.93110100
H	15.96173800	91.62183800	24.48558100
H	16.67738600	89.90905800	26.07545100
H	16.35847800	88.61444500	24.94038200
H	14.10826300	91.35592300	26.83343900
H	11.88853400	87.81922500	26.27449100
H	11.91514400	90.14867800	27.28327500
N	18.01106900	89.78293800	22.73177500
C	19.40217300	89.55671400	22.39710000
H	17.31621500	89.18254200	22.29660200
H	20.01289000	89.67965500	23.29016000
H	19.52927400	88.51593000	22.02630700
H	19.78644100	90.26630400	21.63049800
O	14.67952600	87.59914500	22.87860600
O	16.47500900	87.51601900	21.56533200
O	16.46807300	86.53254100	25.07613700
O	18.43967900	85.47847100	25.17046200
O	13.89416400	85.34993200	26.02827000
O	14.07212200	83.17218000	26.49517200
N	14.60732800	85.06287900	23.44599300
C	15.76827600	85.46778600	22.59860800
C	17.13194400	85.17635900	23.21263600
C	14.71834400	83.87355800	24.31685000
C	15.65323400	86.97565700	22.30576400
C	17.36746300	85.77398900	24.60854500
C	14.18030600	84.13847000	25.73298400
H	15.71877400	84.95495700	21.63546000
H	17.33469200	84.10604900	23.24075900
H	17.87880300	85.60350400	22.54224200
H	13.81711400	84.90970300	22.82469600
H	14.18024800	83.03185900	23.88219400
H	15.75417400	83.56652700	24.42647900
Co	14.19702700	86.63648600	24.56647500

Co^{2+}[CM-Asp]$^{3-}$[HGATH] SAS $Phyre^2$

C	8.84540900	82.10411800	17.88321700
C	9.48721300	83.26403100	18.60715400
O	9.85838400	84.27294900	18.01441300
H	7.76677900	82.09223300	18.04063900
H	9.06888600	82.25106300	16.80198500
H	9.26981800	81.12988600	18.21289900
N	9.63132700	83.14410500	19.95287900
C	10.21998800	84.21462000	20.74307400
C	11.75837700	84.12463700	20.68989400
O	12.41767100	83.57241000	21.55496500
C	9.70362900	84.21983400	22.18452900
C	10.36197900	85.31567900	22.94537700
N	10.29696200	86.64579300	22.55988300
C	11.33372000	85.28002200	23.90419000
C	11.21783300	87.34025700	23.27999400
N	11.86604900	86.53530300	24.09347700
H	9.41179700	82.27117500	20.40443500
H	9.92595500	85.14194500	20.25129000
H	9.95081100	83.27565600	22.67048400
H	8.61466600	84.31317300	22.16254800
H	11.70557300	84.42170200	24.43550100
H	11.39238100	88.39909900	23.17385800
H	9.65106000	87.04494600	21.89959100
N	12.29432100	84.70239700	19.58191900
C	13.68619400	84.49136900	19.24590000
C	14.57669800	85.72135600	19.10552100
O	15.74473800	85.55151100	18.80078500
H	11.63363300	84.89273300	18.83412200
H	13.76784700	83.95895000	18.29602700
H	14.12533300	83.85573300	20.01241800
N	14.01934400	86.94336100	19.29243300
C	14.74943900	88.13652900	18.85635600
C	14.15965400	89.44664000	19.40028900
O	13.98475200	90.39366500	18.64649600
C	14.86057300	88.19020000	17.33626300
H	13.02475900	86.99107800	19.44660900
H	15.74553600	88.04604400	19.29669100
H	15.46267500	89.04371700	17.03217000
H	15.33057100	87.27683800	16.97758500
H	13.87551300	88.29818500	16.88126400
N	13.92220000	89.52101900	20.73568900
C	13.71413600	90.82773100	21.34090600
C	14.79746400	91.25696600	22.33811500
O	15.09713100	92.44034400	22.43057800
C	12.34131500	91.02365500	22.01369800
O	12.19601700	90.25346800	23.20773200
C	11.19344000	90.65507100	21.09699000
H	14.13948100	88.72631000	21.32857900
H	13.76560900	91.54441500	20.52576200
H	12.28367700	92.08883900	22.26518200
H	12.99762400	90.32376600	23.74463000
H	10.24206600	90.82603000	21.60129000
H	11.22450800	91.25499500	20.18763700
H	11.25939500	89.60421000	20.81242700
N	15.27473000	90.29510000	23.15567400
C	16.22153300	90.59401000	24.20750700
C	17.69096900	90.59799200	23.76711000
O	18.50675200	91.26462100	24.39435100
C	16.08879400	89.57747400	25.35966100
C	14.70270400	89.41501500	25.88816700
N	13.93261400	88.27961500	25.66614000
C	13.97859600	90.27199300	26.67273300
C	12.79351800	88.44467200	26.30890100
N	12.78039800	89.64176500	26.93038500
H	15.03692900	89.30997900	23.00208800
H	16.01781200	91.60295100	24.56162900
H	16.74899200	89.91905800	26.15730000
H	16.44846000	88.59654400	25.04938900
H	14.21523200	91.24185500	27.07135800
H	11.98925100	87.73285300	26.32379800
H	12.02231200	90.00878600	27.47915000
N	18.01767000	89.78833400	22.74143000
C	19.40218800	89.55671300	22.39711700
H	17.31912800	89.18912300	22.31243100
H	20.01631200	89.68678300	23.28722000
H	19.52926400	88.51592600	22.02629500
H	19.78643500	90.26629200	21.63048800
O	14.69726600	87.59600600	22.87278800
O	16.48656000	87.50700600	21.55139000
O	16.47212800	86.56051600	25.02485000
O	18.37183400	85.37815300	25.20559200
O	13.81690900	85.36789900	25.98261600
O	13.75762700	83.16416500	26.38038000
N	14.61374400	85.06472800	23.43869100
C	15.77122900	85.45752800	22.58129800
C	17.12952000	85.15254300	23.19896800
C	14.71434700	83.88421300	24.32316300
C	15.67115900	86.96767000	22.29566400
C	17.35060200	85.73947700	24.61098100
C	14.02896800	84.13786300	25.68260000
H	15.71283900	84.95198200	21.61333400
H	17.32776500	84.08087600	23.21843800
H	17.88078800	85.59181500	22.54166800
H	13.81062400	84.92244300	22.83308400
H	14.27960800	83.00299600	23.85207200
H	15.75589600	83.66813800	24.54282200
Co	14.26365200	86.63463800	24.57768400

Cu^{2+}[CM-Asp]$^{3-}$[HGATH] SAS $Phyre^2$

C	8.84541800	82.10412300	17.88322500
C	9.48132700	83.27065600	18.60906700
O	9.80560400	84.30028500	18.02196500
H	7.76642100	82.09336000	18.03820200
H	9.06887900	82.25106300	16.80198300
H	9.26981800	81.12988500	18.21289500
N	9.67686700	83.13027000	19.94306700
C	10.15069400	84.21976800	20.79797900
C	11.69280400	84.26431800	20.77604100
O	12.36812200	83.95895400	21.74565700
C	9.59889300	84.03069800	22.21383300
C	9.66983400	85.23147100	23.08746600
N	8.95280600	86.39040600	22.84427300
C	10.30800400	85.45671400	24.27598500
C	9.18786800	87.24539400	23.87508600
N	9.99473500	86.70985600	24.75842500
H	9.44382300	82.25574300	20.38509400
H	9.78001900	85.14630600	20.35569800
H	10.15235200	83.22966900	22.70404700
H	8.55790400	83.70592100	22.11653900
H	10.99032900	84.80702200	24.79780300
H	8.75990900	88.23322900	23.91770400
H	8.35650100	86.57000200	22.05461100
N	12.22068200	84.60579800	19.57247800
C	13.59079500	84.25684300	19.24816000
C	14.61580100	85.37919400	19.11173500
O	15.78631500	85.08043700	18.93615600
H	11.55123800	84.70868400	18.81362000
H	13.60778800	83.72047000	18.29745600
H	13.96871100	83.57953300	20.01197500
N	14.15887700	86.64707900	19.16079300
C	15.02735600	87.78461500	18.85484300
C	14.43802300	89.09315600	19.39654700
O	14.55208300	90.14174500	18.77997100
C	15.32121100	87.88425700	17.36322200
H	13.16693100	86.79024600	19.26636900
H	15.96040100	87.61324800	19.39982900
H	15.99149000	88.71918200	17.17028700
H	15.78929500	86.96117200	17.02564400
H	14.40281200	88.05161200	16.79866300
N	13.80513300	88.99036400	20.58655100
C	13.26023000	90.13694900	21.27382800
C	14.16970200	90.77092300	22.34167400
O	13.92340800	91.90949600	22.72242400
C	11.89710700	89.76710500	21.89839700
O	11.97673200	88.53440400	22.60745600
C	10.82134100	89.58827400	20.84494000
H	13.88666300	84.12953100	21.11438700
H	13.11000900	90.92277700	20.53641300
H	11.61958800	90.58163400	22.57472100
H	12.57441300	88.61302900	23.36182800
H	9.88439100	89.29566800	21.31881000
H	10.66149100	90.51597000	20.29498700
H	11.10704200	88.80849900	20.13752700
N	15.16576100	89.99903100	22.81769700
C	16.00739900	90.41414000	23.91905100
C	17.49793000	90.55173800	23.57575600
O	18.19521600	91.34081500	24.20541000

C	15.90217900	89.42676100	25.10662000
C	14.50389300	89.19321200	25.56576500
N	13.78630800	88.03989000	25.26429200
C	13.69851900	90.00179600	26.31528500
C	12.59189500	88.14817700	25.82587700
N	12.50361200	89.32906700	26.46441200
H	15.17996000	89.00735300	22.56630700
H	15.66993200	91.40105900	24.22544400
H	16.49437200	89.84147200	25.92297700
H	16.34669400	88.46572400	24.84725600
H	13.86909200	90.97333400	26.74171500
H	11.77917000	87.44102600	25.74857200
H	11.69220800	89.65791300	26.95946100
N	17.98034400	89.71625900	22.63934100
C	19.40216100	89.55669900	22.39707100
H	17.38334800	89.01176800	22.21809600
H	19.96306900	89.70506700	23.31801200
H	19.52927800	88.51592000	22.02631800
H	19.78644500	90.26630900	21.63050800
O	15.02542300	87.25470600	22.57667200
O	16.99956800	87.11429500	21.55757900
O	16.47324100	86.29905100	25.06838600
O	18.20317900	84.96616100	25.58263800
O	13.33289100	85.09390600	25.37779300
O	13.00572000	82.89784800	25.62685200
N	14.81258600	84.77228600	23.19026400
C	16.10010200	85.10556000	22.53312400
C	17.31897500	84.78815500	23.39243300
C	14.66528500	83.59610000	24.05459400
C	16.07028100	86.60770800	22.18090400
C	17.33533000	85.39578000	24.81656100
C	13.56427500	83.85330700	25.10437200
H	16.19232000	84.57085300	21.58278200
H	17.47118500	83.71139700	23.47111900
H	18.18793000	85.18391700	22.86480200
H	14.06638300	84.73729000	22.49834400
H	14.42222900	82.69755500	23.48713400
H	15.58974300	83.42221000	24.60170000
Cu	14.38671800	86.37257900	24.32060700

Ni^{2+}[CM-Asp]$^{3-}$[HGATH] Phyre^2

C	8.84509100	82.10405300	17.88316700
C	9.48834700	83.26689100	18.60606900
O	9.87154500	84.27306700	18.00222200
H	7.76754500	82.09536500	18.04618800
H	9.06902400	82.25104700	16.80201100
H	9.26994900	81.12994100	18.21289200
N	9.62610100	83.16141300	19.94526900
C	10.07748500	84.26809300	20.78716600
C	11.61722700	84.27624300	20.87426300
O	12.22013500	84.04836400	21.91777400
C	9.41089700	84.16144500	22.16137300
C	9.50319500	85.38214000	23.00655300
N	8.86638600	86.56859600	22.69882700
C	10.09860400	85.61166300	24.21780000
C	9.09626000	87.44247800	23.71106600
N	9.83532100	86.89430400	24.65060900
H	9.34609200	82.30673300	20.40064500
H	9.77202100	85.18691600	20.28433500
H	9.85480200	83.33216500	22.71142600
H	8.35967000	83.91575100	21.99014100
H	10.70495200	84.93319000	24.79302200
H	8.71650900	88.45051400	23.70355300
H	8.31194800	86.75456300	21.87867600
N	12.23254800	84.50624000	19.69256800
C	13.61517500	84.13402200	19.48410400
C	14.64810900	85.24176800	19.32622400
O	15.83071900	84.92874400	19.25527800
H	11.62229200	84.58740400	18.88312900
H	13.68800700	83.52763800	18.57963300
H	13.94166900	83.51462000	20.31763900
N	14.20638000	86.51554300	19.25337600
C	15.10881400	87.58492700	18.83564700
C	14.70330400	88.95916300	19.37028100
O	15.15316700	89.97879900	18.85637200
C	15.27018100	87.61600500	17.31646800
H	13.21019200	86.65188800	19.15821100
H	16.07139400	87.35870600	19.29746800
H	15.98483600	88.38304400	17.02780400
H	15.63056000	86.64809100	16.97126100
H	14.31543100	87.82928300	16.83303200
N	13.85692900	88.98673700	20.41542900
C	13.36410700	90.22344900	20.97584700
C	14.29029600	90.90397000	21.99576100
O	14.16947900	92.11015800	22.20088000
C	11.97010600	89.96427000	21.59666400
O	11.98029700	88.76395300	22.36788000
C	10.90236200	89.79259600	20.53354400
H	13.59504800	88.13118900	20.88333400
H	13.25771600	90.94855000	20.17115700
H	11.72603400	90.82013700	22.23136300
H	12.51185900	88.87572300	23.16665900
H	9.94124200	89.59010500	21.00566600
H	10.81314300	90.69744200	19.93240700
H	11.14639500	88.95777500	19.87481200
N	15.15306400	90.10263400	22.63896400
C	15.96553400	90.54249400	23.75354600
C	17.46637500	90.64541500	23.44605500
O	18.14779300	91.50881200	24.00042600
C	15.78824200	89.59248500	24.96354000
C	14.36672900	89.45784300	25.39196800
N	13.57348400	88.34513300	25.12000600
C	13.60250700	90.35353500	26.08429500
C	12.37276600	88.57142700	25.64391400
N	12.36105000	89.77967100	26.26222400
H	15.08342100	89.09343100	22.49610600
H	15.64115800	91.54468200	24.02279900
H	16.37931900	89.99927200	25.78480300
H	16.18988000	88.60680500	24.73309300
H	13.83570400	91.32444000	26.48165700
H	11.51799500	87.91289800	25.57985100
H	11.56705800	90.19288100	26.68856700
N	17.97505300	89.71512600	22.62852900
C	19.40243400	89.55681700	22.39741000
H	17.37668300	88.98432800	22.25622100
H	19.96218500	89.69547800	23.32047800
H	19.52918600	88.51595500	22.02619100
H	19.78631600	90.26618600	21.63032900
O	14.92710200	87.38800000	22.83822300
O	16.83051300	87.07575300	21.74355200
O	16.78584600	86.42508700	25.07616300
O	18.02041800	84.63937200	25.65477500
O	13.21266600	85.67054900	25.67330800
O	12.87961900	83.58211400	26.37818400
N	14.49720100	84.98069300	23.50998000
C	15.78664400	85.15523500	22.76617200
C	17.02383100	84.65170700	23.49482200
C	14.36568500	83.92215600	24.52994200
C	15.90247500	86.64235500	22.41633800
C	17.29698600	85.29389300	24.87119500
C	13.39659300	84.38835800	25.61491900
H	15.71908400	84.61816700	21.81816800
H	17.00087400	83.56645100	23.58776400
H	17.87582400	84.88620000	22.85276000
H	13.75579900	84.81536900	22.82339800
H	14.02776200	82.98485800	24.09023900
H	15.32115500	83.74297900	25.01687600
Ni	14.04300800	86.64746800	24.31607200

Ni^{2+}[CM-Asp]$^{3-}$[HGATH] SAS Phyre^2

C	8.84508400	82.10405000	17.88316700
C	9.46846400	83.26799800	18.62153900
O	9.76901700	84.31485600	18.05320100
H	7.76528600	82.08888000	18.03331200
H	9.06902900	82.25104700	16.80201200
H	9.26994800	81.12994000	18.21288900
N	9.67675700	83.10394500	19.95164600
C	10.13394400	84.18542500	20.82373100
C	11.67379300	84.26088300	20.78938000
O	12.36469200	83.92160200	21.73818700
C	9.59644200	83.96248900	22.23935000
C	9.67940300	85.14772000	23.13267800
N	8.97841400	86.31851500	22.90093700
C	10.32277300	85.35428200	24.32197200
C	9.22702300	87.16141900	23.93766700
N	10.02725600	86.60696600	24.81552800
H	9.46374600	82.21631700	20.37730000
H	9.74186400	85.11236000	20.40127900
H	10.15398600	83.15046700	22.70612400
H	8.55454600	83.63999000	22.14570900
H	10.99042000	84.68782000	24.84160300
H	8.81612300	88.15589200	23.98796400
H	8.38569700	86.51456200	22.11245900
N	12.17942100	84.66934300	19.59848600
C	13.55046400	84.36313100	19.23748700
C	14.54186900	85.51270400	19.08413800
O	15.70533100	85.24224100	18.83589800
H	11.49639300	84.78228500	18.85260000
H	13.56030000	83.83430000	18.28253800
H	13.96414200	83.68954700	19.98590600
N	14.07010900	86.77232600	19.20796400
C	14.90673200	87.92266300	18.86069800
C	14.36747500	89.22551900	19.46217500
O	14.51665400	90.29092400	18.88324400
C	15.09170900	88.04919400	17.35265800
H	13.07683700	86.89565400	19.32872800
H	15.87589600	87.74332700	19.33408300
H	15.73998100	88.89278800	17.12682400
H	15.54010400	87.13585400	16.96578500
H	14.13338000	88.21801100	16.85878900
N	13.74222500	89.11005500	20.65513500
C	13.22739700	90.25428000	21.37041200
C	14.15651500	90.84832300	22.44558300
O	13.90894100	91.96613600	22.88262700
C	11.85357200	89.90392300	21.98423300
O	11.88361200	88.62736700	22.61690900
C	10.76657700	89.83355200	20.92928900
H	13.76263300	88.22658700	21.14631200
H	13.09572000	91.05813700	20.64878800
H	11.61429800	90.68677800	22.71011300
H	12.48765800	88.63162600	23.37095100
H	9.82126500	89.54420600	21.38863200
H	10.63766900	90.80098300	20.44344800
H	11.02062700	89.09331000	20.16899200
N	15.17500000	90.06786100	22.85006300
C	16.03334600	90.41145900	23.96332800
C	17.51982700	90.54882600	23.60662300
O	18.22968800	91.32317100	24.24078400
C	15.92766200	89.35241800	25.08864000
C	14.53011400	89.12866700	25.55278200
N	13.75775200	88.02579500	25.19509900
C	13.77535700	89.91361400	26.37632800
C	12.58283900	88.14127400	25.79880900
N	12.55937500	89.27951700	26.51450000
H	15.16245800	89.08499700	22.57755100
H	15.71223600	91.38248700	24.33170500
H	16.53404300	89.70891800	25.92216700
H	16.35626900	88.40224200	24.76855000
H	13.99798400	90.84603700	26.86172700
H	11.74845100	87.45991500	25.71082300
H	11.77605400	89.60375700	27.05522400
N	17.98460000	89.72613600	22.65015000
C	19.40245300	89.55682800	22.39743400
H	17.37469500	89.03187200	22.23070400
H	19.96900000	89.70199200	23.31536700
H	19.52917200	88.51595700	22.02617900
H	19.78631400	90.26617600	21.63031900
O	14.97508500	87.33562800	22.71398500
O	16.83844400	87.20761200	21.51364200
O	16.89228500	86.33018900	24.77690200
O	18.26175500	84.59762200	25.23666100
O	13.45156200	85.31896300	25.46118200
O	13.17154500	83.15659200	25.94507700
N	14.71510400	84.86595900	23.22969300
C	15.95404500	85.16462100	22.44062400
C	17.24556400	84.69121300	23.08899300
C	14.68687100	83.73581200	24.17939800
C	15.97538500	86.67745400	22.19900700
C	17.49507000	85.25496700	24.51316000
C	13.67669500	84.05057700	25.28667400
H	15.86980400	84.69652200	21.45608800
H	17.30070200	83.60238200	23.10401700
H	18.05770900	85.04023000	22.44803700
H	13.94309500	84.71774800	22.57814700
H	14.42900100	82.80037100	23.68356100
H	15.65879600	83.62659800	24.65526100
Ni	14.24276700	86.43567900	24.20026800

Ni^{2+}[NTA]$^{3-}$[HGATH] Phyre^2

C	8.84500000	82.10400000	17.88300000
C	9.46663900	83.26522800	18.62335800
O	9.61358800	84.37054600	18.10041500
H	7.76503300	82.09526700	18.03101400
H	9.06900000	82.25100000	16.80200000
H	9.27000000	81.13000000	18.21300000
N	9.85014300	83.03135000	19.90113300
C	10.48311300	84.06009600	20.71055700
C	11.99408800	84.08821800	20.41388000
O	12.79787100	83.42754100	21.05402800
C	10.19543300	83.84302000	22.20040600
C	10.96658300	84.79459600	23.04648300
N	11.01525800	86.15627800	22.80665900
C	11.87083000	84.57882000	24.04187900
C	11.92922000	86.71455300	23.61832400
N	12.46588900	85.77976600	24.38834600
H	9.80674200	82.09318900	20.26615800
H	10.05261700	85.00720800	20.38695800
H	10.48327000	82.83255600	22.49000800
H	9.11816900	83.93859500	22.35332500
H	12.16111300	83.65353700	24.50050000
H	12.18485000	87.75655000	23.57793700
H	10.46251600	86.66988900	22.13960600
N	12.32988600	84.89272300	19.37210200
C	13.66700300	84.87570800	18.82112700
C	14.36338400	86.21566900	18.62411000
O	15.49973100	86.21288500	18.16203000
H	11.56033700	85.16545900	18.77089400
H	13.66898400	84.39968500	17.83743900
H	14.29177300	84.26920700	19.47252200
N	13.68942300	87.34782000	18.92049400
C	14.15865100	88.62535200	18.39091800
C	13.72856100	89.84662600	19.21350700
O	13.50459800	90.90926800	18.63810100
C	13.76711200	88.77940600	16.92318300
H	12.72935600	87.26374000	19.21845700
H	15.24820400	88.59320500	18.46146400
H	14.18825900	89.69143700	16.50721700
H	14.14450200	87.92708300	16.35977100
H	12.68230700	88.82007800	16.81749900
N	13.68010300	89.70845200	20.55355000
C	13.41745600	90.86466300	21.39177000
C	14.51582700	91.19563600	22.40789800
O	14.66968100	92.36185600	22.77125000
C	12.06477700	90.80056600	22.14285000
O	12.07479000	89.84043700	23.20336100
C	10.91514000	90.44232500	21.22574400
H	13.91534800	88.81301300	20.98800700
H	13.36087900	91.71950200	20.72303400
H	11.90538300	91.79739600	22.56520900
H	12.71525800	90.11182000	23.87491100
H	9.97541000	90.48261600	21.77610500
H	10.86218900	91.13971100	20.38984700
H	11.04438600	89.43523300	20.82815600
N	15.18464800	90.15523500	22.92788800
C	16.05990600	90.28545100	24.07458500
C	17.55699800	90.41917500	23.75338000
O	18.27588400	91.09101000	24.49531800
C	15.85407500	89.06548900	24.99022000
C	14.49325100	89.02686100	25.60164000
N	13.73776100	87.87187700	25.73848100
C	13.77709400	90.06323600	26.14011300
C	12.61132900	88.20602100	26.35622300
N	12.60378300	89.52159100	26.61143500
H	14.95533500	89.20057900	22.61596200
H	15.80046300	91.20343800	24.59746800
H	16.61476200	89.07700300	25.77223500
H	16.00617500	88.15821500	24.41311400
H	14.00125000	91.10941700	26.23814000
H	11.80798000	87.53793800	26.60700700
H	11.85637200	90.02614600	27.06113800
N	17.99805500	89.72363100	22.70099700
C	19.40200000	89.55700000	22.39700000
H	17.35278100	89.10630500	22.20212000
H	20.02600000	89.68300000	23.28100000
H	19.54897800	88.54996900	22.00826100
H	19.78600000	90.26600000	21.63000000
O	14.54721100	87.55327800	22.17712700
O	16.67366400	87.55437900	21.48421100
O	15.83395500	86.16361900	26.17302600
O	18.01347500	85.79159700	26.21855400
O	14.23230300	84.07782500	25.66926200
O	15.01998300	82.03783100	25.30978400
N	15.72712200	85.07462600	23.47586400
C	15.67121500	85.46023400	22.06047900
C	16.97623900	85.46512600	24.11692900
C	15.44866000	83.65521100	23.62613600
C	15.63432100	86.97964200	21.88386000
C	16.93712000	85.80246900	25.61677000
C	14.88797600	83.21992700	24.97878200
H	14.75025100	85.04809000	21.65301500
H	17.75825400	84.70813800	23.97680000
H	17.34471100	86.37518600	23.63894500
H	16.51952400	85.04978000	21.49619500
H	14.67690700	83.38042900	22.90341800
H	16.33003000	83.03633500	23.41256700
Ni	14.11666300	85.98626000	25.37049600

Ni^{2+}[NTA]$^{3-}$[HGATH] SAS Phyre^2

C	8.84502400	82.10401000	17.88299400
C	9.45010500	83.27735200	18.62325800
O	9.47863900	84.40436600	18.14445200
H	7.76390400	82.09197700	18.02122100
H	9.06898800	82.25099600	16.80199700
H	9.26999400	81.13000100	18.21301100
N	9.94965500	83.01748100	19.86248400
C	10.58954600	84.05775800	20.65235300
C	12.07813900	84.17126800	20.26620500
O	12.94313100	83.52273200	20.83238900
C	10.41114500	83.79849400	22.15002400
C	11.09276800	84.82807500	22.98236000
N	10.99235200	86.18972500	22.75060500
C	11.98439700	84.70997000	24.00403300
C	11.82409000	86.83906100	23.58525800
N	12.43356500	85.96695300	24.37123100
H	10.02917700	82.06359900	20.17462000
H	10.08706600	84.98416500	20.37376900
H	10.84538300	82.83162300	22.40608200
H	9.33885000	83.74957600	22.35930100
H	12.35826100	83.82570200	24.48320700
H	11.97639600	87.90157900	23.53956700
H	10.45807100	86.64025800	22.02804500
N	12.30678100	85.03050800	19.24926500
C	13.62398600	85.21423000	18.66757400
C	13.77483700	86.58599400	18.00889500
O	14.10054400	86.67198400	16.83112700
H	11.49956000	85.34242300	18.72515600
H	13.82827100	84.47310700	17.89359700

H	14.35702900	85.08633300	19.46239500
N	13.50894500	87.64870700	18.79644900
C	13.53407700	88.99215800	18.23532100
C	13.59512400	90.16381200	19.22078600
O	13.69334300	91.28527100	18.73482800
C	12.30248300	89.21266800	17.34119100
H	13.41965300	87.48676200	19.79876700
H	14.42481600	89.07939200	17.61088900
H	12.35097500	90.19456600	16.87638000
H	12.27677700	88.44605700	16.56956900
H	11.39024500	89.14821600	17.93759000
N	13.44864500	89.95525800	20.55184300
C	13.15314900	91.08237400	21.41186300
C	14.14664700	91.36717300	22.54176100
O	13.91795000	92.31829200	23.28965600
C	11.74511700	90.98840200	22.04429100
O	11.71092400	90.02362400	23.10486000
C	10.67435100	90.60414300	21.04700200
H	13.47926600	89.01453700	20.95889800
H	13.16616600	91.96353500	20.77082500
H	11.53061700	91.97510400	22.46020800
H	12.27849300	90.35208900	23.81480400
H	9.69345000	90.64292800	21.52041600
H	10.68233400	91.28555200	20.19561600
H	10.84174700	89.59357700	20.67361100
N	15.17947600	90.52475400	22.68920500
C	16.03179000	90.56820900	23.86833200
C	17.53617500	90.63524800	23.55699700
O	18.25022100	91.44832200	24.13135500
C	15.74998000	89.35004600	24.76436500
C	14.39013800	89.35913900	25.37520700
N	13.65420100	88.20500400	25.60043800
C	13.66012700	90.42143500	25.83677400
C	12.52705700	88.56007800	26.19531500
N	12.49451700	89.89558100	26.35420500
H	15.25670200	89.69089000	22.10086100
H	15.80924900	91.49456900	24.38879600
H	16.50510700	89.28763000	25.55071100
H	15.85172200	88.45131400	24.16619300
H	13.84999500	91.47804200	25.80468800
H	11.73738000	87.89471200	26.49363300
H	11.73913400	90.41829600	26.76438700
N	17.99777700	89.71771400	22.68905400
C	19.40200600	89.55693100	22.39698100
H	17.35058200	89.05017200	22.28008500
H	20.02599600	89.68303800	23.28099700
H	19.55349300	88.54902700	22.01175700
H	19.78599300	90.26602400	21.63001900
O	13.81589600	87.30130600	21.56864000
O	15.92640900	87.99925100	21.73639100
O	15.80630300	86.60403300	26.09600000
O	18.01639000	86.47351400	26.02630200
O	14.27127900	84.43100900	25.74090500
O	15.20449200	82.42947500	25.51605800
N	15.68204000	85.37914000	23.45916600
C	15.48985500	85.66157500	22.03167300
C	16.91197800	85.93574300	24.00734200
C	15.55275400	83.95713800	23.74090000
C	15.04674900	87.10236900	21.76418200
C	16.92007600	86.33743300	25.49556600
C	15.00261400	83.57298400	25.12034600
H	14.70338900	85.00347500	21.67532500
H	17.76106100	85.25570100	23.85825400
H	17.14615400	86.85151100	23.46507300
H	16.40687000	85.46313500	21.45433500
H	14.83971800	83.52733200	23.03490300
H	16.50242500	83.42301800	23.60567500
Ni	14.09377400	86.29542000	25.35510100

Zn^{2+}[CM-Asp]$^{3-}$[HGATH] AlphaFold

C	15.43198700	0.80103800	-29.77900100
C	16.31679100	-0.43523900	-29.76057700
O	17.53481600	-0.39570800	-29.63175600
H	14.60532800	0.62289400	-29.12341500
N	15.64229800	-1.61269500	-29.87832600
C	16.27666500	-2.91914800	-30.00485600
C	17.00165600	-3.39761800	-28.73100100
O	17.91828600	-4.21432600	-28.79285500
C	17.14585500	-3.05563300	-31.24503400
C	16.31007000	-3.26274600	-32.49339200
N	16.84975000	-2.80463200	-33.63689600
O	15.22356200	-3.83854500	-32.46517100
H	14.65067200	-1.57000200	-30.04704600
H	15.45164100	-3.62435100	-30.12416900
H	17.76204600	-3.94652800	-31.12779800
H	17.81680000	-2.20549900	-31.34703500
H	16.39516300	-3.00409800	-34.51327700
H	17.75739300	-2.37300900	-33.65610900
N	16.48403000	-2.93697700	-27.58552200
C	16.89796100	-3.35699400	-26.25401700
H	15.73330600	-2.26919800	-27.65626500
H	16.15060000	-3.99125700	-25.82503600
C	18.83905300	-12.26302200	-25.94104900
C	18.69342600	-11.31520300	-27.10556900
O	19.62337000	-11.13456600	-27.89768300
N	17.54534200	-10.61635600	-27.23027000
C	17.37063900	-9.69747700	-28.34367500
C	18.30786200	-8.48634200	-28.25699000
O	18.69393800	-7.90866400	-29.26460500
C	15.89634700	-9.26846900	-28.46826000
C	15.64982100	-8.57941100	-29.76574100
C	15.75822600	-9.04194200	-31.04722800
N	15.37258600	-7.23505500	-29.89483900
C	15.32594600	-6.92775000	-31.21104000
N	15.56448600	-8.00768900	-31.93277200
H	16.77896900	-10.79224700	-26.60008500
H	17.66678000	-10.20402700	-29.26111600
H	15.61340400	-8.63046700	-27.62944600
H	15.28365000	-10.16988700	-28.40868700
H	16.00879000	-10.03140100	-31.38418200
H	15.24660900	-6.58353800	-29.13654700
H	15.14915500	-5.93310300	-31.58924100
N	18.69540200	-8.09900200	-27.02308800
C	19.69995500	-7.06985800	-26.88476200
C	21.07668400	-7.48981900	-27.39735400
O	21.88339200	-6.63745400	-27.76964200
H	18.37719400	-8.60713200	-26.21464500
H	19.79516500	-6.80975500	-25.83114200
H	19.41100800	-6.17569000	-27.43477000
N	21.35517400	-8.80218400	-27.38017100
C	22.63579400	-9.32717500	-27.81210400
C	22.78023000	-9.41475700	-29.33369700
O	23.89347300	-9.31824000	-29.84215200
C	22.89640000	-10.69118000	-27.17555200
H	20.62146400	-9.46940800	-27.18605000
H	23.40502700	-8.62214300	-27.50171400
H	23.85778400	-11.07588700	-27.51229800
H	22.91993600	-10.59481500	-26.09005700
H	22.11355900	-11.39827000	-27.44806000
N	21.66131400	-9.61191600	-30.06090200
C	21.73337000	-9.64741100	-31.50731900
C	21.86375900	-8.25276200	-32.13153500
O	22.27825400	-8.14326300	-33.28060000
C	20.57419300	-10.44446200	-32.12292400
C	20.66034200	-11.92422400	-31.76671200
O	19.35641800	-9.88159500	-31.67394300
H	20.75994600	-9.70191300	-29.61261500
H	22.66374200	-10.14385400	-31.78146200
H	20.66992800	-10.32911000	-33.20690100
H	19.83557800	-12.45538500	-32.24129700
H	21.60091900	-12.35879000	-32.11178100
H	20.58614300	-12.06332800	-30.68703800
H	18.68124000	-9.91901800	-32.39092000
N	21.54697600	-7.19789100	-31.34896900
C	21.70589800	-5.83290200	-31.79795100
C	22.85614100	-5.05473600	-31.14669200
O	23.27820200	-4.04962500	-31.72173400
C	20.39014800	-5.03924900	-31.65734100
C	19.35079200	-5.54703600	-32.58717000
C	18.35630300	-6.46633400	-32.41974200
N	19.30309900	-5.20328500	-33.92584200
C	18.30939500	-5.89744000	-34.51397700
N	17.71595000	-6.67175700	-33.62554700
H	21.05866900	-7.38255300	-30.48463400
H	21.97202200	-5.88623600	-32.85178200
H	20.60210000	-3.98777000	-31.85373200
H	20.02356100	-5.11047300	-30.63212700
H	18.09354600	-7.00291200	-31.52582300
H	19.89427600	-4.53005000	-34.38677900
H	18.05187600	-5.83478200	-35.55538100
N	23.33981900	-5.50326700	-29.98346200
C	24.38305700	-4.78805600	-29.26495500
H	22.84694600	-6.22443200	-29.47395100
H	23.96570700	-4.02507300	-28.64153400
H	24.93904400	-5.49246900	-28.64974400
H	25.03327000	-4.34161300	-29.98804100
H	19.76435300	-11.98831500	-25.47929600
H	18.02348500	-12.19023800	-25.22437300
H	18.90648200	-13.26660400	-26.30586300
H	16.00276200	1.66372900	-29.44842800
H	15.07065600	0.96078500	-30.77338400
H	17.84386200	-3.88611200	-26.32349500
H	17.00492600	-2.47651500	-25.65549200
O	14.47000100	-6.94840500	-34.57806400
O	12.72521000	-7.10092200	-35.96897300
O	16.82967300	-7.97600600	-36.11327400
O	16.79494500	-8.36467300	-38.30676000
O	17.39857500	-9.82933300	-33.59447000
O	17.50182600	-12.02996900	-33.98479000
N	14.89513600	-9.62257200	-34.66539200
C	14.12099400	-9.02364400	-35.76916300
C	14.86689500	-9.01493900	-37.10634700
C	15.64626600	-10.85662400	-34.88950100
C	13.72625500	-7.56663600	-35.40236300
C	16.27714800	-8.39944700	-37.17495200
C	16.95622400	-10.92339700	-34.08041600
H	13.18468400	-9.56694000	-35.92420200
H	14.93783900	-10.02613500	-37.51151500
H	14.24462800	-8.46664700	-37.81476600
H	14.27468700	-9.74873800	-33.87461200
H	15.05832800	-11.74914300	-34.66738300
H	15.94095100	-10.92544700	-35.93447000
Zn	16.20721600	-8.04126000	-34.02556800

Zn^{2+}[CM-Asp]$^{3-}$[HGATH] SAS AlphaFold

C	15.43201400	0.80105300	-29.77898100
C	16.10356500	-0.57530900	-29.67440400
O	17.27379200	-0.75657800	-29.35551700
H	14.60531600	0.62288500	-29.12343300
N	15.21407300	-1.59258800	-29.83136400
C	15.43029900	-3.03830200	-29.79300600
C	15.89329300	-3.66069600	-28.47110900
O	15.62034000	-4.84752400	-28.25525300
C	16.20640900	-3.56540900	-31.00476800
C	15.23779600	-3.77556100	-32.17702400
N	15.72609300	-4.47883000	-33.21500300
O	14.09163400	-3.34310900	-32.13099600
H	14.30222000	-1.34464800	-30.17972700
H	14.42441600	-3.44185900	-29.89474900
H	16.68782800	-4.51552600	-30.77044900
H	16.99829700	-2.86912500	-31.28370600
H	15.07041900	-4.96215400	-33.83174000
H	16.59823400	-4.96615600	-33.08554500
N	16.51980200	-2.89483600	-27.58117800
C	16.89789000	-3.35694600	-26.25401900
H	16.89103300	-2.01198800	-27.91643000
H	16.15062500	-3.99128900	-25.82504000
C	18.83908200	-12.26303400	-25.94106200
C	18.80163000	-11.35301300	-27.15256500
O	19.59771000	-11.51357600	-28.07275600
N	17.95955100	-10.28715100	-27.15004600
C	17.92274800	-9.38068900	-28.29536600
C	19.19198000	-8.52455500	-28.40896300
O	19.47348600	-7.94997700	-29.45042900
C	16.65527100	-8.50491300	-28.26999400
C	16.13721100	-8.20084100	-29.63718600
C	16.19747300	-8.88673500	-30.82194400
N	15.37056600	-7.08372600	-29.89949500
C	15.01042200	-7.12424400	-31.20300100
N	15.49788900	-8.20032000	-31.78865300
H	17.25140800	-10.21175000	-26.43811000
H	17.93066200	-9.98927400	-29.19838600
H	16.84055200	-7.57483400	-27.72724700
H	15.88406800	-9.04121500	-27.70664400
H	16.71967400	-9.79315800	-31.06923100
H	15.25290400	-6.28352100	-29.27064500
H	14.42458000	-6.36808700	-31.69524000
N	19.98155100	-8.45776500	-27.31386500
C	21.29076700	-7.85290500	-27.38722900
C	22.31813500	-8.63117300	-28.21221700
O	23.33329900	-8.05863200	-28.59547100
H	19.70906000	-8.96623700	-26.49086800
H	21.68649600	-7.73862500	-26.37853400
H	21.22622900	-6.86130300	-27.83238400
N	22.06866100	-9.92637600	-28.46567000
C	22.98452100	-10.70194100	-29.28856200
C	22.98596400	-10.25977300	-30.75855400
O	24.00555000	-10.35916400	-31.42310700
C	22.67099500	-12.19095800	-29.18271500
H	21.18859600	-10.35316300	-28.19891000
H	23.99901600	-10.50641000	-28.94486500
H	23.36206900	-12.74852200	-29.81305200
H	22.78344200	-12.53149400	-28.15294600
H	21.65046300	-12.40238600	-29.50106800
N	21.81019500	-9.80792600	-31.24732400
C	21.67167500	-9.35299600	-32.61041300
C	21.78212600	-7.83382800	-32.76707100
O	22.00381600	-7.36116600	-33.87293700
C	20.36992000	-9.89582400	-33.22597100
C	20.47288400	-11.38300200	-33.54268600
O	19.33426400	-9.63949900	-32.29707200
H	20.96844500	-9.83437200	-30.69118600
H	22.51622000	-9.74569300	-33.17376300
H	20.19574500	-9.33826700	-34.15274600
H	19.50347700	-11.75347900	-33.87947400
H	21.21642400	-11.56440700	-34.32292100
H	20.76112800	-11.94209000	-32.65002900
H	18.48032500	-9.87515700	-32.73418900
N	21.65789500	-7.07299000	-31.64257600
C	21.61815900	-5.63077600	-31.75793200
C	22.59921500	-4.84084400	-30.89334600
O	22.68660100	-3.62885700	-31.07352100
C	20.19115100	-5.08206100	-31.55988800
C	19.24580800	-5.70592400	-32.51556500
C	18.22419800	-6.58585700	-32.31647700
N	19.35110200	-5.58185500	-33.89100900
C	18.42159900	-6.37297600	-34.46901100
N	17.71343400	-6.97758100	-33.53604700
H	21.20081800	-7.50012300	-30.84722000
H	21.93289000	-5.41589400	-32.77881100
H	20.23096100	-3.99690200	-31.66296600
H	19.84949600	-5.30843200	-30.54854300
H	17.88085400	-7.00167600	-31.38771200
H	20.08410800	-5.09722400	-34.38083500
H	18.26394700	-6.50824000	-35.52584000
N	23.33503100	-5.49724800	-29.98596400
C	24.38306400	-4.78808100	-29.26494200
H	23.25105400	-6.49591100	-29.85035900
H	23.96570800	-4.02506300	-28.64154600
H	24.92566000	-5.50736300	-28.65650200
H	25.03326000	-4.34160100	-29.98804300
H	19.76434300	-11.98830200	-25.47928400
H	18.01005600	-12.14083100	-25.24788400
H	18.90646500	-13.26660500	-26.30586200
H	16.08708800	1.59359000	-29.43911500
H	15.07064800	0.96077300	-30.77338300
H	17.84491900	-3.90211000	-26.27114300
H	17.00496500	-2.47652200	-25.65548900
O	14.49004300	-6.84382600	-34.30330100
O	12.73025300	-6.64416100	-35.67731300
O	16.66292800	-8.05130500	-36.01629800
O	16.49387800	-8.20840600	-38.23511000
O	17.00604400	-10.06829000	-33.58403000
O	16.89995900	-12.23878300	-34.12951800
N	14.55236600	-9.53654800	-34.62662400
C	13.83882300	-8.75235600	-35.65134900
C	14.54782600	-8.74043000	-37.00898200
C	15.16843200	-10.82316300	-34.94328000
C	13.65115000	-7.28022900	-35.16780000
C	16.02633900	-8.29307600	-37.09538800
C	16.46841500	-11.08829700	-34.14312100
H	12.82925000	-9.14341100	-35.81384000
H	14.48612900	-9.72260800	-37.48323900
H	13.97830600	-8.07053000	-37.65289900
H	13.97446700	-9.61695400	-33.79966100
H	14.49513500	-11.67034800	-34.79045200
H	15.46252900	-10.83377200	-35.99049600
Zn	16.09506200	-8.20072300	-33.98954300

Co^{2+}[CM-Asp]$^{3-}$[HGATH] AlphaFold

C	15.43197800	0.80101200	-29.77898600
C	16.31143600	-0.43928900	-29.76306700
O	17.53077500	-0.40596000	-29.64822200
H	14.60534600	0.62290800	-29.12338900
N	15.62713200	-1.61289700	-29.86700100
C	16.24620800	-2.92631700	-30.00037400
C	16.97162800	-3.41811500	-28.73319700
O	17.86329400	-4.26113300	-28.80328900
C	17.10760000	-3.06497400	-31.24724900
C	16.24621000	-3.12947200	-32.49288400
N	16.74049400	-2.51129400	-33.58072000
O	15.17302900	-3.72891500	-32.50651100
H	14.63407400	-1.56183700	-30.02446500
H	15.41321200	-3.62182600	-30.12063200
H	17.64608300	-4.01023500	-31.18180300
H	17.84355800	-2.26618000	-31.30252600
H	16.25595000	-2.59036100	-34.46022500
H	17.62845400	-2.04088600	-33.56297200
N	16.48344300	-2.93378200	-27.58440700
C	16.89792900	-3.35705400	-26.25409100
H	15.74942800	-2.24740600	-27.65166900
H	16.15058400	-3.99122100	-25.82501000
C	18.83906000	-12.26298600	-25.94104400
C	18.73243800	-11.30943300	-27.10826900
O	19.68302400	-11.15131100	-27.88090600
N	17.60970000	-10.57421200	-27.25146100
C	17.48964700	-9.64064500	-28.36144100
C	18.46619700	-8.46227900	-28.23964100
O	18.90696700	-7.89303800	-29.23043800
C	16.03234600	-9.16231000	-28.51083800
C	15.80567200	-8.54331800	-29.84744800
C	16.03263300	-9.04508400	-31.09714800
N	15.37983200	-7.24875100	-30.05278300
C	15.36438100	-6.99945900	-31.38108000
N	15.76841000	-8.07452100	-32.03692200
H	16.82464600	-10.72883100	-26.63889500
H	17.79256700	-10.14967500	-29.27504200
H	15.77553900	-8.46400500	-27.71299600
H	15.38468000	-10.03386300	-28.39355300
H	16.41994800	-10.00565600	-31.37734400
H	15.13140300	-6.58806300	-29.33387300
H	15.10323100	-6.04923100	-31.81551900
N	18.82497100	-8.09580300	-26.99153000
C	19.84912400	-7.09360100	-26.80660800
C	21.23167500	-7.53931200	-27.28108700
O	22.06782100	-6.70362700	-27.62230600

H	18.45918200	-8.59540100	-26.19803300
H	19.91422900	-6.84912600	-25.74707300
H	19.60070600	-6.18578900	-27.35373000
N	21.47801600	-8.85880300	-27.26681300
C	22.74702500	-9.40913100	-27.70259100
C	22.89431500	-9.45024900	-29.22632200
O	24.00101200	-9.29492400	-29.73543200
C	22.95998000	-10.79958000	-27.10782300
H	20.72224300	-9.50787800	-27.09898300
H	23.53431900	-8.73761600	-27.36544900
H	23.91117200	-11.20458300	-27.45035000
H	22.97941900	-10.73809000	-26.01963100
H	22.15768100	-11.47341900	-27.40669800
N	21.78323500	-9.68073700	-29.95453900
C	21.85614000	-9.69557500	-31.40098300
C	21.95986900	-8.29151400	-32.00903700
O	22.45184100	-8.15549700	-33.12467500
C	20.71439500	-10.51169900	-32.02520900
C	20.83863300	-11.99408100	-31.68950500
O	19.48185000	-9.98833900	-31.57127700
H	20.88721200	-9.81569300	-29.50643500
H	22.79624900	-10.16879900	-31.68219600
H	20.80662400	-10.38084200	-33.10824600
H	20.02624200	-12.54053200	-32.16855500
H	21.78857300	-12.40107400	-32.04222600
H	20.76947000	-12.14998700	-30.61188400
H	18.81478600	-10.03070600	-32.29945500
N	21.53183200	-7.25526400	-31.25415400
C	21.64615300	-5.88629500	-31.71124700
C	22.80175400	-5.08193100	-31.10033700
O	23.17549800	-4.06140400	-31.68165500
C	20.32180600	-5.12086200	-31.51979100
C	19.27228300	-5.60726200	-32.45007300
C	18.37498100	-6.62936800	-32.34231400
N	19.09975300	-5.10581800	-33.72591400
C	18.12694400	-5.81204600	-34.33960200
N	17.66962200	-6.74436500	-33.52532100
H	20.96734400	-7.46147000	-30.44036700
H	21.87490200	-5.92870400	-32.77445200
H	20.51336500	-4.06068100	-31.68569700
H	19.97605500	-5.23000600	-30.49074100
H	18.22490300	-7.28966700	-31.50788600
H	19.59851600	-4.32947700	-34.12951100
H	17.78917400	-5.63646800	-35.34394100
N	23.34023600	-5.52538200	-29.96015800
C	24.38308100	-4.78807000	-29.26492100
H	22.88964300	-6.27195400	-29.44878200
H	23.96571100	-4.02506900	-28.64153800
H	24.94873500	-5.48133600	-28.64559900
H	25.03325000	-4.34160900	-29.98805700
H	19.76437000	-11.98834300	-25.47931300
H	18.01653100	-12.17889500	-25.23427600
H	18.90647800	-13.26661400	-26.30583700
H	16.00545700	1.66181300	-29.44837700
H	15.07065500	0.96081100	-30.77337900
H	17.84371900	-3.88657100	-26.32369300
H	17.00493800	-2.47649800	-25.65551700
O	14.46526800	-6.87197600	-34.44623200
O	12.55735700	-7.23996700	-35.56867000
O	16.89256600	-8.04180500	-35.81238600
O	16.72846800	-8.12974400	-38.01984200
O	17.59591200	-9.96720900	-33.51553500
O	17.39841600	-12.18872000	-33.74676400
N	15.01407500	-9.53245700	-34.38654500
C	14.14868900	-9.01326400	-35.47437500
C	14.86980900	-8.96483800	-36.82143600
C	15.65666900	-10.83997700	-34.62473000
C	13.66703400	-7.58273900	-35.11898800
C	16.26128400	-8.32043900	-36.88631300
C	16.99612000	-11.02380100	-33.88630100
H	13.26457100	-9.64602800	-35.58238800
H	14.97458000	-9.96719500	-37.24215300
H	14.22886700	-8.42781500	-37.52081400
H	14.43495400	-9.61554800	-33.55748600
H	14.98810600	-11.66079900	-34.36357200
H	15.88762900	-10.93866500	-35.68302400
Co	16.31022600	-8.10443300	-33.94069700

Co^{2+}[CM-Asp]$^{3-}$[HGATH] AlphaFold SAS

C	15.43200800	0.80099300	-29.77897800
C	16.11796500	-0.56408500	-29.65734400
O	17.27788900	-0.72449200	-29.28967400
H	14.60533200	0.62290700	-29.12340700
N	15.25229900	-1.59468400	-29.84535500
C	15.50805300	-3.03406700	-29.82451000
C	15.93409900	-3.66434700	-28.49283400
O	15.66506200	-4.85539600	-28.29445500
C	16.35999600	-3.50531800	-31.00966000
C	15.46090700	-3.63329300	-32.24575400
N	15.93887400	-4.40675100	-33.23739800
O	14.36828200	-3.07766800	-32.28453500
H	14.37146900	-1.36856600	-30.27916600
H	14.52057400	-3.46608000	-29.97442000
H	16.82172600	-4.46916100	-30.79334600
H	17.16887900	-2.79759900	-31.19701300
H	15.27082800	-4.85046000	-33.87093800
H	16.74673200	-4.97605600	-33.03979600
N	16.53100600	-2.89677400	-27.58441300
C	16.89789800	-3.35701300	-26.25407000
H	16.89662900	-2.00697800	-27.90981100
H	16.15059500	-3.99124600	-25.82502700
C	18.83903400	-12.26298700	-25.94104100
C	18.80046300	-11.34951300	-27.14876700
O	19.61574900	-11.48795200	-28.05561700
N	17.93360700	-10.30378300	-27.15802300
C	17.90089800	-9.39620400	-28.30195800
C	19.15116400	-8.50709700	-28.38371200
O	19.44559300	-7.92486400	-29.41671700
C	16.60741000	-8.55925800	-28.31045900
C	16.16922500	-8.21025300	-29.69423700
C	16.31529700	-8.86455900	-30.88664900
N	15.44232500	-7.07293600	-29.98170800
C	15.18821900	-7.06149200	-31.30840200
N	15.71625000	-8.12748600	-31.88487200
H	17.21173200	-10.24595000	-26.45833100
H	17.94917700	-10.00395000	-29.20433300
H	16.73275100	-7.64846300	-27.71998600
H	15.82298800	-9.14381300	-27.81821400
H	16.83869000	-9.77113600	-31.12385000
H	15.29854100	-6.28349600	-29.34428800
H	14.65928800	-6.28728600	-31.83407500
N	19.91197000	-8.42440100	-27.26875300
C	21.20884300	-7.79074500	-27.30945500
C	22.28138700	-8.55635000	-28.08764200
O	23.29668600	-7.96589800	-28.44192100
H	19.63074500	-8.93963900	-26.45298000
H	21.56932700	-7.65153700	-26.29068900
H	21.13509100	-6.80840200	-27.77292700
N	22.07048100	-9.86069000	-28.32986300
C	23.03369700	-10.62866000	-29.10425500
C	23.05732100	-10.23655500	-30.58819100
O	24.09575600	-10.32450200	-31.22492000
C	22.77223000	-12.12415300	-28.95512300
H	21.18710700	-10.30038200	-28.09761000
H	24.03066100	-10.38368100	-28.74192600
H	23.49742700	-12.67633900	-29.55095000
H	22.87409800	-12.42576300	-27.91210400
H	21.76753700	-12.38348000	-29.28802600
N	21.88051600	-9.84103400	-31.12005400
C	21.75843600	-9.44345100	-32.50273300
C	21.86565500	-7.93177900	-32.72123800
O	22.11311500	-7.50439700	-33.83958900
C	20.45982100	-10.00966900	-33.10274200
C	20.56320600	-11.50776300	-33.36484600
O	19.42343400	-9.72079500	-32.18687000
H	21.02557500	-9.87352200	-30.58439600
H	22.60851200	-9.85690300	-33.04238600
H	20.28792200	-9.48707900	-34.05015000
H	19.59389900	-11.88640600	-33.69188200
H	21.30801200	-11.71973000	-34.13600700
H	20.84564900	-12.03550400	-32.45165500
H	18.56950600	-9.97961500	-32.61674800
N	21.70725100	-7.12635000	-31.63294300
C	21.67834000	-5.68987100	-31.80545300
C	22.65204100	-4.87698800	-30.95279000
O	22.76963900	-3.67684000	-31.18696000
C	20.25422500	-5.12280600	-31.64432900
C	19.30179800	-5.77412000	-32.57447600
C	18.28528100	-6.64848900	-32.33072100
N	19.37079100	-5.67736500	-33.95190600
C	18.42052200	-6.47573800	-34.49026900
N	17.73516100	-7.06353800	-33.52829500
H	21.23069600	-7.52314800	-30.83348600
H	22.00689200	-5.51600600	-32.82965300
H	20.30493200	-4.04327800	-31.79306700
H	19.90501500	-5.30094100	-30.62570200
H	17.97987700	-7.04357500	-31.38124600
H	20.08019400	-5.19016500	-34.47215900
H	18.23723800	-6.62298900	-35.53979200
N	23.34319400	-5.50366300	-29.99062000
C	24.38303500	-4.78800200	-29.26496700
H	23.23442800	-6.49297000	-29.81450800
H	23.96571800	-4.02509200	-28.64150500
H	24.92583000	-5.50448500	-28.65342400
H	25.03328700	-4.34164800	-29.98804800
H	19.76437400	-11.98834500	-25.47932000
H	18.01014200	-12.14334500	-25.24716400
H	18.90649700	-13.26661500	-26.30583200
H	16.07829400	1.60121100	-29.43940300
H	15.07064200	0.96081800	-30.77337300
H	17.84477100	-3.90303100	-26.26496000
H	17.00496000	-2.47650400	-25.65551300
O	14.54758300	-6.67556600	-34.22499300
O	12.55574100	-6.69574000	-35.27052000
O	16.67149500	-8.23778200	-35.72512800
O	16.36850100	-8.10776300	-37.91917900
O	17.11448200	-10.20225500	-33.41349600
O	16.67459300	-12.37436200	-33.78246200
N	14.63863800	-9.40237500	-34.24403300
C	13.82117900	-8.71104700	-35.27311800
C	14.47457200	-8.74546700	-36.65411200
C	15.08239100	-10.78095200	-34.53427200
C	13.59898900	-7.22127000	-34.87590700
C	15.94750500	-8.31499800	-36.77940700
C	16.40692100	-11.17367800	-33.83480400
H	12.83402800	-9.17662000	-35.34482300
H	14.40837300	-9.74427500	-37.09314000
H	13.88972700	-8.10032900	-37.30841000
H	14.10640700	-9.41105400	-33.38078600
H	14.31366600	-11.51268700	-34.27865600
H	15.27563700	-10.87143700	-35.60014500
Co	16.16850100	-8.23097300	-33.84233500

Ni^{2+}[CM-Asp]$^{3-}$[HGATH] AlphaFold

C	15.43198700	0.80103800	-29.77900100
C	16.27780800	-0.46102000	-29.78000700
O	17.50138300	-0.46820400	-29.72754700
H	14.60532800	0.62289400	-29.12341500
N	15.54651000	-1.60990400	-29.83173700
C	16.10635100	-2.94625200	-29.97639400
C	16.87422400	-3.44858400	-28.73675000
O	17.72156300	-4.33297200	-28.83397100
C	16.89841100	-3.13413200	-31.26359800
C	15.97434100	-3.17869400	-32.46400600
N	16.36464100	-2.47056900	-33.53780600
O	14.93726900	-3.84107600	-32.45507200
H	14.54880200	-1.52267000	-29.93400000
H	15.24168400	-3.60939300	-30.04144700
H	17.40068300	-4.09904700	-31.20738200
H	17.66194600	-2.36629600	-31.36235000
H	15.82493000	-2.51765700	-34.38712800
H	17.21876900	-1.94099600	-33.54048800
N	16.46654600	-2.92075100	-27.57628100
C	16.89796100	-3.35699400	-26.25401700
H	15.75606900	-2.20844800	-27.62329100
H	16.15060000	-3.99125700	-25.82503600
C	18.83905300	-12.26302200	-25.94104900
C	18.72102400	-11.31869100	-27.11105800
O	19.64074400	-11.19999800	-27.92680700
N	17.62101200	-10.54391300	-27.20863000
C	17.49790000	-9.59526500	-28.30297100
C	18.53950700	-8.46956100	-28.20892200
O	18.97350100	-7.91190900	-29.20949200
C	16.06231800	-9.04684300	-28.37345300
C	15.80251900	-8.38748500	-29.68338200
C	16.04673000	-8.82162100	-30.95375000
N	15.28585900	-7.11825500	-29.82610100
C	15.23239200	-6.80818600	-31.13611100
N	15.69645600	-7.82802800	-31.84238700
H	16.86048400	-10.66313600	-26.55854300
H	17.73332300	-10.10854600	-29.23388500
H	15.87985000	-8.35142900	-27.55306500
H	15.37733500	-9.88719000	-28.23862600
H	16.48632000	-9.74360100	-31.27996800
H	14.99643000	-6.51319200	-29.07402800
H	14.90435200	-5.85868500	-31.52770200
N	18.96336700	-8.14211200	-26.97098900
C	20.04163300	-7.19610500	-26.80615500
C	21.38301400	-7.68864800	-27.34591200
O	22.24893300	-6.87599500	-27.66950600
H	18.60396400	-8.63765100	-26.17219500
H	20.16002600	-6.97940800	-25.74529900
H	19.81720800	-6.26434300	-27.32230300
N	21.56317600	-9.01637700	-27.41065900
C	22.78794600	-9.59955500	-27.92367800
C	22.87003700	-9.58153400	-29.45273600
O	23.96388100	-9.48081900	-30.00096600
C	22.96776300	-11.02196000	-27.39724800
H	20.78525200	-9.64032700	-27.24400700
H	23.61630800	-8.97565500	-27.59378400
H	23.88702800	-11.44789900	-27.79664000
H	23.03414600	-11.00829200	-26.30927200
H	22.12720600	-11.65074200	-27.68863300
N	21.71732700	-9.70335500	-30.14309800
C	21.73229900	-9.65075900	-31.59044100
C	21.86387700	-8.22236900	-32.13467500
O	22.30753200	-8.04650500	-33.26450300
C	20.54043000	-10.38981000	-32.21259900
C	20.58555500	-11.88468600	-31.91364100
O	19.34916100	-9.80140000	-31.73086200
H	20.83082300	-9.78039200	-29.66448100
H	22.64192700	-10.14529400	-31.92840500
H	20.62848900	-10.23952500	-33.29447200
H	19.73407300	-12.37454200	-32.38715100
H	21.50220700	-12.33725300	-32.29747100
H	20.52876100	-12.06397500	-30.83900400
H	18.61705200	-9.97930900	-32.37371700
N	21.50905800	-7.21276000	-31.30858100
C	21.62689700	-5.82666500	-31.70872100
C	22.80925800	-5.05866600	-31.10531200
O	23.19837600	-4.03988200	-31.67977100
C	20.32457400	-5.05504900	-31.41868200
C	19.22476100	-5.47571000	-32.31984600
C	18.30352100	-6.47395800	-32.21201600
N	18.99188000	-4.89351000	-33.55152600
C	17.96307900	-5.52539100	-34.14530000
N	17.52407700	-6.49035000	-33.35519900
H	20.97569400	-7.44417100	-30.48091200
H	21.81036400	-5.82214900	-32.78113000
H	20.52119700	-3.98968700	-31.53906300
H	20.02456300	-5.21442600	-30.38204800
H	18.18159700	-7.17695800	-31.40846500
H	19.49122600	-4.10866400	-33.93898100
H	17.56706400	-5.28319700	-35.11319200
N	23.33960700	-5.51708900	-29.96775600
C	24.38305700	-4.78805600	-29.26495500
H	22.89239600	-6.27208900	-29.46476000
H	23.96570700	-4.02507300	-28.64153000
H	24.94479700	-5.48531700	-28.64694200
H	25.03327000	-4.34161300	-29.98804100
H	19.76435300	-11.98831500	-25.47929600
H	18.01936400	-12.17932300	-25.23096000
H	18.90648200	-13.26660400	-26.30586300
H	16.02099800	1.65058800	-29.44786100
H	15.07065600	0.96078500	-30.77338400
H	17.84348800	-3.88522000	-26.33381200
H	17.00492600	-2.47651500	-25.65549200
O	12.67064800	-7.45204200	-33.39461200
O	12.71745000	-6.45265500	-35.40983100
O	16.35295000	-7.35371200	-35.60142200
O	15.84830100	-7.01601600	-37.72080400
O	17.35854600	-10.06366400	-33.52348000
O	16.32930300	-11.95250700	-32.88473900
N	14.67586400	-9.08435400	-34.12226700
C	13.72341200	-8.62446200	-35.16770100
C	14.37236000	-8.43459000	-36.52427500
C	15.15797500	-10.47021600	-34.34131200
C	12.97415700	-7.37636500	-34.61109700
C	15.58681700	-7.52311500	-36.62629300
C	16.38368300	-10.87010800	-33.49459900
H	12.95460000	-9.40052400	-35.27567200
H	14.68986800	-9.40020800	-36.92918000
H	13.61874200	-8.05371500	-37.20826400
H	14.12466900	-9.04834700	-33.26513400
H	14.33813500	-11.16519300	-34.15790500
H	15.45932800	-10.56482700	-35.38369400
Ni	16.07629900	-7.73590800	-33.74930300

Ni^{2+}[CM-Asp]$^{3-}$[HGATH] AlphaFold SAS

C	15.43198000	0.80102900	-29.77900300
C	16.18625600	-0.52524600	-29.69330400
O	17.36380100	-0.62515400	-29.36378800
H	14.60532800	0.62289800	-29.12341400
N	15.40219900	-1.61375900	-29.93015100
C	15.78473400	-3.02834500	-29.90396700
C	15.93457900	-3.65766500	-28.50776100
O	15.53539300	-4.80349800	-28.29924800
C	17.04939300	-3.33473000	-30.71536700
C	16.85730500	-2.88214400	-32.14987600
N	17.72238700	-1.92584100	-32.57859600
O	15.98639100	-3.35683300	-32.85659500
H	14.43810100	-1.43815500	-30.15864300
H	14.96022600	-3.55973400	-30.37397100
H	17.18822400	-4.41497700	-30.72944200
H	17.92008400	-2.87716500	-30.25454400
H	17.55180200	-1.50234000	-33.47698700
H	18.29175000	-1.42316500	-31.91775000
N	16.55414600	-2.90712400	-27.59287500
C	16.89800600	-3.35701800	-26.25399700
H	16.96497000	-2.03851900	-27.91484200
H	16.15058300	-3.99124300	-25.82504500
C	18.83906400	-12.26301600	-25.94106300
C	18.75558000	-11.23662100	-27.05803100
O	19.74630300	-10.98186700	-27.74120800
N	17.61751000	-10.51542200	-27.20065200
C	17.50025600	-9.49739300	-28.24573700
C	18.38994200	-8.27569600	-27.98724200
O	18.75764000	-7.55246900	-28.89881600
C	16.02651900	-9.05512800	-28.39586300
C	15.77386900	-8.44343500	-29.73012400
C	15.91175100	-9.01659600	-30.96130500
N	15.42858900	-7.12831000	-29.96289400
C	15.37711400	-6.94096400	-31.29607400
N	15.67901000	-8.06570400	-31.92646400
H	16.79315900	-10.79270900	-26.69244300
H	17.85937600	-9.91192800	-29.18858500

H 15.75084900 -8.36816900 -27.59315900
H 15.39905800 -9.94391000 -28.28844800
H 16.18748100 -10.02889600 -31.19653000
H 15.34172100 -6.37410500 -29.27974200
H 15.15988200 -5.99914300 -31.76891700
N 18.72214700 -8.03305300 -26.69540300
C 19.67134400 -6.99378100 -26.36708900
C 21.11689600 -7.29559800 -26.78081000
O 21.92362300 -6.38693800 -26.87975300
H 18.41335200 -8.67561700 -25.98642500
H 19.65209300 -6.82123000 -25.29142100
H 19.39772200 -6.06657700 -26.86660500
N 21.41047300 -8.60180000 -26.97734100
C 22.69112600 -9.03009300 -27.50623500
C 22.73396400 -9.00274800 -29.03880300
O 23.73344100 -8.57823900 -29.61466400
C 23.05985500 -10.41671600 -26.98331700
H 20.66233000 -9.27928100 -26.99019500
H 23.43076800 -8.29732300 -27.19288400
H 24.01557000 -10.72925200 -27.40373400
H 23.14855600 -10.39378400 -25.89664000
H 22.30064400 -11.14938600 -27.25824600
N 21.65632600 -9.46313700 -29.70174400
C 21.64951300 -9.50158100 -31.15420600
C 21.71228500 -8.10771800 -31.79100900
O 22.15283000 -7.97532000 -32.92181100
C 20.46614500 -10.31304100 -31.69571800
C 20.54319600 -11.78643800 -31.30345500
O 19.25997700 -9.73198300 -31.24768400
H 20.84828700 -9.81348300 -29.20478800
H 22.57072500 -9.97933200 -31.49121800
H 20.53212100 -10.23363600 -32.78592800
H 19.68282800 -12.30450800 -31.72762900
H 21.45866100 -12.25098900 -31.67917500
H 20.50684600 -11.90555500 -30.21954400
H 18.56573500 -9.98338000 -31.92269700
N 21.30206400 -7.07285200 -31.01843600
C 21.39414400 -5.70228000 -31.45738700
C 22.43943300 -4.85889200 -30.70836600
O 22.44844700 -3.63829700 -30.85285500
C 20.03098600 -4.99948200 -31.39209400
C 19.04971100 -5.55582200 -32.35506200
C 18.24530500 -6.65228000 -32.28074100
N 18.81112900 -5.01500800 -33.60804600
C 17.87886600 -5.76318100 -34.23512000
N 17.51851500 -6.75899100 -33.45124800
H 20.77668700 -7.28366900 -30.18112100
H 21.73012800 -5.73967700 -32.49539200
H 20.19593200 -3.93709600 -31.56723400
H 19.63783000 -5.10416200 -30.37845500
H 18.18106800 -7.37339100 -31.48627200
H 19.20746700 -4.16030300 -33.95976300
H 17.49142500 -5.58822100 -35.22212600
N 23.32443600 -5.51356100 -29.94703100
C 24.38304200 -4.78805100 -29.26496000
H 23.31058900 -6.52337200 -29.89877300
H 23.96571200 -4.02507200 -28.64153100
H 24.93961000 -5.49326800 -28.65225900
H 25.03327500 -4.34161900 -29.98804000
H 19.76435000 -11.98831800 -25.47928900
H 18.01960600 -12.19208800 -25.22751900
H 18.90647600 -13.26660600 -26.30585800
H 16.05474100 1.61982600 -29.43835500
H 15.07065900 0.96079200 -30.77338400
H 17.84552500 -3.90295700 -26.25476100
H 17.00490500 -2.47650800 -25.65549900
O 12.76672500 -8.22951000 -33.54933700
O 12.37382400 -7.54477000 -35.66672900
O 16.46776600 -7.76112800 -35.67404100
O 16.03514000 -7.56422300 -37.82717900
O 17.60457300 -10.29895300 -33.22132300
O 16.41502700 -12.01030900 -32.37743200
N 14.91083300 -9.58587400 -34.20116500
C 13.95236100 -9.31116100 -35.29758800
C 14.61024400 -9.03339500 -36.63643300
C 15.59732200 -10.89345000 -34.32923500
C 12.93535700 -8.22741700 -34.79376800
C 15.76309800 -8.03561000 -36.72668200
C 16.63386800 -11.11281100 -33.20592200
H 13.34012000 -10.21648400 -35.42064300
H 15.00046700 -9.96164900 -37.06766300
H 13.83198300 -8.67843500 -37.30750600
H 14.30948300 -9.55912800 -33.37251800
H 14.85189000 -11.69161500 -34.31049100
H 16.11525500 -10.90619300 -35.28765300
Ni 16.14754100 -8.10045800 -33.84469500

Cu^{2+}[CM-Asp]$^{3-}$[HGATH] AlphaFold SAS

C 15.43200000 0.80110700 -29.77899400
C 16.11063400 -0.57416100 -29.84335200
O 17.28751100 -0.78399200 -30.05742900
H 14.60532700 0.62286900 -29.12342300
N 15.19776200 -1.58120200 -29.63130700
C 15.40269400 -3.01591400 -29.74195400
C 16.22994900 -3.63802700 -28.62069800
O 16.57589700 -4.81976300 -28.68843800
C 15.78393600 -3.55006100 -31.12314100
C 14.84727600 -4.70543600 -31.51137800
N 15.20235100 -5.41360800 -32.59098700
O 13.82029800 -4.92759500 -30.87372800
H 14.23760800 -1.30035200 -29.52978500
H 14.41148400 -3.44902800 -29.56221300
H 16.82019000 -3.88325600 -31.15951300
H 15.66932100 -2.75828000 -31.86454600
H 14.71796200 -6.29722100 -32.78957300
H 16.13753400 -5.31957600 -32.94893400
N 16.43534200 -2.87253100 -27.54761400
C 16.89784300 -3.35704000 -26.25402300
H 16.10121300 -1.92620700 -27.60506100
H 16.15060900 -3.99123800 -25.82499300
C 18.83924100 -12.26300400 -25.94117600
C 18.88199200 -11.23227000 -27.06497300
O 19.81658600 -11.22621700 -27.86149300
N 18.01352400 -10.19461500 -26.99019100
C 18.12202400 -9.02765600 -27.86253500
C 19.47438200 -8.32757400 -27.72867000
O 19.94196900 -7.67495800 -28.65098200
C 17.02200800 -7.99966700 -27.53133400
C 15.70800700 -8.25334500 -28.19362300
C 14.89522200 -9.34939300 -28.32256300
N 15.08466900 -7.26280100 -28.92206000
C 13.95283200 -7.77851000 -29.45503100
N 13.80595400 -9.04572400 -29.11392700
H 17.20252000 -10.28027500 -26.40097000
H 18.04178100 -9.33425400 -28.90336200
H 17.37105500 -7.01828900 -27.85109000
H 16.91689100 -7.93851000 -26.44221900
H 15.05211000 -10.34613800 -27.94477400
H 15.42722300 -6.30947500 -29.01354000
H 13.31435600 -7.20017500 -30.10084700
N 20.10653700 -8.39702500 -26.53438200
C 21.40129500 -7.77071700 -26.37954200
C 22.51490700 -8.42158000 -27.20550800
O 23.46155200 -7.75458100 -27.60213500
H 19.70323800 -8.94330800 -25.79295300
H 21.68793000 -7.80931800 -25.32854300
H 21.35798600 -6.72808800 -26.68806500
N 22.40260200 -9.75137300 -27.40049200
C 23.38606200 -10.48954300 -28.17350400
C 23.25878000 -10.27445700 -29.68668200
O 24.24493300 -10.34207200 -30.40580100
C 23.29499800 -11.97868400 -27.84837200
H 21.51562900 -10.20572900 -27.22239500
H 24.37055700 -10.10365300 -27.91623800
H 24.01463900 -12.53054000 -28.45141100
H 23.51768100 -12.14945100 -26.79439400
H 22.29386500 -12.35773200 -28.05896000
N 22.01025100 -10.05628900 -30.14585200
C 21.72794100 -9.88280400 -31.54969300
C 21.80670900 -8.42775600 -32.01774500
O 21.94970500 -8.18461300 -33.21487900
C 20.31839300 -10.42368600 -31.84675400
C 20.20090700 -11.92427400 -31.62269300
O 19.47228800 -9.68959000 -30.99390500
H 21.21262600 -10.07808500 -29.52477200
H 22.47164300 -10.42352800 -32.13199600
H 20.10251100 -10.19914200 -32.89891500
H 19.18109100 -12.24308700 -31.84082300
H 20.88319400 -12.47020300 -32.27809300
H 20.41464600 -12.17993600 -30.58520500
H 18.56477300 -10.06510400 -30.95618100
N 21.66372400 -7.46844900 -31.07349800
C 21.51999600 -6.07654200 -31.45518400
C 22.51335400 -5.10690400 -30.80392100
O 22.51028800 -3.92967000 -31.14728900
C 20.07978900 -5.56652300 -31.24634800
C 19.06066300 -6.46746000 -31.85291000
C 17.85380000 -6.89429100 -31.38060400
N 19.23101800 -7.12818800 -33.05788100
C 18.17193300 -7.93951100 -33.25721900
N 17.31364300 -7.80210200 -32.26568300
H 21.25229100 -7.74936200 -30.18910200
H 21.76122300 -6.03098000 -32.51729400
H 20.03239900 -4.55523300 -31.65352100
H 19.86447900 -5.49944100 -30.17950900
H 17.36364100 -6.61838400 -30.46596800
H 20.10878200 -7.20433800 -33.55814400
H 18.04615800 -8.63323900 -34.07223300
N 23.36503700 -5.60751300 -29.89616900
C 24.38311300 -4.78814600 -29.26487700
H 23.28646300 -6.56936400 -29.60275900
H 23.96569700 -4.02503500 -28.64158700
H 24.97338400 -5.44661400 -28.62951500
H 25.03322200 -4.34157300 -29.98806000
H 19.76430500 -11.98830700 -25.47920500
H 18.00652500 -12.14894800 -25.25127100
H 18.90636700 -13.26662000 -26.30583900
H 16.09992300 1.58693900 -29.44762200
H 15.07064900 0.96076100 -30.77338500
H 17.84342000 -3.88761300 -26.34143000
H 17.00500600 -2.47650500 -25.65552200
O 14.32772200 -8.07990700 -32.82195900
O 12.47604100 -8.53411400 -33.99628200
O 16.29252400 -10.17073500 -33.94395700
O 15.81428900 -11.59100100 -35.60654900
O 16.88991500 -10.29741600 -30.77151800
O 16.71642000 -12.22255600 -29.64571200
N 14.39880400 -10.42949300 -31.67599700
C 13.55153600 -10.35979000 -32.88397300
C 14.07812400 -11.17830200 -34.06584000
C 15.00362200 -11.68671000 -31.23408300
C 13.41093300 -8.86633600 -33.28147300
C 15.52580600 -10.95520400 -34.58579200
C 16.30351700 -11.40771300 -30.45778000
H 12.54242700 -10.71624300 -32.65579200
H 13.97755200 -12.24589600 -33.85607200
H 13.40908100 -10.98145500 -34.90352200
H 13.90719100 -10.01143100 -30.88160500
H 14.32837500 -12.28004300 -30.61529400
H 15.28563500 -12.28174200 -32.10121500
Cu 15.86117000 -9.13024100 -32.04932900

Ni^{2+}[NTA]$^{3-}$[HGATH] AlphaFold

C 15.43201000 0.80102600 -29.77897300
C 16.16039300 -0.53887200 -29.82601400
O 17.36986200 -0.67930500 -29.95543600
H 14.60533100 0.62289400 -29.12341200
N 15.29854300 -1.59097200 -29.71068800
C 15.58723800 -3.01058200 -29.83638700
C 16.19228200 -3.66793700 -28.59357000
O 16.33923000 -4.89694900 -28.56266300
C 16.31636500 -3.39174400 -31.12476000
C 15.33546300 -3.82011300 -32.21235600
N 15.86966500 -4.08049000 -33.41730200
O 14.13781600 -3.98145500 -31.97989800
H 14.32359600 -1.36359000 -29.60975200
H 14.60640500 -3.48461600 -29.91217600
H 16.97114000 -4.24593900 -30.94119800
H 16.95238200 -2.58011000 -31.47266400
H 15.34003500 -4.68491400 -34.04857000
H 16.86922400 -4.04873300 -33.53160100
N 16.47213200 -2.87971500 -27.56248400
C 16.89792300 -3.35704300 -26.25406900
H 16.29307500 -1.89622200 -27.67076600
H 16.15059500 -3.99124200 -25.82502100
C 18.83903700 -12.26297600 -25.94101300
C 18.81364900 -11.39377100 -27.17574300
O 19.64089300 -11.57471900 -28.07346700
N 17.93935800 -10.36788600 -27.25640700
C 17.93854100 -9.52368400 -28.44108700
C 19.21271900 -8.67487600 -28.54935400
O 19.58868700 -8.23222300 -29.63238300
C 16.66782900 -8.66361000 -28.50396000
C 16.31542300 -8.26522700 -29.89780200
C 16.46669900 -8.89688700 -31.10197600
N 15.65774800 -7.08695200 -30.17793800
C 15.43896300 -7.02227900 -31.50279700
N 15.92635700 -8.10191300 -32.09272500
H 17.23973400 -10.23840900 -26.54297100
H 17.97522400 -10.17851000 -29.31035200
H 16.77361700 -7.77445000 -27.87958400
H 15.84600200 -9.24777100 -28.08099200
H 16.95292600 -9.82549600 -31.33173700
H 15.58532400 -6.29347100 -29.53547800
H 14.94862400 -6.20989500 -32.00380600
N 19.90788900 -8.47491000 -27.41716700
C 21.20765800 -7.84783700 -27.45287700
C 22.25655800 -8.62256500 -28.24591500
O 23.25269100 -8.03368100 -28.67114300
H 19.56792800 -8.86758600 -26.55509600
H 21.56902500 -7.73528100 -26.43173200
H 21.14958600 -6.85538700 -27.89812500
N 22.05807400 -9.93178300 -28.44759400
C 22.99761900 -10.69922100 -29.24629600
C 23.01823800 -10.26737700 -30.71651100
O 24.04665200 -10.39975100 -31.37219600
C 22.70312200 -12.19289700 -29.14226500
H 21.20488300 -10.38873900 -28.14056300
H 24.00491900 -10.49762600 -28.88409200
H 23.42263800 -12.74602100 -29.74358400
H 22.78922100 -12.52045800 -28.10638500
H 21.69727500 -12.41992500 -29.49287700
N 21.86393400 -9.79429500 -31.23029000
C 21.76418000 -9.37462500 -32.60843200
C 21.77447600 -7.85460300 -32.80717000
O 21.92185700 -7.41146900 -33.94100800
C 20.58367700 -10.04760000 -33.32551500
C 20.81463200 -11.54437500 -33.49019700
O 19.41506600 -9.78098500 -32.57132300
H 21.03305400 -9.73715800 -30.65831400
H 22.67812400 -9.70662300 -33.09923500
H 20.51423200 -9.58081200 -34.31423300
H 19.94807900 -11.99859800 -33.97163700
H 21.69367800 -11.73919200 -34.10768700
H 20.95592700 -12.01987200 -32.51823500
H 18.63625800 -10.14379800 -33.05034300
N 21.69206000 -7.06884700 -31.70473200
C 21.67774800 -5.62611900 -31.83454500
C 22.69485300 -4.85892800 -30.99184500
O 22.87840300 -3.66751300 -31.25690300
C 20.26918000 -5.03765600 -31.59255100
C 19.28697400 -5.60203000 -32.54553500
C 18.44915000 -6.66611700 -32.40805800
N 19.16737000 -5.21076000 -33.86704400
C 18.28553400 -6.02615900 -34.48089900
N 17.82565900 -6.91175000 -33.61368200
H 21.31412800 -7.48472800 -30.86326100
H 21.96009800 -5.42076000 -32.86527200
H 20.33233200 -3.95320800 -31.67678100
H 19.94805600 -5.27529000 -30.57742900
H 18.31261500 -7.28622300 -31.54285700
H 19.67463100 -4.46441400 -34.31560300
H 17.99061700 -5.94839100 -35.51042200
N 23.33942800 -5.48707100 -30.00579800
C 24.38302900 -4.78800100 -29.26496700
H 23.17308000 -6.46127300 -29.78222300
H 23.96572300 -4.02509100 -28.64150200
H 24.92228500 -5.51018600 -28.65822800
H 25.03328500 -4.34164800 -29.98805000
H 19.76438200 -11.98835800 -25.47932900
H 18.00102200 -12.12675100 -25.26314500
H 18.90648600 -13.26661000 -26.30584700
H 16.07680000 1.60615600 -29.44551700
H 15.07064200 0.96080900 -30.77337400
H 17.84478800 -3.89178600 -26.31608800
H 17.00493600 -2.47649400 -25.65552400
O 17.17876100 -10.64408300 -33.89100300
O 16.82192600 -8.05532100 -35.79146900
O 14.56281700 -6.35515600 -34.64087100
O 16.06249900 -12.33216500 -32.91594100
O 16.13723800 -8.70733900 -37.80913700
O 12.43126500 -6.67841000 -35.27164700
N 14.83040500 -9.24683800 -34.45853900
C 14.79035600 -10.49389900 -33.65327000
C 15.00553300 -9.57702500 -35.90088300
C 13.56134200 -8.49671400 -34.25139300
C 16.13748300 -11.22769200 -33.48469000
C 16.04821900 -8.70856800 -36.58295000
C 13.52232500 -7.04634500 -34.79342600
H 14.06088200 -11.18462400 -34.08327800
H 14.44395900 -10.23841700 -32.65403500
H 14.05376300 -9.50941100 -36.42737100
H 15.35789900 -10.60361500 -35.98969800
H 13.39684600 -8.42828200 -33.17600600
H 12.73613000 -9.06520000 -34.68347200
Ni 16.36807200 -8.10890000 -33.96693700

Ni^{2+}[NTA]$^{3-}$[HGATH] AlphaFold SAS

C 15.43199600 0.80092700 -29.77898100
C 16.11897500 -0.56218700 -29.65443700
O 17.27952900 -0.72148800 -29.28817900
H 14.60532400 0.62290700 -29.12341800
N 15.25264700 -1.59345600 -29.83580900
C 15.51855100 -3.03045300 -29.82871100
C 15.95499700 -3.66801400 -28.50395200
O 15.71009500 -4.86718300 -28.32062300
C 16.36794700 -3.47954000 -31.02352700
C 15.46851200 -3.56653400 -32.26339200
N 15.93588300 -4.32295200 -33.27258300
O 14.38443700 -2.99314700 -32.29044600
H 14.37680200 -1.37079900 -30.28180100
H 14.53359800 -3.46934600 -29.97726200
H 16.82196200 -4.45184800 -30.83020700
H 17.18248500 -2.77331000 -31.19150300
H 15.25876400 -4.74608800 -33.91603100
H 16.73438700 -4.90598300 -33.08244700
N 16.52987000 -2.89678500 -27.58447400
C 16.89780500 -3.35691600 -26.25399700
H 16.88313700 -1.99948700 -27.90229300
H 16.15063000 -3.99132100 -25.82507800
C 18.83906900 -12.26298600 -25.94105300
C 18.80562300 -11.35553300 -27.15233800
O 19.61331600 -11.51165200 -28.06277000
N 17.95520300 -10.29560500 -27.16359600
C 17.92696100 -9.40723100 -28.32177700
C 19.18967300 -8.53938000 -28.42975300

O	19.47958400	-7.97265900	-29.47372300
C	16.64749300	-8.55095300	-28.34117100
C	16.23946300	-8.19361700	-29.73171000
C	16.42700400	-8.84164400	-30.92003600
N	15.50885500	-7.06155300	-30.03383100
C	15.28806600	-7.04296400	-31.36445600
N	15.84188200	-8.10530600	-31.92608400
H	17.23578800	-10.22259600	-26.46277200
H	17.95790300	-10.03466600	-29.21149200
H	16.77766100	-7.64378600	-27.74612800
H	15.84664800	-9.12593500	-27.86475200
H	16.95955100	-9.74782800	-31.13455700
H	15.34473300	-6.27588400	-29.39497800
H	14.75956000	-6.28293100	-31.91344200
N	19.96990900	-8.45995700	-27.32963900
C	21.27954000	-7.85565500	-27.40253700
C	22.30045800	-8.62673600	-28.24229700
O	23.30683900	-8.04659600	-28.63866900
H	19.69406400	-8.96307100	-26.50447900
H	21.68001900	-7.75444900	-26.39433500
H	21.21544000	-6.85863500	-27.83551500
N	22.05798700	-9.92254500	-28.49476400
C	22.97140800	-10.69071700	-29.32750700
C	22.96656100	-10.23976500	-30.79472200
O	23.98471400	-10.33053500	-31.46230200
C	22.65899700	-12.18072900	-29.23137800
H	21.18540300	-10.35774600	-28.21637100
H	23.98744100	-10.49731500	-28.98703100
H	23.34970500	-12.73352200	-29.86627300
H	22.77266100	-12.52818300	-28.20412200
H	21.63822200	-12.39065400	-29.55003400
N	21.78730400	-9.78981600	-31.27904400
C	21.64120200	-9.35180700	-32.64627600
C	21.73023700	-7.83434100	-32.82519600
O	21.87438200	-7.37351600	-33.94841600
C	20.35816800	-9.93673500	-33.26579500
C	20.53549200	-11.40863700	-33.61887500
O	19.31555900	-9.75805700	-32.32808900
H	20.94150200	-9.84067500	-30.73021800
H	22.49711400	-9.73237300	-33.20190400
H	20.15222300	-9.36714100	-34.17861000
H	19.58296100	-11.81225500	-33.96194700
H	21.28083900	-11.53384000	-34.40768400
H	20.85395800	-11.97642800	-32.74249400
H	18.49277400	-10.15107700	-32.71675000
N	21.69481800	-7.06259100	-31.70220900
C	21.67918800	-5.62186000	-31.82969500
C	22.65791800	-4.84071300	-30.95485400
O	22.78387800	-3.63652700	-31.16197600
C	20.26021000	-5.04042800	-31.64801500
C	19.29448900	-5.68027400	-32.57057600
C	18.34830800	-6.62921100	-32.32742000
N	19.29223400	-5.50973000	-33.94285400
C	18.37379000	-6.33801700	-34.48239600
N	17.77323300	-7.01702400	-33.52160600
H	21.30271400	-7.48287100	-30.87042400
H	22.00264300	-5.42097300	-32.85032500
H	20.31738500	-3.96072700	-31.78992800
H	19.92248300	-5.22324200	-30.62646000
H	18.11367200	-7.09125200	-31.38823700
H	19.94225600	-4.94870700	-34.46657000
H	18.14627900	-6.43221100	-35.52811400
N	23.33870600	-5.49036400	-30.00009200
C	24.38300800	-4.78798500	-29.26498000
H	23.24093600	-6.48611700	-29.85124600
H	23.96573300	-4.02509600	-28.64149000
H	24.92042900	-5.51167700	-28.65743200
H	25.03329300	-4.34165800	-29.98804900
H	19.76436800	-11.98834300	-25.47930900
H	18.00913200	-12.14005500	-25.24913900
H	18.90646800	-13.26661600	-26.30583300
H	16.07694700	1.60239000	-29.43957700
H	15.07066100	0.96086100	-30.77337200
H	17.84475500	-3.90268500	-26.26529200
H	17.00502600	-2.47650700	-25.65552100
O	17.06893900	-10.64510300	-33.48738800
O	16.67378100	-8.14978700	-35.63392400
O	14.34814600	-6.37160500	-34.24160400
O	15.94767200	-12.24081700	-32.35781700
O	16.12400800	-9.03829700	-37.60687800
O	12.77675400	-6.93985300	-35.75786500
N	14.74212500	-9.35661000	-34.26043800
C	14.67540600	-10.53543900	-33.36186800
C	14.98260000	-9.80460000	-35.66056200
C	13.47609000	-8.57838500	-34.16926700
C	16.02996000	-11.22050100	-33.05035800
C	15.98496300	-8.93034200	-36.39805400
C	13.53866900	-7.16035700	-34.80847600
H	13.98697500	-11.27696500	-33.77695200
H	14.26500800	-10.21070900	-32.40814800
H	14.04448500	-9.84651800	-36.21408100
H	15.41655700	-10.80259600	-35.63199700
H	13.25611500	-8.45177000	-33.10964000
H	12.67174900	-9.15530100	-34.63183000
Ni	16.27026300	-8.19079600	-33.81084200
