# Supplementary figures and images for: The chromatin remodeler SMARCA5 binds to d-block metal supports: Characterization of affinities by IMAC chromatography and QM analysis
Source: PLoS One. 2024 Oct 7;19(10):e0309134. doi: 10.1371/journal.pone.0309134 (PMC11458017; doi:10.1371/journal.pone.0309134)

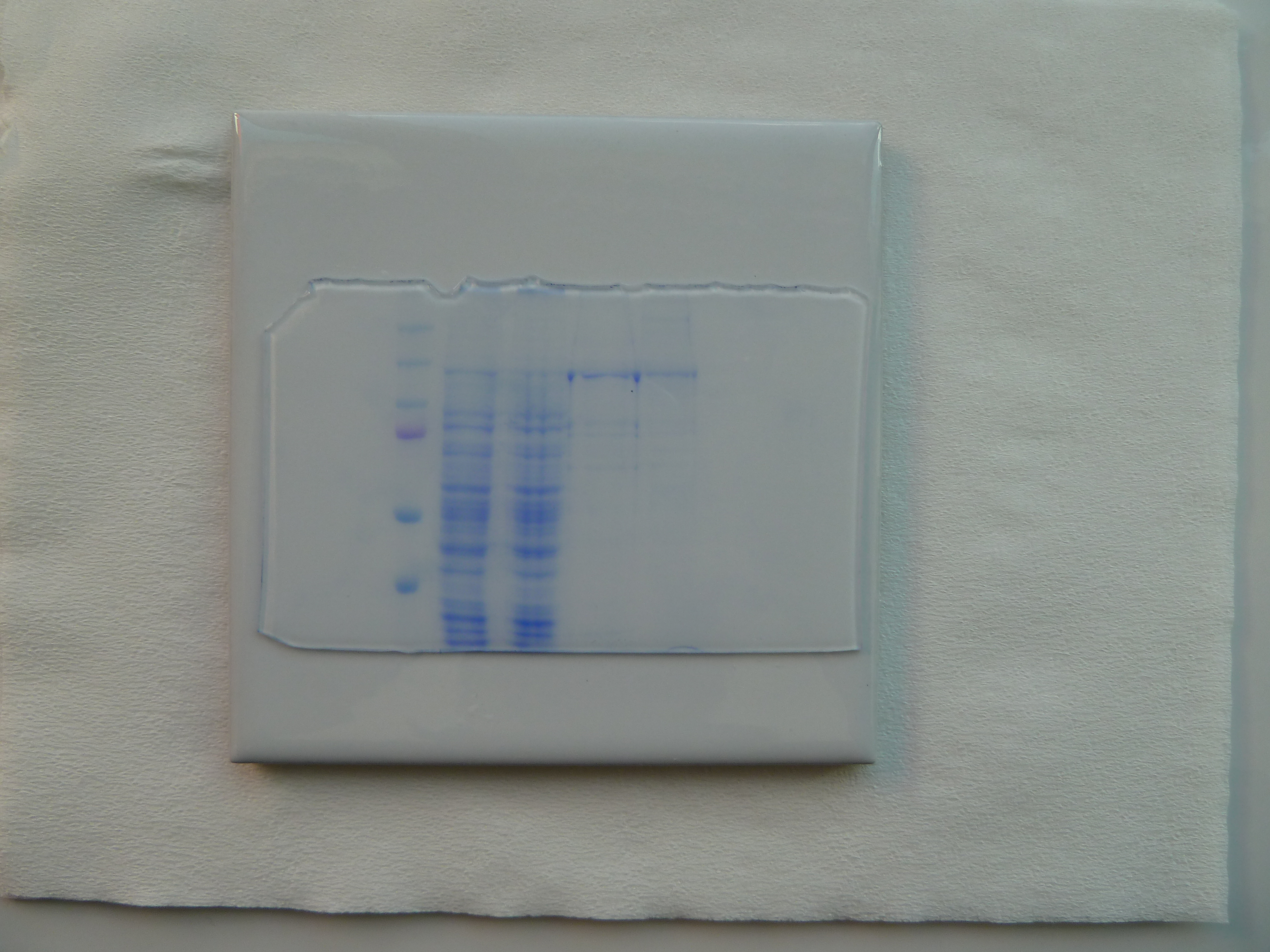

Supplement: S1 Raw images — (ZIP) [file pone.0309134.s002.zip › Figure 1A_unmodified.JPG]

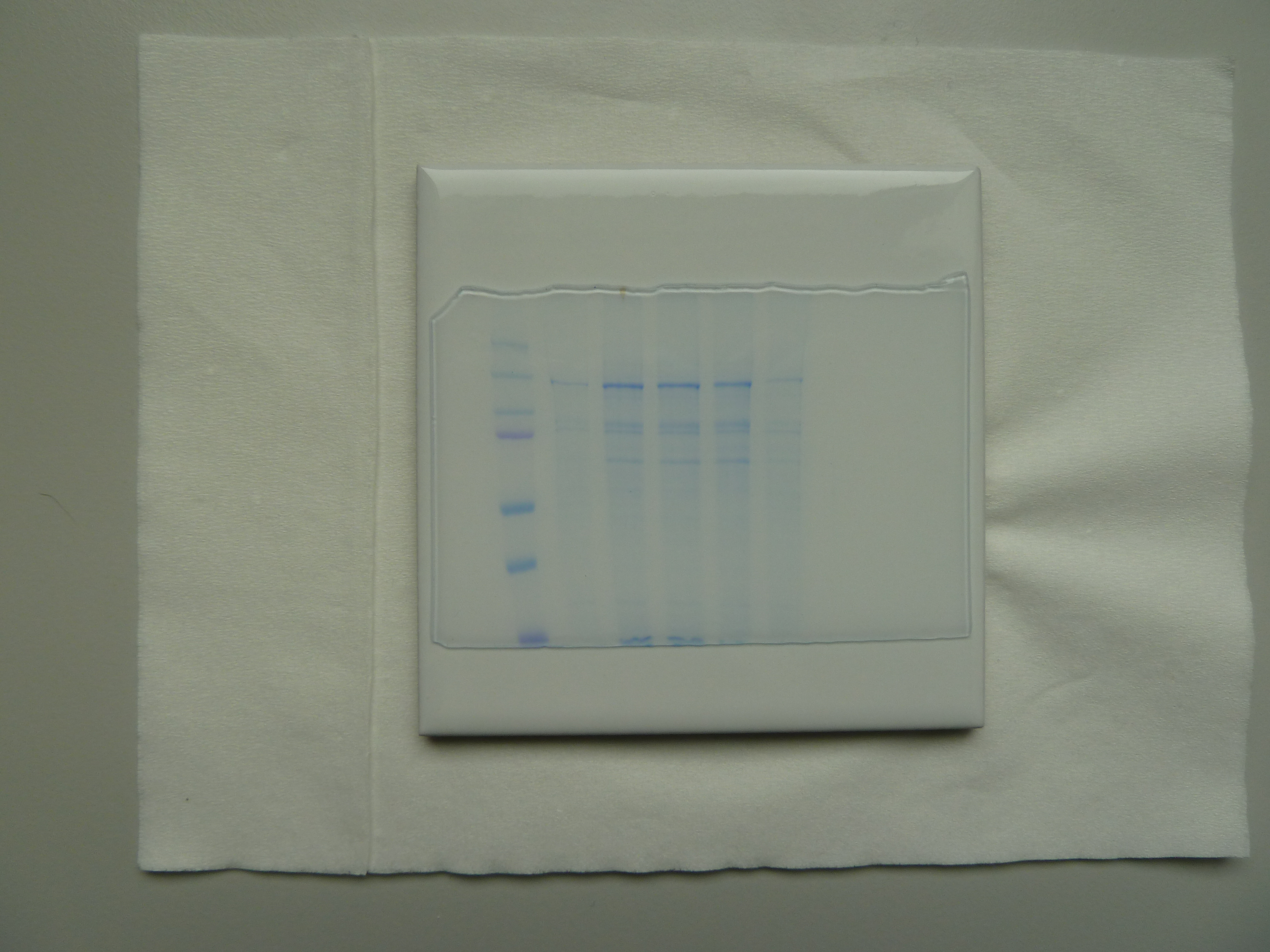

Supplement: S1 Raw images — (ZIP) [file pone.0309134.s002.zip › Figure 1B_unmodified.JPG]

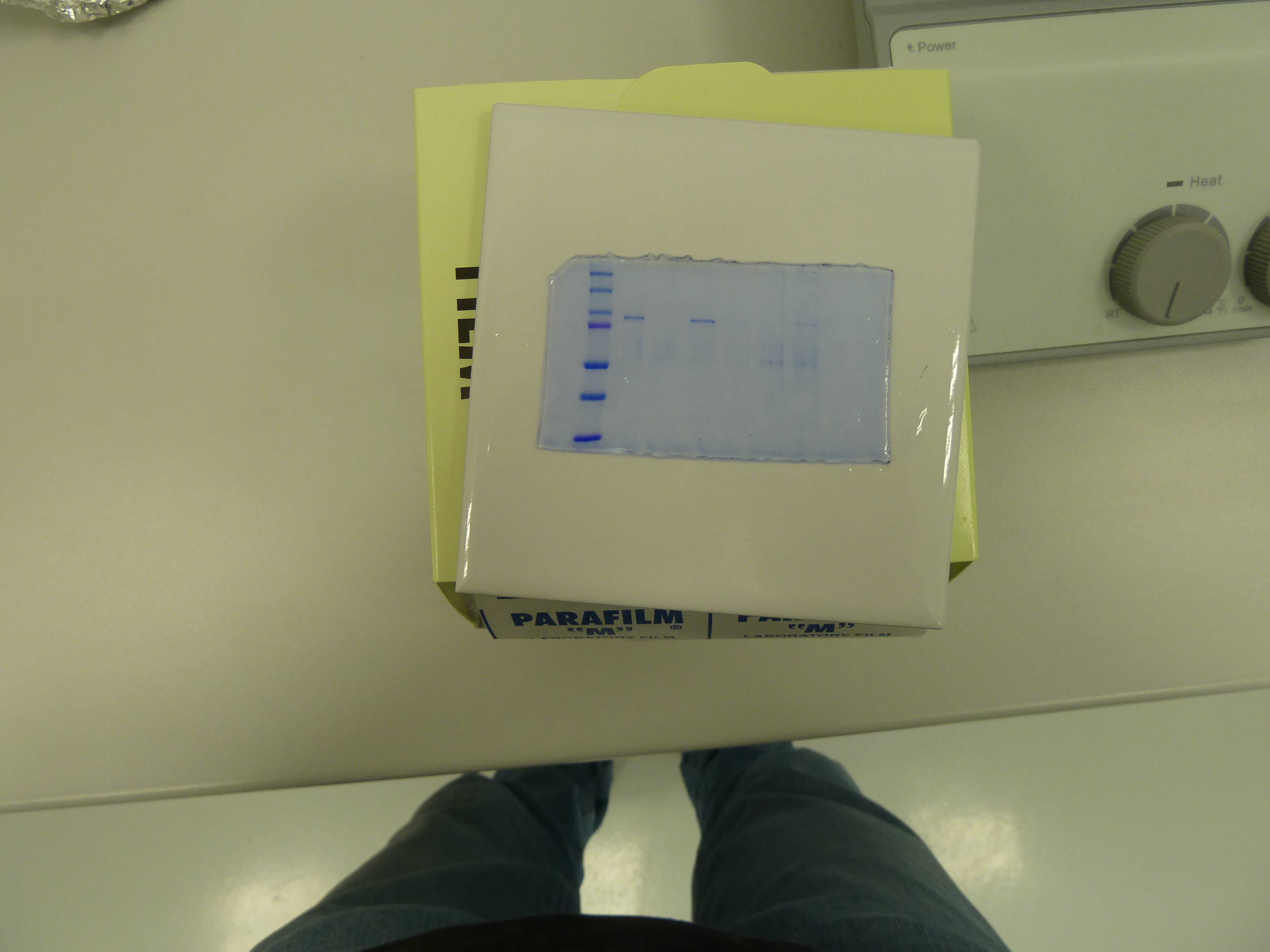

Supplement: S1 Raw images — (ZIP) [file pone.0309134.s002.zip › Figure 1D, 6xHis-TTF1_unmodified.JPG]

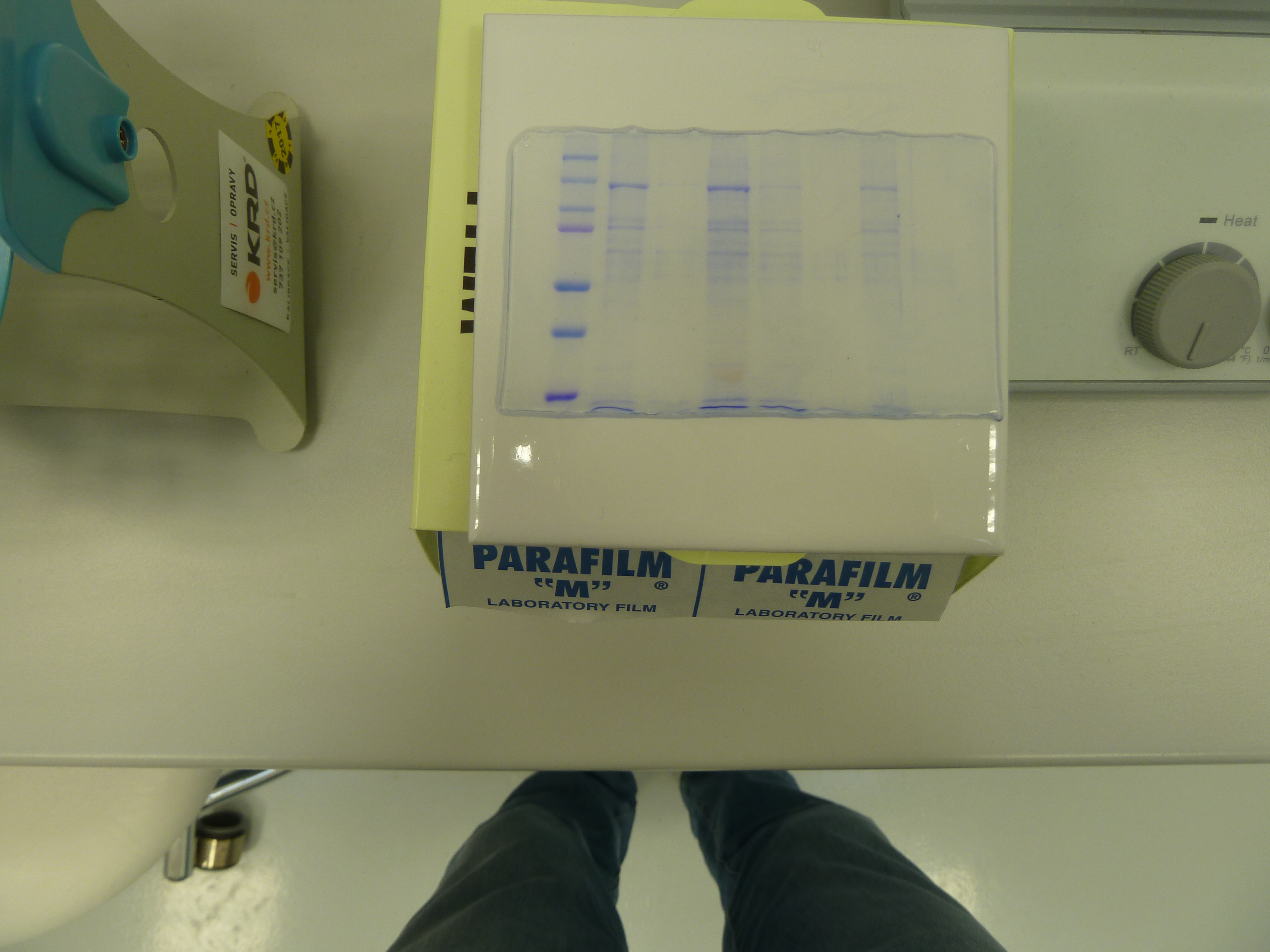

Supplement: S1 Raw images — (ZIP) [file pone.0309134.s002.zip › Figure 1D, SMARCA5_unmodified.JPG]

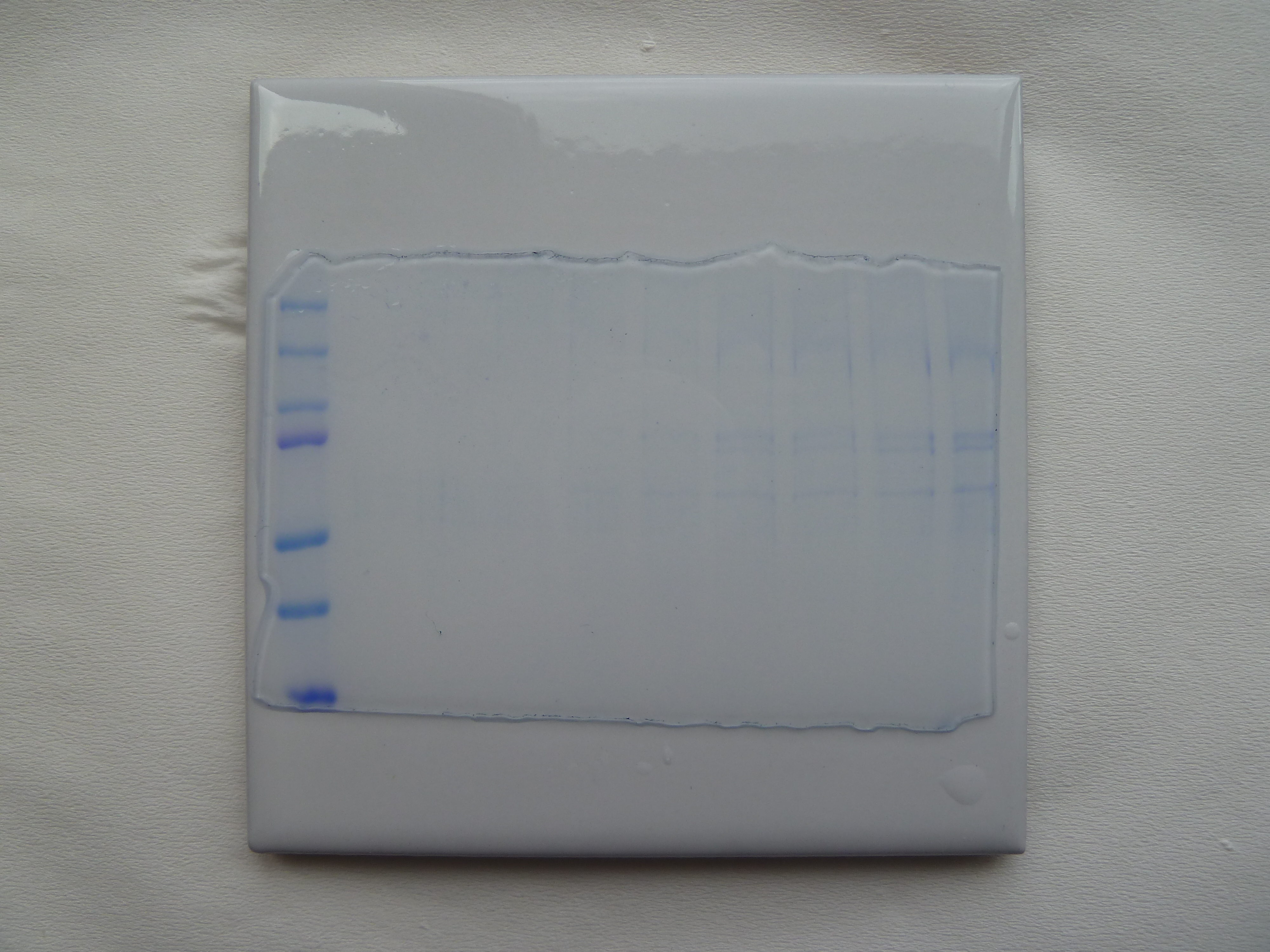

Supplement: S1 Raw images — (ZIP) [file pone.0309134.s002.zip › Figure 2A, Co2+_unmodified.JPG]

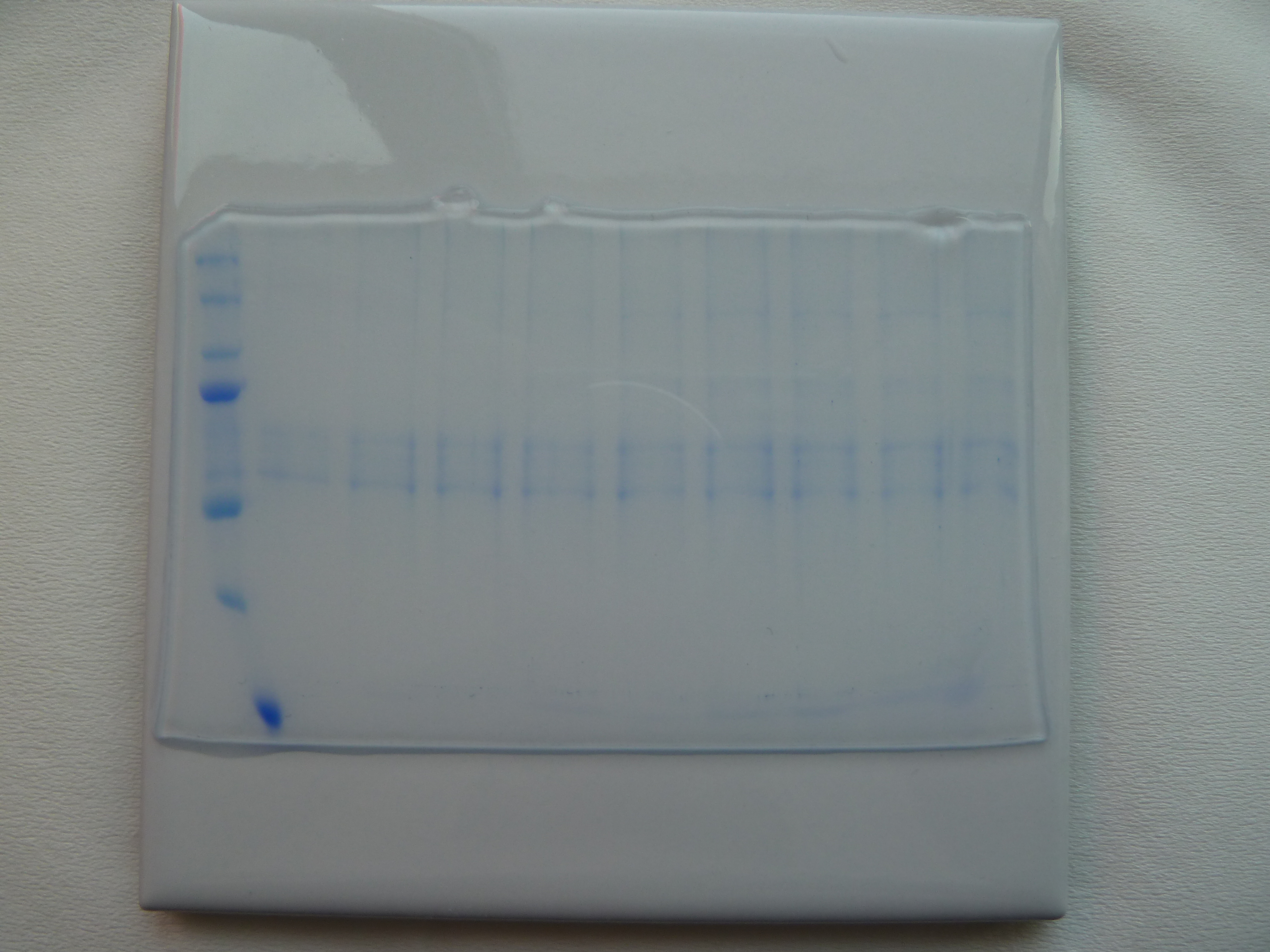

Supplement: S1 Raw images — (ZIP) [file pone.0309134.s002.zip › Figure 2A, Cu2+_unmodified.JPG]

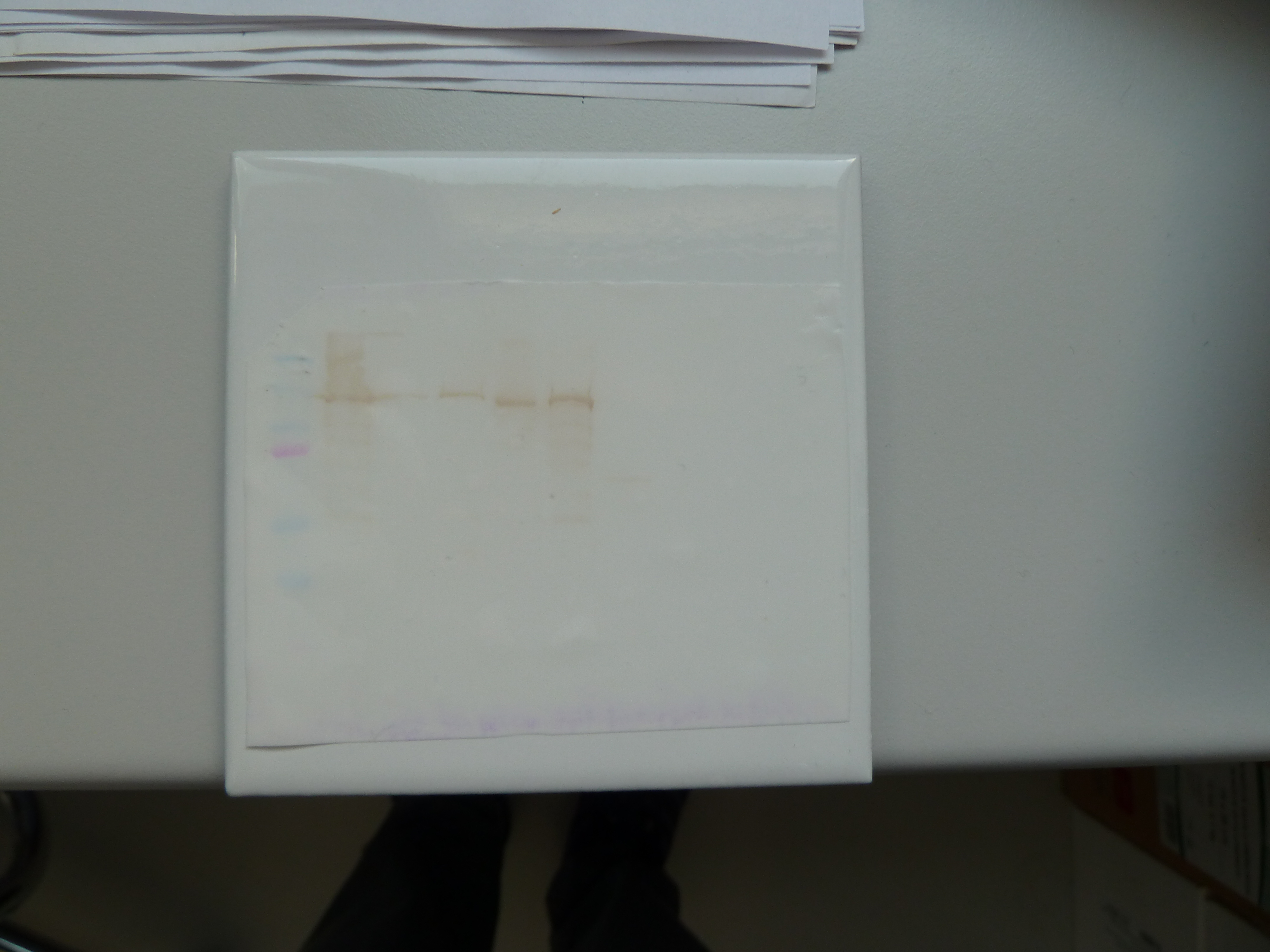

Supplement: S1 Raw images — (ZIP) [file pone.0309134.s002.zip › Figure S8_unmodified.JPG]

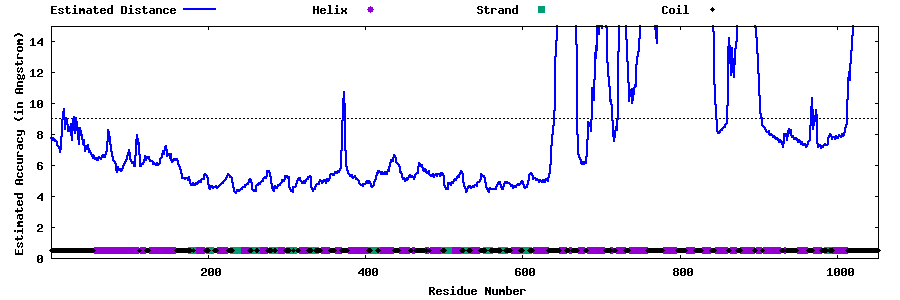

Supplement: S1 Data — (BZ2) [file pone.0309134.s003.bz2 › S751200_results/RSQ_3.png]

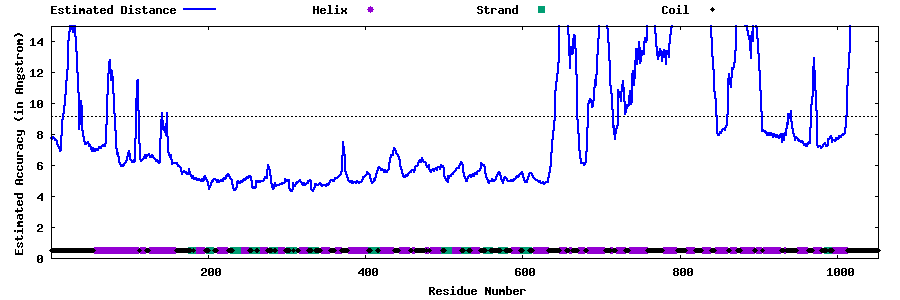

Supplement: S1 Data — (BZ2) [file pone.0309134.s003.bz2 › S751200_results/RSQ_4.png]

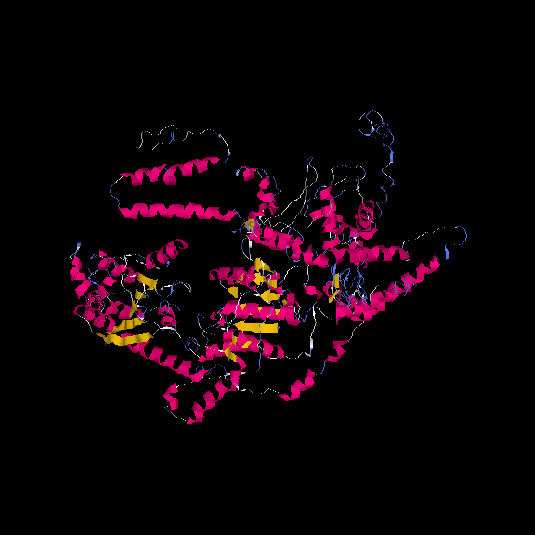

Supplement: S1 Data — (BZ2) [file pone.0309134.s003.bz2 › S751200_results/model2.gif]

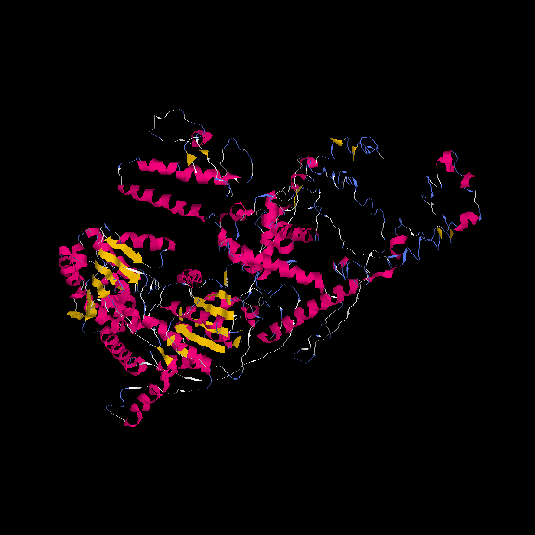

Supplement: S1 Data — (BZ2) [file pone.0309134.s003.bz2 › S751200_results/model5.gif]

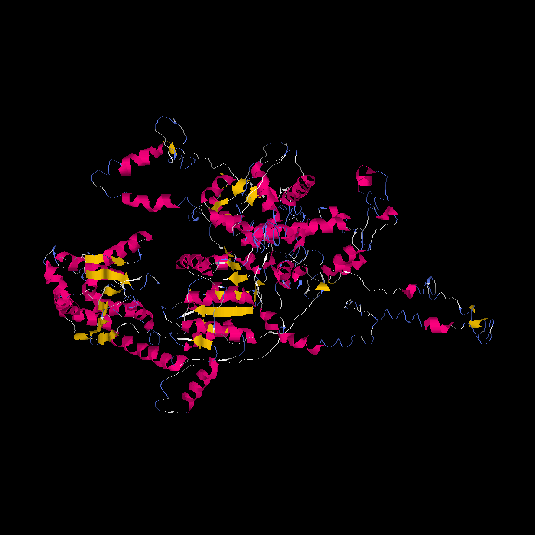

Supplement: S1 Data — (BZ2) [file pone.0309134.s003.bz2 › S751200_results/model4.gif]

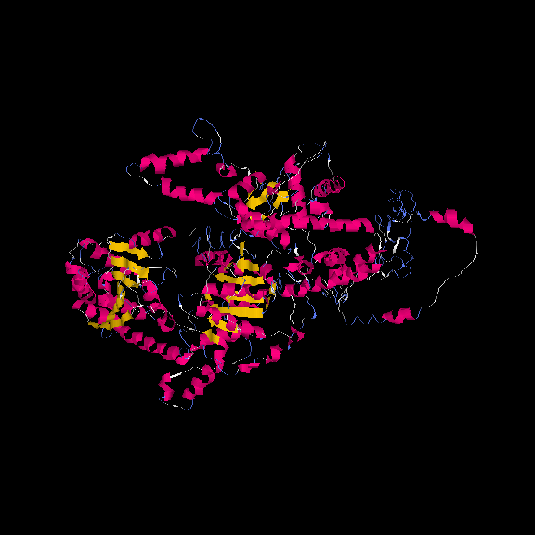

Supplement: S1 Data — (BZ2) [file pone.0309134.s003.bz2 › S751200_results/model3.gif]

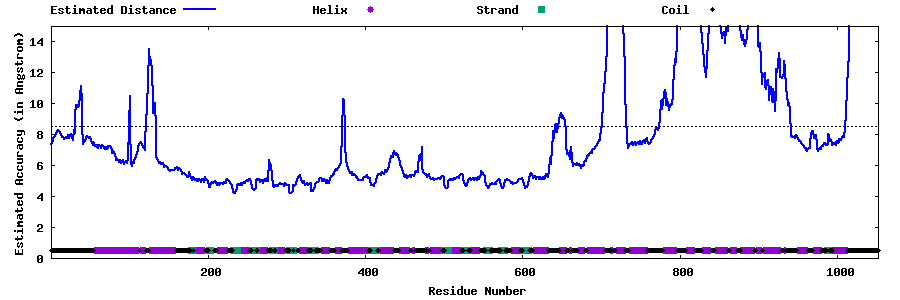

Supplement: S1 Data — (BZ2) [file pone.0309134.s003.bz2 › S751200_results/RSQ_5.png]

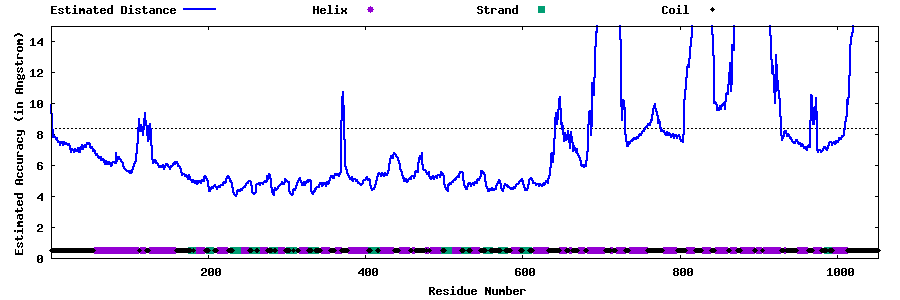

Supplement: S1 Data — (BZ2) [file pone.0309134.s003.bz2 › S751200_results/RSQ_2.png]

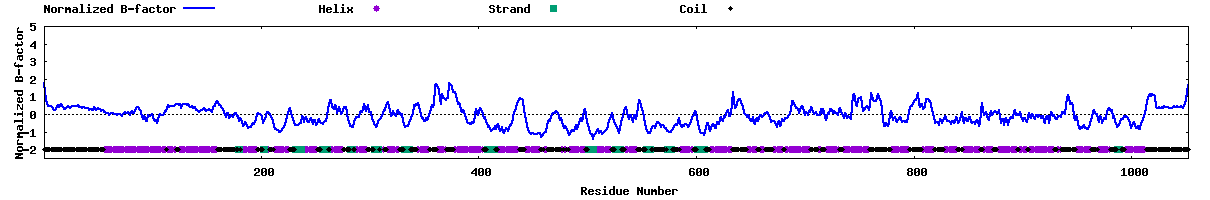

Supplement: S1 Data — (BZ2) [file pone.0309134.s003.bz2 › S751200_results/BFP.png]

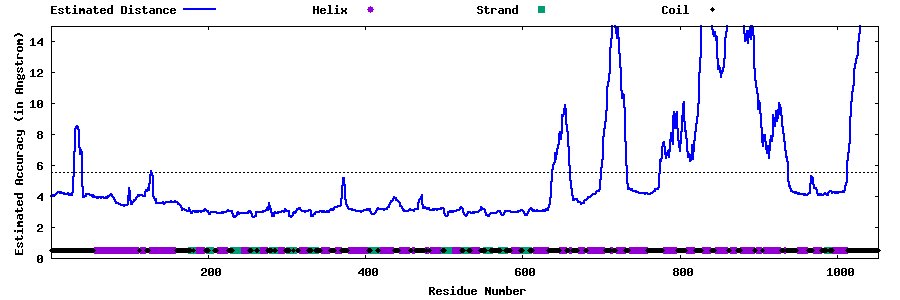

Supplement: S1 Data — (BZ2) [file pone.0309134.s003.bz2 › S751200_results/RSQ_1.png]

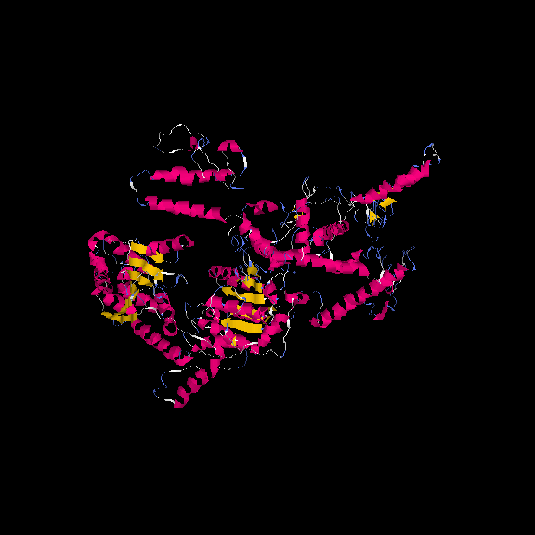

Supplement: S1 Data — (BZ2) [file pone.0309134.s003.bz2 › S751200_results/model1.gif]
